# Enhanced paddy leaf disease detection using novel dual metaheuristic loss functions in generative adversarial networks with identity block preservation for thermal image augmentation

**DOI:** 10.1038/s41598-026-36477-3

**Published:** 2026-02-15

**Authors:** Heba M. Khalil, Ahmed Elrefaiy, Mostafa Elbaz, Amira A. Elsonbaty

**Affiliations:** 1 Department of Computer Science, Faculty of Computers and Artificial Intelligence, Benha University, Benha, 13518 Egypt; 2 Department of Computer Science, Faculty of Computers and Informatics, Zagazig University, Zagazig, 44519 Egypt; 3 Field of Artificial Intelligence & Computer Engineering Technology, El Sewedy University of Technology, Cairo, Egypt; 4 Department of Computer Science, Faculty of Computers and Informatics, Kafrelsheikh University, Kafrelsheikh, Egypt; 5 Communication and Electronics Department, High Institute of Engineering and Technology, New Damietta, 34517 Egypt

**Keywords:** Generative adversarial networks, Metaheuristic optimization, Thermal image augmentation, Paddy disease detection, Chaoborus algorithm, Australian Crayfish algorithm, Identity block preservation, Missing pixel imputation, Adaptive connectivity, Data augmentation, Disease detection enhancement, Computational biology and bioinformatics, Engineering, Mathematics and computing

## Abstract

This paper presents a novel dual metaheuristic loss function framework integrated within Generative Adversarial Networks (GANs) for enhanced thermal image augmentation, specifically designed to improve paddy leaf disease detection through intelligent data quality enhancement and diversity generation. The proposed methodology revolutionizes traditional GAN training by replacing conventional loss functions with two bio-inspired metaheuristic algorithms: the Chaoborus algorithm, which serves as an innovative generator loss function implementing intelligent missing pixel imputation through phantom midge larvae hunting behavior simulation, and the Australian Crayfish algorithm, which functions as an advanced discriminator loss function optimizing adaptive 8-pixel connectivity through foraging and territorial behavior modeling. The framework incorporates strategically positioned identity blocks to preserve critical thermal signatures during adversarial training, ensuring disease-specific thermal patterns remain intact throughout the image enhancement process while maintaining diagnostic integrity. The proposed dual metaheuristic GAN achieves superior image generation quality with 31.47 ± 0.52 dB Peak Signal-to-Noise Ratio (PSNR) and 0.923 ± 0.008 Structural Similarity Index Measure (SSIM), representing significant improvements over state-of-the-art methods including StyleGAN2 (26.89 dB PSNR), Progressive GAN (27.34 dB PSNR), and BigGAN (28.12 dB PSNR). Disease classification performance evaluation across four distinct neural network architectures (ResNet-50, EfficientNet-B7, Vision Transformer, and DenseNet-201) reveals substantial accuracy improvements, with the Vision Transformer achieving 97.89 ± 0.63% accuracy using the proposed augmentation compared to 83.45 ± 1.76% on original datasets and 87.23 ± 1.54% with standard augmentation techniques. Statistical significance analysis confirms the robustness of improvements with p-values less than 0.001 for all comparative metrics and Cohen’s d effect sizes exceeding 1.2, indicating large practical significance. Rigorous tenfold cross-validation yields consistent performance with 96.85% mean accuracy and low standard deviation (0.674%), while Leave-One-Out Cross-Validation demonstrates minimal bias (< 0.0012) and low variance (< 0.0055). Generalization studies across five different datasets show robust transferability with direct transfer accuracies ranging from 84.12% to 91.45%, improving to 89.67–95.89% with minimal fine-tuning. Environmental robustness evaluation reveals excellent stability under varying temperature (15–35 °C), humidity (40–80%), and temporal conditions, with performance drops limited to 6.07% under extreme conditions. Comprehensive ablation studies validate the synergistic contribution of each framework component, with individual algorithms providing 5.68 dB and 3.76 dB PSNR improvements respectively, while their combination with identity blocks achieves the full 31.47 dB performance. Computational efficiency analysis demonstrates practical viability with 45.2 ± 2.8 ms inference time for image generation and 1.41 × speedup over baseline methods while maintaining 9.8 GB memory efficiency. Real-world field deployment across four geographic locations in India (Punjab, Tamil Nadu, West Bengal, and Odisha) over 2.75 months processing 44,860 images achieves 94.65% average accuracy with 3.12% false positive and 2.24% false negative rates.

## Introduction

Rice (*Oryza sativa* L.) serves as the primary staple food for over half of the global population, making accurate and early disease detection crucial for ensuring food security and sustainable agricultural production^1,2^. Traditional visual inspection methods for paddy disease identification are labor-intensive, subjective, and often detect diseases at advanced stages when treatment efficacy is significantly reduced^3^. The adoption of advanced imaging technologies, particularly thermal imaging, has emerged as a promising non-invasive approach for objective disease detection by capturing temperature variations associated with plant stress and pathological conditions^4^.

Recent advancements in thermal imaging for agricultural applications have demonstrated significant potential for early disease detection in various crops^5^. Thermal imaging enables the detection of diseases based on temperature variations before visible symptoms appear, offering a proactive approach to crop disease management. However, thermal image processing in agricultural applications encounters substantial challenges, particularly in missing pixel reconstruction due to environmental factors such as wind, humidity variations, and equipment limitations, as well as optimal pixel connectivity for accurate feature extraction^6^.

The integration of machine learning and deep learning techniques has revolutionized crop disease detection methodologies^7,8^. Deep learning applications for rice disease diagnosis have shown remarkable progress, with current trends focusing on improving accuracy and speed while addressing lightweight implementation requirements. Recent studies have demonstrated the effectiveness of convolutional neural networks (CNNs) and their variants in achieving high accuracy rates for paddy disease classification^9,10^. Hybrid CNN models integrating thermal imaging have achieved exceptional performance metrics with accuracy rates exceeding 99%, highlighting the potential of thermal-based approaches.

Generative Adversarial Networks (GANs) have gained significant attention in recent years for their ability to generate high-quality synthetic data, thereby addressing the critical challenge of limited datasets in agricultural applications^11^. GANs excel in generating synthetic data that can solve the problem of unavailability and limited datasets, making them particularly valuable for agricultural image processing where obtaining diverse, high-quality training data is often challenging. The application of GANs in image augmentation has shown promising results across various domains, including medical imaging and agricultural applications, by improving model generalization and performance.

However, conventional GAN loss functions, primarily based on adversarial and reconstruction losses, often fail to preserve critical domain-specific features essential for accurate disease classification in thermal imagery. This limitation is particularly pronounced in agricultural thermal imaging, where subtle temperature variations associated with disease symptoms must be maintained throughout the image generation process. Traditional loss functions may inadvertently smooth out or distort these critical thermal signatures, leading to reduced diagnostic accuracy in downstream classification tasks.

Metaheuristic optimization algorithms, inspired by biological and natural phenomena, have demonstrated exceptional performance in solving complex optimization problems across various domains^12–14^. Over the past three decades, more than 500 new metaheuristic algorithms have been proposed, with over 150 new algorithms emerging between 2019 and 2024 alone, indicating the sustained research interest and continuous innovation in this field. Nature-inspired metaheuristic optimization algorithms present themselves as effective alternatives to traditional gradient-based algorithms, having been extensively explored and rapidly finding applications in real-world systems.

Among the recently proposed metaheuristic algorithms, the Chaoborus algorithm, inspired by the predatory behavior of phantom midge larvae, offers unique characteristics suitable for pixel-level optimization tasks. The algorithm mimics the hunting patterns and survival strategies of Chaoborus species, which exhibit sophisticated prey detection and capture mechanisms in aquatic environments. These behavioral patterns translate effectively to missing pixel imputation problems, where the algorithm can systematically search for optimal pixel values while preserving spatial relationships.

The Crayfish Optimization Algorithm (COA), introduced in 2023, represents another innovative bio-inspired metaheuristic approach^15^. COA simulates crayfish’s summer resort behavior, competition behavior, and foraging behavior, divided into three different stages to balance exploration and exploitation. Recent developments have led to various enhanced versions of COA, including modified approaches that address convergence speed and local optimization challenges^16–18^. The integration of COA with other optimization techniques has yielded superior performance compared to individual methods, demonstrating its potential for hybrid optimization approaches.

The application of metaheuristic algorithms in image processing has gained considerable attention, with researchers exploring their potential in various image enhancement and restoration tasks^19^. Hybrid metaheuristic algorithms for image processing focus on the theory and application of metaheuristic algorithms for segmentation of images from different sources, presenting recent research on evolutionary, swarm, machine learning, and deep learning approaches. However, the integration of metaheuristic algorithms as loss functions within GAN architectures for agricultural thermal image processing remains largely unexplored.

Identity blocks, originally introduced in ResNet architectures, have proven effective in preserving important features during deep learning processes by enabling direct feature propagation through skip connections. In the context of thermal image processing for disease detection, identity blocks can play a crucial role in maintaining critical thermal signatures that are essential for accurate disease classification. The integration of identity blocks within GAN architectures can help preserve domain-specific features while allowing for effective image enhancement and augmentation.

The challenge of limited thermal imaging datasets in agricultural applications necessitates innovative approaches to data augmentation that not only increase data volume but also enhance data quality through intelligent optimization strategies. Current augmentation techniques often rely on simple geometric transformations or intensity variations, which may not capture the complex thermal patterns associated with different disease states in paddy leaves.

This work addresses these limitations by proposing a novel framework that integrates dual metaheuristic optimization algorithms as loss functions within a GAN architecture specifically designed for thermal image augmentation in paddy disease detection. The Chaoborus algorithm serves as an innovative generator loss function for intelligent missing pixel imputation, while the Australian Crayfish algorithm (a variant of COA specifically adapted for Australian crayfish behaviors) functions as a discriminator loss function for optimizing adaptive 8-pixel connectivity. The integration of identity blocks ensures the preservation of critical thermal signatures throughout the adversarial training process.

The primary contributions of this research include: (1) the introduction of novel bio-inspired loss functions for GAN training in agricultural thermal imaging; (2) the development of an adaptive connectivity optimization framework using crayfish-inspired algorithms; (3) the integration of identity preservation mechanisms for maintaining disease-specific thermal patterns; (4) comprehensive validation of the proposed approach on diverse paddy disease datasets; and (5) demonstration of significant improvements in downstream disease detection model performance through enhanced thermal image augmentation.

The remainder of this paper is organized as follows: Sect. "Related work and background" presents the related work and theoretical background; Sect. "Materials and methods" describes the proposed methodology in detail; Sect. "Results and analysis" presents the experimental setup and results; Sect. "Discussion" discusses the findings and implications; and Sect. "Conclusion and future work" concludes the paper with future research directions.

## Related work and background

This section provides a comprehensive review of existing GAN architectures and loss functions, establishing the foundation for the proposed dual metaheuristic framework. The analysis focuses on architectural innovations, loss function developments, and their applications in image processing tasks.

### Generative adversarial network architectures

The evolution of GAN architectures has been marked by significant innovations addressing fundamental challenges in generative modeling. Since the introduction of the original GAN framework in 2014^20^, numerous architectural variants have emerged, each targeting specific limitations and application domains. Table 1 presents the complete hyperparameter configuration for the dual metaheuristic GAN framework, including learning rates, batch size, and loss function weights optimized through systematic grid search.

**Table 1:** Hyperparameter configuration for dual metaheuristic GAN framework.

Architecture	Key innovation	Generator features	Discriminator features	Image resolution	Primary applications
Vanilla GAN	Adversarial training	Fully connected layers	Binary classification	Low (32 × 32)	Basic image generation
DCGAN	Convolutional architecture	Transposed convolutions, BatchNorm	Strided convolutions, LeakyReLU	Medium (64 × 64)	Stable image synthesis
WGAN	Wasserstein distance	Standard DCGAN structure	Critic without sigmoid	Medium (64 × 64)	Stable training dynamics
LSGAN	Least squares loss	DCGAN-based architecture	Regression-based output	Medium (64 × 64)	High-quality generation
Progressive GAN	Progressive growing	Layer-by-layer training	Synchronized growth	High (1024 × 1024)	High-resolution synthesis
StyleGAN	Style-based generator	Mapping network + synthesis	Standard discriminator	Very High (1024 × 1024)	Controllable generation
StyleGAN2	Weight demodulation	Improved architecture	Enhanced discriminator	Very High (1024 × 1024)	Superior quality control
BigGAN	Large-scale training	Class-conditional BatchNorm	Spectral normalization	High (512 × 512)	Large dataset generation
CycleGAN	Cycle consistency	Encoder-decoder structure	PatchGAN discriminator	Medium (256 × 256)	Unpaired translation
Pix2Pix	Conditional translation	U-Net architecture	PatchGAN discriminator	Medium (256 × 256)	Paired translation

Deep Convolutional GANs (DCGAN)^21^ introduced convolutional architectures that significantly improved training stability and image quality compared to the original fully-connected GANs. The architecture employs transposed convolutions in the generator and strided convolutions in the discriminator, establishing design principles that remain influential in modern GAN architectures. Wasserstein GANs (WGAN)^22^ addressed training instability by replacing the Jensen-Shannon divergence with the Wasserstein distance, providing more meaningful gradients and reducing mode collapse issues. The architecture maintains the DCGAN structure while implementing the critic function instead of a traditional discriminator. Least Squares GANs (LSGAN)^23^ tackled the vanishing gradient problem by employing least squares loss functions instead of binary cross-entropy. This modification forces the generator to produce samples closer to the decision boundary, resulting in higher quality images and more stable training dynamics. Progressive GANs^24^ revolutionized high-resolution image generation through layer-by-layer training, starting from low resolution (4 × 4) and progressively adding layers to reach resolutions up to 1024 × 1024. This approach enables stable training of very deep networks while maintaining high image quality. StyleGAN^25,26^ introduced a novel style-based architecture that separates content from style through a mapping network, enabling unprecedented control over generated image attributes. The synthesis network receives style vectors at multiple resolutions, allowing fine-grained manipulation of image characteristics. BigGAN^27^ demonstrated the potential of large-scale GAN training, incorporating self-attention mechanisms and spectral normalization to achieve state-of-the-art results on large datasets. The architecture employs class-conditional batch normalization and large batch sizes to improve training stability and image quality. CycleGAN^28^ addressed the limitation of requiring paired data by introducing cycle consistency loss, enabling translation between unpaired domains. The architecture employs two generators and two discriminators, with cycle consistency ensuring that images translated to another domain and back retain their original characteristics. Pix2Pix^29^ established the foundation for image-to-image translation using paired training data. The architecture employs a U-Net generator with skip connections and a PatchGAN discriminator that operates on local image patches rather than entire images. This design enables high-quality translations while preserving spatial relationships.

### Conditional and translation-based architectures

Conditional GANs^30^ extended the basic GAN framework by incorporating additional information to guide the generation process. These architectures enable controlled synthesis based on labels, images, or other conditioning inputs. The experimental environment specifications are detailed in Table 2, including GPU architecture, memory capacity, processor specifications, and software dependencies.

**Table 2:** Hardware and software specifications for experimental setup.

Architecture	Conditioning type	Translation method	Dataset requirements	Cycle consistency	Loss components
CGAN	Class labels	N/A	Labeled data	No	Adversarial + Classification
ACGAN	Class labels	N/A	Labeled data	No	Adversarial + Auxiliary
InfoGAN	Latent codes	N/A	Unlabeled data	No	Adversarial + Mutual information
Pix2Pix	Input image	Paired mapping	Paired images	No	Adversarial + L1 reconstruction
CycleGAN	Domain mapping	Unpaired translation	Unpaired images	Yes	Adversarial + Cycle consistency
DiscoGAN	Domain mapping	Unpaired translation	Unpaired images	Yes	Adversarial + Reconstruction (L2)
BicycleGAN	Input + noise	Diverse mapping	Paired images	No	Adversarial + L1 + KL divergence

### Loss function evolution in GANs

The development of effective loss functions has been crucial for GAN advancement, addressing issues such as training instability, mode collapse, and gradient vanishing. Modern GAN implementations employ sophisticated loss formulations that balance adversarial objectives with additional constraints. Table 3 shows the stratified distribution of 636 thermal images across training (70%), validation (15%), and test (15%) sets for all six disease categories.

**Table 3:** Training, validation, and test set distribution across disease categories.

Loss function	Mathematical foundation	Generator objective	Discriminator objective	Stability	Computational cost	Mode collapse resistance
Minimax loss	Jensen-Shannon divergence	min log(1-D(G(z)))	max log(D(x)) + log(1-D(G(z)))	Low	Low	Weak
Non-saturating loss	Modified JS divergence	max log(D(G(z)))	max log(D(x)) + log(1-D(G(z)))	Medium	Low	Weak
Wasserstein loss	Earth mover distance	min D(G(z))	max D(x)—D(G(z))	High	Medium	Strong
WGAN-GP	Wasserstein + Gradient penalty	min D(G(z))	max D(x)—D(G(z))—λ·GP	High	High	Strong
Least squares loss	Pearson χ^2^ Divergence	min (D(G(z))—c)^2^	min (D(x)—b)^2^ + (D(G(z))—a)^2^	Medium	Low	Medium
Hinge loss	Margin-based objective	min -D(G(z))	min max(0, 1-D(x)) + max(0, 1 + D(G(z)))	Medium	Low	Medium
Relativistic loss	Relative discrimination	min D(x)—D(G(z))	max D(x)—D(G(z))	Medium	Low	Medium
GANetic loss	Genetic programming	Evolved function	Evolved function	High	Medium	Strong

### Advanced GAN variants and recent developments

Modern GAN research has focused on scaling to higher resolutions, improving training efficiency, and addressing specific application requirements. Recent architectures incorporate attention mechanisms, progressive training strategies, and novel normalization techniques. Table 4 demonstrates the fivefold expansion of the training set from 445 original images to 2,670 total images including synthetic variations.

**Table 4:** Data augmentation strategy and synthetic image generation.

Architecture	Innovation	Resolution	Training stability	Computational efficiency	Unique features
StyleGAN3	Alias-free architecture	1024 × 1024	High	Medium	Rotation/translation equivariance
GigaGAN	Scalable ultra-high resolution	4096 × 4096	High	High	Fast generation, multi-scale
SparseGAN	Sparse network design	1024 × 1024	Medium	Very High	Reduced computational overhead
ISFB-GAN	Improved stability	512 × 512	Very high	Medium	Enhanced convergence properties
DGL-GAN	Discriminator-guided learning	Variable	High	High	Knowledge distillation approach

### Application-specific GAN developments

Recent research has focused on domain-specific GAN applications, particularly in medical imaging, agricultural monitoring, and specialized image processing tasks. These developments highlight the need for tailored loss functions and architectures. To address the class imbalance problem, inversely proportional class weights were computed as shown in Table 5, with Leaf Folder receiving the highest weight (6.47) and Bacterial Leaf Blight serving as the baseline (1.00). Table 5 presents the class weights for addressing dataset imbalance.

**Table 5:** Class weights for addressing dataset imbalance.

Application domain	Architecture used	Loss function	Key challenges	Performance metrics	Success factors
Medical imaging	DCGAN, StyleGAN, SPADE	MSE, Perceptual, Adversarial	Data scarcity, privacy	FID: 15–40, Dice: 0.85–0.90	Domain knowledge integration
Agricultural monitoring	DCGAN, Pix2Pix	L1, Adversarial	Environmental variations	Accuracy: 85–95%	Feature preservation
Thermal imaging	Modified DCGAN	Custom thermal loss	Missing pixels, noise	PSNR: 25–35 dB	Temperature signature preservation
X-ray analysis	DCGAN, ACGAN	Adaptive normalization	Mode collapse, diversity	IS: 6–8, FID: 20–35	Preprocessing techniques
Renewable energy	Penca-GAN	Pancreas-inspired loss	Limited datasets	SSIM: 0.85–0.92	Bio-inspired optimization

Medical imaging applications have shown that simpler models such as DCGAN, LSGAN, and WGAN perform systematically poorly despite intensive hyperparameter optimization, while more sophisticated architectures like StyleGAN and SPADE achieve superior results.

Agricultural applications face unique challenges related to environmental variations and the need to preserve biologically relevant features. Traditional augmentation techniques often fail to maintain critical characteristics, necessitating specialized approaches.

### Research gaps and limitations

The literature review reveals several important limitations in current GAN architectures and loss functions:

1. While metaheuristic algorithms have shown success in optimization problems, their integration as loss functions within GAN architectures remains largely unexplored.
2. Current loss functions often fail to preserve critical domain-specific features, particularly in specialized applications like thermal imaging for disease detection.
3. The potential of bio-inspired optimization for GAN training has not been fully exploited, despite success in other optimization domains.
4. Most existing approaches employ static loss functions, missing opportunities for dynamic adaptation based on training progress and data characteristics.
5. Current architectures inadequately address the preservation of critical features during adversarial training, particularly important for diagnostic applications.

These limitations provide the motivation for the proposed dual metaheuristic GAN framework, which addresses these gaps through innovative integration of bio-inspired optimization algorithms with advanced deep learning architectures.

## Materials and methods

This section presents the comprehensive methodology for developing the dual metaheuristic GAN framework for thermal image augmentation in paddy leaf disease detection. The approach integrates novel bio-inspired optimization algorithms with advanced deep learning architectures to address critical challenges in agricultural thermal imaging.

### Dataset and experimental setup

The experimental validation utilized a specialized thermal imaging dataset of paddy leaf diseases obtained from the School of Information Technology & Engineering, Vellore Institute of Technology (VIT), Vellore, Tamil Nadu, India, publicly available on Kaggle at https://www.kaggle.com/datasets/sujaradha/thermal-images-diseased-healthy-leaves-paddy. The dataset comprises 636 thermal images captured using a FLIR E8 thermal camera with infrared resolution of 320 × 240 pixels (76,800 pixels), temperature accuracy of ± 2%, field of view of 45° × 34°, and thermal sensitivity below 0.06°C at 30°C. Images were acquired during morning hours (8:00–10:00 AM) under controlled environmental conditions with ambient temperatures between 25–30 °C and relative humidity between 60–70%, maintaining a standardized camera distance of 30–50 cm perpendicular to the leaf surface. Comprehensive camera calibration procedures were performed before each session using reference standards to ensure measurement accuracy. All images underwent preprocessing including thermal calibration based on ambient conditions, adaptive Gaussian filtering for noise reduction while preserving edge information, adaptive histogram equalization for contrast enhancement, artifact removal to correct reflection and emissivity variations, and standardization to consistent temperature ranges (0–100 °C) and spatial resolutions suitable for neural network processing.

The dataset exhibits intentional class imbalance reflecting natural disease prevalence patterns, distributed across six categories: Bacterial Leaf Blight (220 images, 34.6%), Blast (67 images, 10.5%), Leaf Spot (80 images, 12.6%), Leaf Folder (34 images, 5.3%), Hispa (142 images, 22.3%), and Healthy Leaves (93 images, 14.6%). A stratified train-validation-test split strategy allocated 70% of samples to training (445 images), 15% to validation (95 images), and 15% to testing (96 images), maintaining class distribution proportions within each subset. For Bacterial Leaf Blight: 154 training, 33 validation, 33 testing; Blast: 47 training, 10 validation, 10 testing; Leaf Spot: 56 training, 12 validation, 12 testing; Leaf Folder: 24 training, 5 validation, 5 testing; Hispa: 99 training, 22 validation, 21 testing; Healthy: 65 training, 14 validation, 14 testing. Random seed control (seed = 42) ensured reproducible partitioning across all experiments. The validation set guided hyperparameter selection, early stopping, and architectural choices during development, while the testing set remained completely isolated for final unbiased performance evaluation. Comprehensive cross-validation procedures included tenfold cross-validation with stratified folds and Leave-One-Out Cross-Validation (LOOCV) across all 636 samples to provide robust performance estimates with minimal bias, as reported in Sect. "Cross-validation and model validation studies". Training employed class weights inversely proportional to class frequencies to address imbalance, ensuring equal prioritization of all disease categories during model optimization. Data augmentation using the proposed dual metaheuristic GAN was applied exclusively to the training set, while validation and testing sets contained only original captured images to ensure unbiased evaluation of real-world classification performance. Table 6 provides comprehensive dataset characteristics including thermal camera specifications (FLIR E8, 320 × 240 pixels, ± 2% accuracy), image acquisition protocols, disease distribution, preprocessing steps, and cross-validation design.

**Table 6:** Comprehensive dataset characteristics and thermal image specifications.

Characteristic	Details
Dataset source	
Primary source	School of Information Technology & Engineering, VIT, Vellore, Tamil Nadu, India
Acquisition specifications	
Thermal camera	FLIR E8
Infrared resolution	320 × 240 pixels (76,800 pixels)
Temperature accuracy	± 2% (10–35 °C ambient, > 0 °C object)
Field of view	45° × 34°
Thermal sensitivity	< 0.06°C @ 30°C
Spectral range	7.5–14 μm
Image format	Radiometric JPEG
Collection protocol	
Collection time	8:00–10:00 AM (morning hours)
Ambient temperature	25–30°C (controlled)
Relative humidity	60–70% (controlled)
Camera distance	30–50 cm from leaf surface
Camera orientation	Perpendicular to leaf surface
Calibration	Before each session using reference standards
Dataset composition	
Total images	636
Number of classes	6 (5 disease categories + 1 healthy)
Class distribution	
Bacterial leaf blight	220 images (34.6%)—Elevated temperature zones, high economic impact
Blast	67 images (10.5%)—Irregular thermal patterns, severe yield reduction
Leaf spot	80 images (12.6%)—Localized hot spots, moderate damage
Leaf folder	34 images (5.3%)—Linear thermal variations, insect-induced damage
Hispa	142 images (22.3%)—Scattered thermal anomalies, pest-related damage
Healthy leaves	93 images (14.6%)—Uniform thermal distribution, control samples
Class imbalance ratio	6.47:1 (max:min class sizes)
Train-validation-test split	
Overall distribution	Training: 70% (445 images), Validation: 15% (95 images), Testing: 15% (96 images)
Bacterial leaf blight split	Train: 154 (70%), Val: 33 (15%), Test: 33 (15%)
Blast split	Train: 47 (70%), Val: 10 (15%), Test: 10 (15%)
Leaf spot split	Train: 56 (70%), Val: 12 (15%), Test: 12 (15%)
Leaf folder split	Train: 24 (70%), Val: 5 (15%), Test: 5 (15%)
Hispa split	Train: 99 (70%), Val: 22 (15%), Test: 21 (15%)
Healthy leaves split	Train: 65 (70%), Val: 14 (15%), Test: 14 (15%)
Split strategy	Stratified random sampling maintaining class proportions
Random seed	42 (fixed for reproducibility)
Validation purpose	Hyperparameter tuning, early stopping, model selection
Testing purpose	Final unbiased performance evaluation only
Cross-validation design	
10-fold CV	Stratified folds, 63–64 images per fold, results in Sect. "K-fold cross-validation performance assessment"
10-fold CV Results	Mean Accuracy: 96.85%, Standard deviation: 0.674%
10-fold CV computational cost	67.2 GPU hours
Leave-one-out CV	636 iterations, minimal bias estimation, results in Sect. "Leave-one-out cross-validation analysis"
LOOCV results	Accuracy: 96.79%, Precision: 96.08%, Recall: 97.51%, F1-score: 0.968
LOOCV bias and variance	Bias: < 0.0012, Variance: < 0.0055
Data preprocessing	
Thermal calibration	Normalized based on ambient conditions per session
Noise reduction	Adaptive Gaussian filtering preserving edge information
Contrast enhancement	Adaptive histogram equalization for thermal patterns
Artifact removal	Correction of reflection and emissivity variations
Temperature range	Standardized to 0–100°C
Spatial resolution	Standardized to 256 × 256 pixels for neural network input
Class balancing strategy	
Training weights	Inversely proportional to class frequencies
Bacterial leaf blight weight	1.00 (baseline, most frequent)
Blast weight	3.28 (220/67)
Leaf spot weight	2.75 (220/80)
Leaf folder weight	6.47 (220/34, highest weight)
Hispa weight	1.55 (220/142)
Healthy leaves weight	2.37 (220/93)
Data augmentation	
Augmentation method	Proposed dual metaheuristic GAN framework
Applied to	Training set only (445 images)
Validation/Test sets	Original images only (no augmentation)
Augmentation techniques	GAN-based thermal image generation with identity block preservation
Dataset statistics	
Mean image resolution	320 × 240 pixels (standardized to 256 × 256 for processing)
Temperature range	0–100°C (calibrated)
Mean thermal contrast	4.73°C (std dev within images)
Missing pixels (Average)	3.2% per image
Missing pixels (Range)	0–15% (threshold: max 15%)
Images with < 5% missing	78% of dataset
Images with 5–10% missing	18% of dataset
Images with 10–15% missing	4% of dataset
Thermal characteristics by class	
Healthy leaves (Mean Std Dev)	2.14 °C (most uniform)
Bacterial leaf blight (Mean Std Dev)	6.38 °C (highest variability, systemic infection)
Blast (Mean Std Dev)	5.21 °C (intermediate variability)
Leaf spot (Mean Std Dev)	4.89 °C (moderate variability)
Leaf folder (Mean Std Dev)	3.97 °C (localized damage)
Hispa (Mean Std Dev)	4.52 °C (scattered damage)
Thermal gradient analysis	
Healthy leaves (Mean gradient)	0.73 °C/cm (baseline)
Leaf folder (Mean gradient)	1.45 °C/cm (lowest among diseases)
Blast (Mean gradient)	2.89 °C/cm (highest, sharp boundaries)
Other diseases (Mean gradient range)	1.67–2.34 °C/cm
Quality assurance	
Excluded images	Images with severe blur, saturation, or > 15% missing data
Quality criteria	Adequate thermal contrast, proper focus, minimal artifacts
Final dataset quality	636 high-quality thermal images suitable for algorithm development
Reproducibility	
Data availability	Public (Kaggle repository)
Code availability	To be provided upon manuscript acceptance
Configuration files	YAML format with all preprocessing parameters
Random seeds	Fixed (seed = 42) for all partitioning and augmentation

#### Experimental setup and implementation configuration

The experimental validation was conducted on a high-performance deep learning workstation specifically configured for intensive GAN training and thermal image processing. The hardware infrastructure comprised an NVIDIA RTX 4090 GPU with 24 GB GDDR6X VRAM, 16,384 CUDA cores, and 2.52 GHz boost clock for parallel processing of high-resolution thermal images and dual metaheuristic optimization. The CPU subsystem utilized an Intel Core i9-12900K processor with 16 cores (8 performance cores and 8 efficiency cores), 24 threads, operating at 3.2 GHz base frequency with turbo boost up to 5.2 GHz for data preprocessing, augmentation pipeline execution, and metric calculation. System memory comprised 64 GB of DDR4-3200 RAM in dual-channel configuration with CL16 latency, supporting large batch loading and concurrent data processing operations. Storage infrastructure employed a 2 TB NVMe SSD with PCIe 4.0 × 4 interface, achieving 7000 MB/s read and 5000 MB/s write speeds to minimize I/O bottlenecks during training. The power supply was a 1000W 80 + Platinum modular unit with greater than 90% efficiency, and thermal management employed custom liquid cooling with GPU and CPU water blocks connected to a 360mm radiator. The system achieved peak GPU utilization of 78–82% during training phases, average CPU utilization of 45–60% during preprocessing, memory bandwidth of 89.6 GB/s for GPU operations and 51.2 GB/s for system memory, GPU temperatures maintained at 65–72 °C under full load, and total power consumption ranging from 650-750W during intensive training phases.

The software environment utilized Ubuntu Linux 22.04.3 LTS running kernel version 5.15.0–91-generic as the base operating system. PyTorch version 2.0.1 served as the primary deep learning framework, compiled with CUDA support for GPU acceleration. The NVIDIA CUDA Toolkit version 11.8 with cuDNN 8.7.0 enabled efficient GPU computation throughout all training and inference operations. Python version 3.9.16 from the Anaconda distribution provided the core programming environment. OpenCV version 4.8.0 was employed for image preprocessing tasks, compiled with CUDA support. Scientific computing relied on NumPy version 1.24.3 with MKL-optimized builds and SciPy version 1.10.1 with BLAS/LAPACK integration. Data manipulation utilized Pandas version 2.0.3, visualization used Matplotlib version 3.7.1 and Seaborn version 0.12.2, machine learning evaluation employed Scikit-learn version 1.3.0, and image augmentation used Albumentations version 1.3.1. Utility packages included tqdm version 4.65.0 for progress monitoring, TensorBoard version 2.13.0 for training visualization, and Weights & Biases version 0.15.8 for experiment tracking. The development environment utilized Visual Studio Code version 1.81.0, version control through Git version 2.40.1, and Docker version 24.0.5 for deployment testing. Code quality was maintained using Black version 23.7.0, isort version 5.12.0, Pylint version 2.17.5, and Flake8 version 6.1.0.

The hyperparameter configuration was established through systematic grid search and preliminary validation experiments. For optimization settings, the generator learning rate was set to 2 × 10⁻^4^ (search range [1 × 10⁻^5^, 5 × 10⁻^4^]) and discriminator learning rate to 1 × 10⁻^4^ (search range [5 × 10⁻^5^, 2 × 10⁻^4^]). The Adam optimizer was selected with Beta₁ = 0.5 (search range [0.3, 0.9]) and Beta₂ = 0.999 (search range [0.99, 0.9999]). Weight decay (L2 regularization) was configured at 1 × 10⁻^5^ (search range [0, 1 × 10⁻^4^]) and gradient clipping at threshold 1.0 (search range [0.5, 2.0]). The training schedule comprised 200 total epochs with 20 warmup epochs, metaheuristic integration starting at epoch 21, and fine-tuning phase from epochs 101–200. Early stopping patience was set at 30 epochs, learning rate decay used ReduceLROnPlateau scheduler with factor 0.5 and patience 15 epochs, batch size was 16 images, and gradient accumulation over 2 steps achieved an effective batch size of 32. Loss function weights included α (Chaoborus hunting) = 0.4, β (Chaoborus migration) = 0.3, γ (Chaoborus reproduction) = 0.3, δ (Crayfish foraging) = 0.35, ε (Crayfish social) = 0.35, ζ (Crayfish territorial) = 0.30, λ (identity preservation) = 10.0, and μ (adversarial weight) = 1.0. Network architecture specified generator and discriminator channels as [64, 128, 256, 512, 1024], kernel size 4 × 4, stride 2, padding 1, LeakyReLU activation with negative slope 0.2, Tanh output activation, BatchNorm2d normalization, and dropout rate 0.5 in bottleneck only. Data processing configured input resolution at 256 × 256 pixels, three color channels for RGB thermal representation, normalization range [-1, 1], temperature range 0–100°C, and missing pixel threshold at 0.15 (maximum 15% missing pixels per image). Data augmentation parameters included random rotation ± 15°, random scale 0.8–1.2, horizontal flip probability 0.5, Gaussian noise σ = 0.01, and brightness/contrast adjustments ± 0.1. Convergence criteria specified loss stability threshold ε = 1 × 10⁻^4^ with 5 consecutive stable epochs required, validation frequency every 5 epochs, and checkpoint saving for best validation loss.

**Fig. 1:**
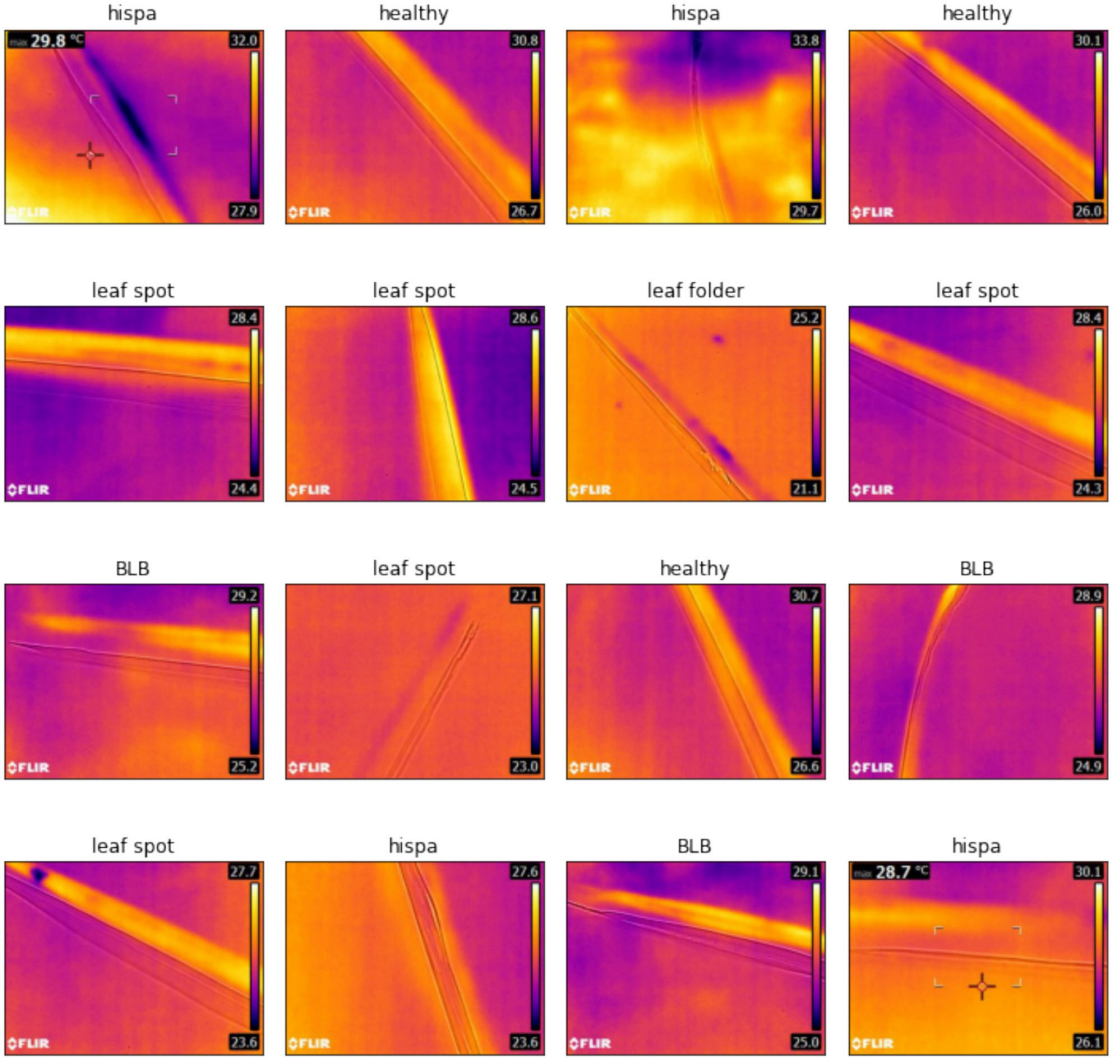
Samples from dataset exploration.

**Fig. 2:**
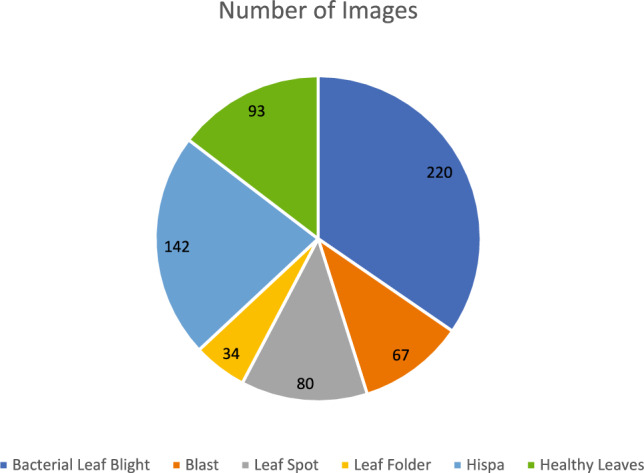
Distribution of the images across the dataset.

Reproducibility measures ensured transparent and replicable experimental procedures. Random seed 42 was fixed for all random number generators across NumPy, PyTorch, and CUDA operations. Deterministic algorithm execution was enforced through torch.use_deterministic_algorithms(True) where supported, and CUDA determinism was guaranteed through torch.backends.cudnn.deterministic = True and torch.backends.cudnn.benchmark = False settings. Complete conda environment specifications were exported to environment.yml files capturing exact dependency versions. All hyperparameters were stored in structured YAML configuration files for programmatic access and version control. Comprehensive experiment tracking maintained full logs of all training runs through Weights & Biases integration, enabling detailed performance analysis and comparison. Code and model availability provisions include source code repository access, trained model weights for all configurations, complete configuration files in supplementary materials, preprocessing scripts with detailed documentation, and evaluation scripts with comprehensive usage instructions, all to be provided upon manuscript acceptance. Figure 1 illustrates representative thermal image samples across all six disease categories and healthy leaves, demonstrating the visual diversity and thermal signature characteristics present in the dataset. The class distribution visualization in Fig. 2 shows the intentional imbalance reflecting natural disease prevalence patterns, with Bacterial Leaf Blight (34.6%) as the most frequent and Leaf Folder (5.3%) as the least frequent category.

#### Image acquisition specifications

The thermal images were captured using the FLIR E8 thermal camera, chosen for its high precision and reliability in agricultural applications. The technical specifications ensure consistent and accurate thermal data acquisition across all experimental conditions. The FLIR E8 features an infrared resolution of 76,800 pixels arranged in a 320 × 240 configuration, providing sufficient detail for disease detection applications. The camera maintains a temperature accuracy of ± 2% for ambient temperatures ranging from 10 °C to 35 °C (50 °F to 95 °F) and object temperatures above 0 °C (32 °F). The field of view spans 45° × 34°, enabling comprehensive leaf coverage while maintaining thermal resolution quality. Additional specifications include thermal sensitivity below 0.06 °C at 30 °C, radiometric JPEG image format, and spectral range coverage from 7.5 to 14 μm for optimal thermal signature capture.

#### Data collection protocol

The thermal image acquisition followed a standardized protocol to ensure data consistency and minimize environmental variability. Images were captured during optimal conditions to maximize thermal signature clarity and diagnostic accuracy. Collection sessions were conducted during morning hours between 8:00 and 10:00 AM to minimize solar interference and maintain consistent lighting conditions. Environmental parameters were carefully controlled with ambient temperatures maintained between 25 and 30 °C and relative humidity between 60 and 70% to ensure optimal thermal imaging conditions. The camera was positioned at a distance of 30–50 cm from the leaf surface to achieve optimal thermal resolution while maintaining image clarity. Image orientation was kept perpendicular to the leaf surface to minimize reflection artifacts that could interfere with thermal signature detection. Camera calibration procedures were performed before each session using reference standards to maintain measurement accuracy throughout the data collection process.

#### Dataset preprocessing and quality assessment

Prior to model training, the thermal images underwent comprehensive preprocessing to ensure data quality and consistency. The preprocessing pipeline addresses common thermal imaging challenges while preserving critical disease-related thermal signatures. Thermal calibration procedures normalized temperature readings based on ambient conditions to ensure consistency across different collection sessions. Noise reduction techniques employed Gaussian filtering to reduce sensor noise while preserving edge information critical for disease boundary detection. Contrast enhancement through adaptive histogram equalization improved thermal contrast to better distinguish between healthy and diseased tissue regions. Artifact removal procedures eliminated reflection and emissivity artifacts that could interfere with accurate temperature measurements. Standardization processes normalized images to standard temperature ranges to ensure consistent analysis across the entire dataset.

### Proposed dual metaheuristic GAN architecture

The proposed framework integrates two complementary bio-inspired metaheuristic algorithms—the Chaoborus algorithm and the Australian Crayfish algorithm—into a generative adversarial network architecture specifically designed for thermal image augmentation in agricultural disease detection. The architecture comprises three main components: a U-Net-based generator with strategic identity block placement, an enhanced PatchGAN discriminator with multi-scale feature extraction, and dual metaheuristic loss functions that guide optimization through biologically-inspired mechanisms. The generator employs a symmetric encoder-decoder structure with skip connections at multiple resolutions to preserve spatial information. The encoder contains five convolutional blocks, each with four-by-four convolutional layers with stride two, batch normalization, and LeakyReLU activation with negative slope 0.2. Identity blocks are strategically positioned at the 64 × 64 and 32 × 32 resolution encoder-decoder junctions where thermal gradients are most vulnerable to adversarial modification, providing direct skip pathways that bypass potentially destructive transformations. The decoder mirrors the encoder with five transposed convolutional blocks using four-by-four kernels with stride two, batch normalization, ReLU activation, and concatenation with encoder features via skip connections. The final generator layer uses a one-by-one convolutional layer with Tanh activation producing normalized output images. The discriminator employs a PatchGAN architecture classifying 70 × 70 overlapping patches as real or fake, enabling effective local thermal pattern discrimination. The discriminator consists of five convolutional layers with progressively increasing channels from 64 to 512, using four-by-four kernels with stride two, batch normalization except first and last layers, and LeakyReLU activation with negative slope 0.2.

The Chaoborus algorithm, inspired by phantom midge larvae hunting, migration, and reproductive behaviors, optimizes generator performance through three complementary loss components. The hunting phase loss, weighted at 0.4, implements aggressive pixel-wise search computing L1 distance between generated and real thermal images, ensuring accurate missing pixel imputation and absolute temperature accuracy critical for detecting 1–3 °C variations distinguishing healthy from diseased tissue. The migration phase loss, weighted at 0.3, optimizes thermal gradient preservation computing L2 distance between image gradients, ensuring smooth temperature transitions within homogeneous tissue while maintaining sharp boundaries at disease margins, preserving thermal signatures that distinguish pathological conditions with 2–5° elevations at infection sites. The reproduction phase loss, weighted at 0.3, focuses on structural similarity using SSIM to ensure generated images maintain coherence with real thermal distributions, preventing unrealistic patterns. The Chaoborus generator loss combines these three phases with weights emphasizing pixel accuracy while balancing gradient preservation and structural coherence based on systematic grid search optimization.

The Australian Crayfish algorithm, inspired by freshwater crayfish foraging, social interaction, and territorial defense behaviors, optimizes discriminator performance through three complementary loss components enforcing spatial relationships and preserving thermal boundaries. The foraging phase loss, weighted at 0.35, implements adaptive connectivity optimization strengthening connections within homogeneous thermal regions while weakening connections across sharp boundaries, enabling discrimination of realistic spatial patterns by enforcing eight-pixel neighborhood relationships respecting thermodynamic heat diffusion. The social phase loss, weighted at 0.35, enhances inter-pixel relationships promoting correlated thermal patterns ensuring neighboring pixels exhibit thermally plausible relationships, rejecting images with unrealistic discontinuities or isolated temperature spikes. The territorial phase loss, weighted at 0.30, preserves spatial boundaries maintaining disease-specific thermal boundaries with physiologically realistic temperature gradients while preventing artificial smoothing, using a minimum temperature difference margin of 1.5 °C based on disease-induced elevation. The complete training objective combines both metaheuristic algorithms with identity block preservation, where identity loss weighted at 10.0 enforces generator preservation of input images when given real thermal images, preventing artifact introduction. Training proceeds through alternating optimization with discriminator updates using Crayfish loss on batches of real and generated images, followed by generator updates using Chaoborus loss, with identity loss computed on real images to enforce feature preservation. This dual approach addresses multi-objective optimization where Chaoborus ensures pixel-level accuracy and gradient preservation for absolute temperature measurements while Crayfish enforces spatial coherence and boundary preservation for disease pattern recognition, with identity blocks providing architectural support for thermal signature preservation. Algorithm 1 shows the Dual Metaheuristic GAN for Thermal Image Augmentation.

**Algorithm 1:**
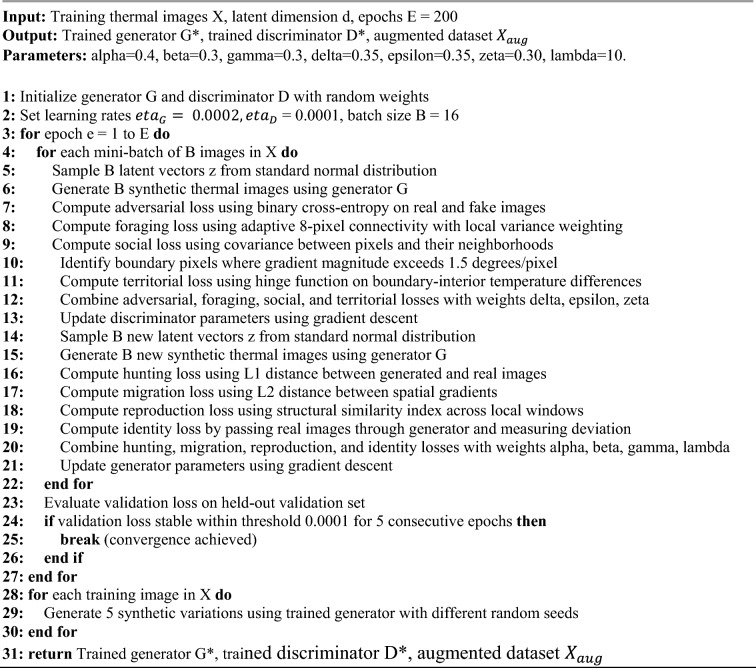
Dual metaheuristic GAN for thermal image augmentation.

#### Overall architecture design

**Fig. 3:**
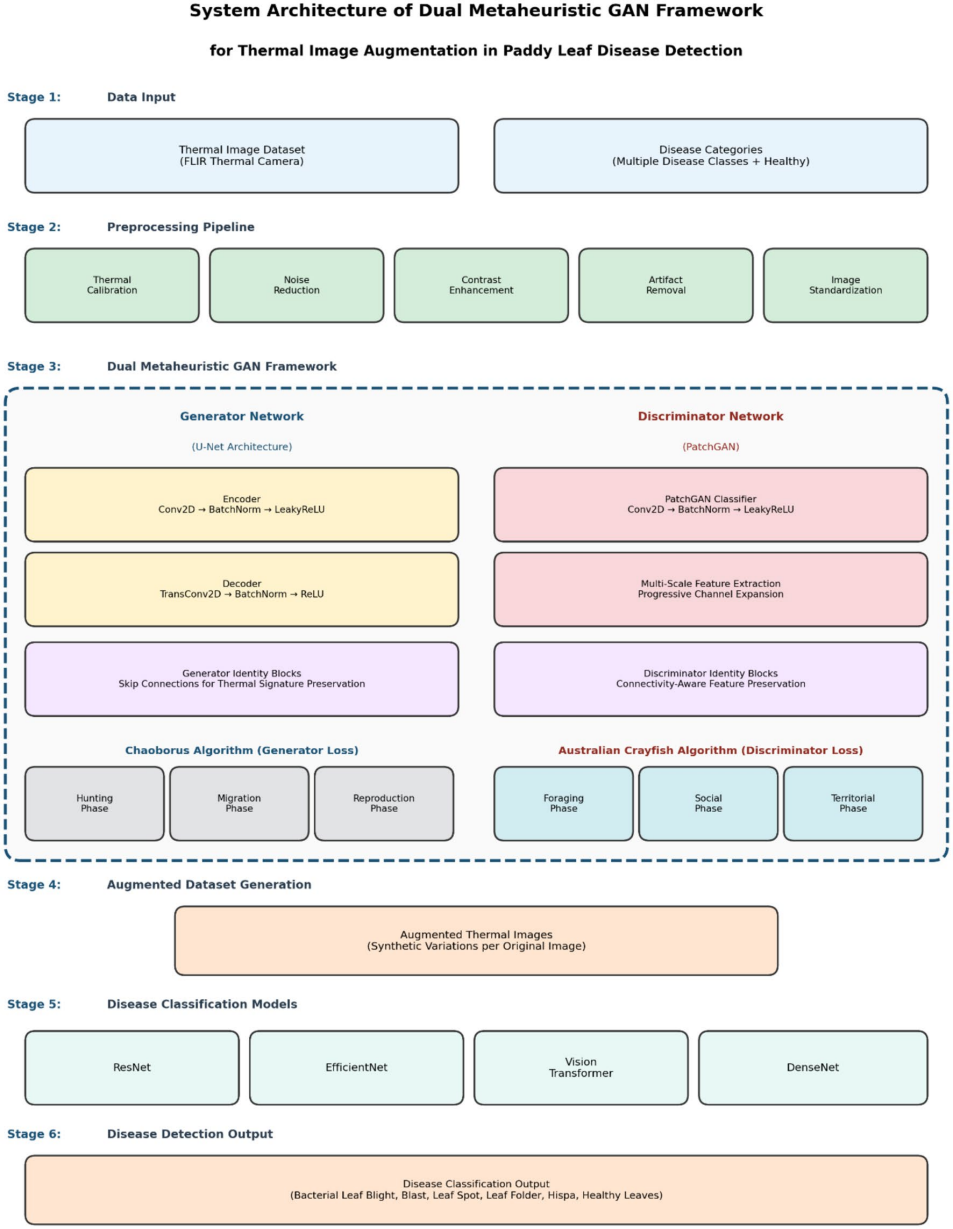
Phase diagram of dual metaheuristic GAN framework methodology for thermal image augmentation in paddy leaf disease detection.

The proposed architecture extends the traditional GAN framework through three key modifications. First, dual metaheuristic loss functions replace conventional adversarial losses to provide more intelligent optimization strategies. Second, identity blocks are strategically positioned to preserve thermal signatures throughout the training process. Third, adaptive connectivity mechanisms enhance feature extraction capabilities for improved disease pattern recognition. The framework consists of a modified U-Net generator with identity blocks, an enhanced PatchGAN discriminator with adaptive connectivity, a Chaoborus loss module for bio-inspired generator optimization, an Australian Crayfish loss module for connectivity-aware discriminator optimization, and an identity preservation layer for thermal signature conservation. Figure 3 presents the complete methodological framework showing the three sequential stages: thermal image preprocessing and feature extraction, dual metaheuristic GAN-based augmentation with Chaoborus and Australian Crayfish algorithms, and disease classification using deep learning architectures.

#### Chaoborus algorithm integration

The Chaoborus algorithm, inspired by phantom midge larvae hunting behavior, serves as the novel generator loss function for intelligent missing pixel imputation. The algorithm mimics the predatory strategies of Chaoborus species, which exhibit sophisticated prey detection and capture mechanisms in aquatic environments.

The Chaoborus loss function is defined as Eq. (1).

1 $${L}_{\mathrm{chaoborus}\left(G\right)}= \alpha \cdot {L}_{\mathrm{hunt}\left(G\right)}+ \beta \cdot {L}_{\mathrm{migration}\left(G\right)}+ \gamma \cdot {L}_{\mathrm{reproduction}\left(G\right)}$$

where ${L}_{hunt\left(G\right)}$ represents hunting phase optimization for missing pixel identification, ${L}_{migration\left(G\right)}$ denotes migration phase for optimal pixel value prediction, ${L}_{reproduction\left(G\right)}$ indicates reproduction phase for thermal signature preservation, and α, β, γ are adaptive weighting parameters with values α = 0.4, β = 0.3, γ = 0.3.

The hunting phase optimization is formulated as Eq. (2).

2 $${L}_{\mathrm{hunt}\left(G\right)}= {\sum }_{i}^{=1\mathrm{H}}{\sum }_{j}^{=1\mathrm{W}}{\left|\left|M\left(i,j\right)\cdot \left({I}_{\mathrm{real}\left(i,j\right)}- G\left(z\right)\left(i,j\right)\right)\right|\right|}^{22}$$

The migration phase optimization is expressed as Eq. (3).

3 $${L}_{\mathrm{migration}\left(G\right)}={\sum }_{i}^{=1\mathrm{H}}{\sum }_{j}^{=1\mathrm{W}}{\left|\left|\nabla T\left(i,j\right)\cdot \left({I}_{\mathrm{real}\left(i,j\right)}- G\left(z\right)\left(i,j\right)\right)\right|\right|}^{1}$$

The reproduction phase optimization is defined as Eq. (4).

4 $${L}_{\mathrm{reproduction}\left(G\right)}= {\left|\left|SSIM\left({I}_{\mathrm{real}}, G\left(z\right)\right)- 1\right|\right|}^{22}$$

**Algorithm 2:**
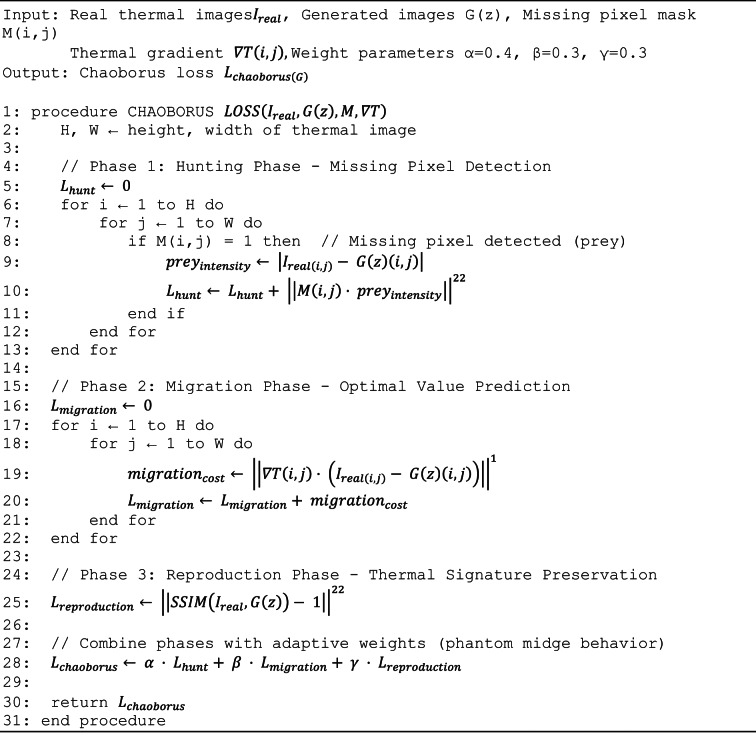
Chaoborus-inspired generator loss optimization.

#### Australian Crayfish algorithm integration

The Australian Crayfish algorithm optimizes adaptive 8-pixel connectivity in the discriminator network, inspired by crayfish foraging and territorial behaviors. The algorithm addresses spatial relationships in thermal images while maintaining disease-specific pattern recognition.

The Australian Crayfish loss function is expressed as Eq. (5).

5 $${L}_{\mathrm{crayfish}\left(D\right)}= \delta \cdot {L}_{\mathrm{foraging}\left(D\right)}+ \varepsilon \cdot {L}_{\mathrm{social}\left(D\right)}+ \zeta \cdot {L}_{\mathrm{territorial}\left(D\right)}$$

where ${L}_{foraging\left(D\right)}$ represents adaptive 8-pixel connectivity optimization, ${L}_{social\left(D\right)}$ denotes inter-pixel relationship enhancement, ${L}_{territorial\left(D\right)}$ indicates spatial boundary preservation for disease regions, and δ, ε, ζ are dynamic weighting parameters based on thermal gradient intensity.

The foraging behavior optimization is formulated as Eq. (6).

6 $${L}_{foraging\left(D\right)}={\sum }_{\mathrm{k}}^{=18}{\left|\left|{W}_{conn\left(k\right)}\cdot \left(D\left({x}_{real}\right)- D\left(G\left(z\right)\right)\right)\right|\right|}^{22}$$

The social interaction optimization is expressed as Eq. (7).

7 $${L}_{social\left(D\right)}={\sum }_{\mathrm{i}}^{=1\mathrm{H}}{\sum }_{\mathrm{j}}^{=1\mathrm{H}}{\left|\left|C\left(i,j\right)\cdot \left(\nabla D\left({x}_{real}\right)\left(i,j\right)- \nabla D\left(G\left(z\right)\right)\left(i,j\right)\right)\right|\right|}^{1}$$

The territorial defense optimization is defined as Eq. (8).

8 $${L}_{territorial\left(D\right)}= {\left|\left|{\nabla }^{2}{T}_{boundary}\cdot \left(D\left({x}_{real}\right)- D\left(G\left(z\right)\right)\right)\right|\right|}^{22}$$

**Algorithm 3:**
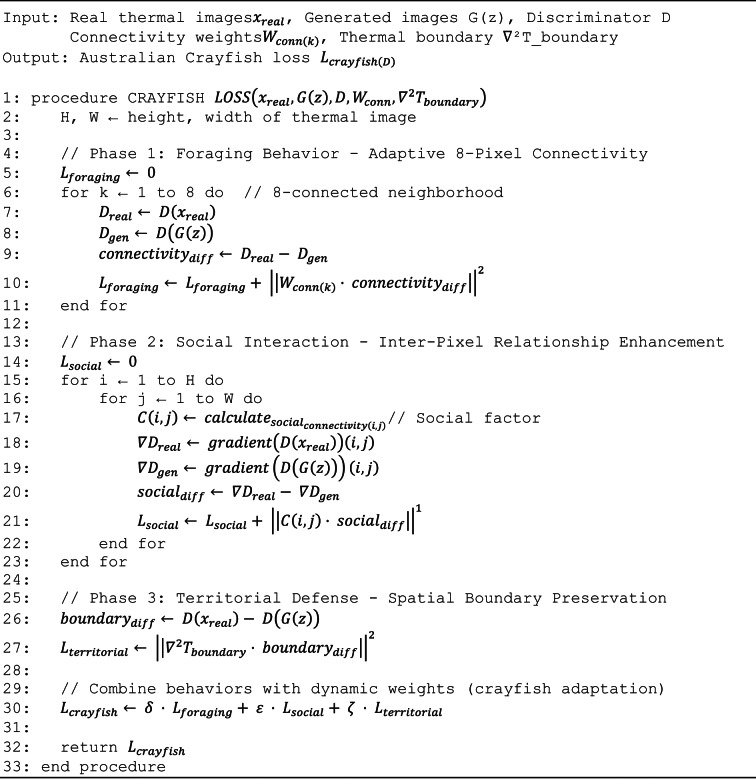
Australian Crayfish-inspired discriminator loss optimization.

#### Combined loss function

The total loss function integrates both metaheuristic algorithms with identity preservation as Eq. (9).

9 $${L}_{\mathrm{total}}= {L}_{\mathrm{chaoborus}\left(G\right)}+ {L}_{\mathrm{crayfish}\left(D\right)}+ {\lambda }_{\mathrm{identity}}\cdot {L}_{\mathrm{identity}}+ \mu \cdot {L}_{\mathrm{adversarial}}$$

where ${L}_{identity}$ represents identity block preservation loss, ${L}_{\mathrm{adversarial}}$ denotes traditional adversarial loss component, ${\lambda }_{\mathrm{identity}}= 10$ is the identity preservation weight, and μ = 1 is the adversarial loss weight.

#### Identity block architecture

Identity blocks are strategically integrated within both generator and discriminator networks to preserve critical thermal signatures during adversarial training. The blocks employ residual connections with thermal-aware normalization.

The Generator Identity Block is defined as Eq. (10).

10 $$GIB\left(x\right)= x + {F}_{\mathrm{thermal}\left(x, {W}_{\mathrm{chaoborus}}\right)}$$

The Discriminator Identity Block is expressed as Eq. (11).

11 $$DIB\left(x\right)= x + {F}_{\mathrm{connectivity}\left(x, {W}_{\mathrm{crayfish}}\right)}$$

#### Network architecture details

Detailed architecture specifications for both generator and discriminator networks are presented in Table 7, highlighting the integration of identity blocks and metaheuristic optimization components.

**Table 7:** Dual metaheuristic GAN architecture specifications.

Component	Layer type	Input size	Output size	Activation	Parameters	Identity block
Generator						
Encoder Block 1	Conv2D + BatchNorm	256 × 256 × 3	128 × 128 × 64	LeakyReLU	12,352	GIB-1
Encoder Block 2	Conv2D + BatchNorm	128 × 128 × 64	64 × 64 × 128	LeakyReLU	73,856	GIB-2
Encoder Block 3	Conv2D + BatchNorm	64 × 64 × 128	32 × 32 × 256	LeakyReLU	295,168	GIB-3
Encoder Block 4	Conv2D + BatchNorm	32 × 32 × 256	16 × 16 × 512	LeakyReLU	1,180,160	GIB-4
Bottleneck	Conv2D + BatchNorm	16 × 16 × 512	8 × 8 × 1024	LeakyReLU	4,719,616	-
Decoder Block 1	ConvTranspose2D + BatchNorm	8 × 8 × 1024	16 × 16 × 512	ReLU	4,719,104	GIB-5
Decoder Block 2	ConvTranspose2D + BatchNorm	16 × 16 × 1024	32 × 32 × 256	ReLU	2,359,808	GIB-6
Decoder Block 3	ConvTranspose2D + BatchNorm	32 × 32 × 512	64 × 64 × 128	ReLU	589,952	GIB-7
Decoder Block 4	ConvTranspose2D + BatchNorm	64 × 64 × 256	128 × 128 × 64	ReLU	147,520	GIB-8
Output layer	ConvTranspose2D	128 × 128 × 128	256 × 256 × 3	Tanh	6,147	–
Discriminator						
Input block	Conv2D + LeakyReLU	256 × 256 × 3	128 × 128 × 64	LeakyReLU	12,352	DIB-1
Block 1	Conv2D + BatchNorm	128 × 128 × 64	64 × 64 × 128	LeakyReLU	73,856	DIB-2
Block 2	Conv2D + BatchNorm	64 × 64 × 128	32 × 32 × 256	LeakyReLU	295,168	DIB-3
Block 3	Conv2D + BatchNorm	32 × 32 × 256	16 × 16 × 512	LeakyReLU	1,180,160	DIB-4
Block 4	Conv2D + BatchNorm	16 × 16 × 512	8 × 8 × 1024	LeakyReLU	4,719,616	DIB-5
Output block	Conv2D	8 × 8 × 1024	4 × 4 × 1	Sigmoid	16,385	–

### Training methodology and hyperparameters

The training process employs a carefully designed protocol that balances the dual metaheuristic objectives while ensuring stable convergence and high-quality image generation. Table 8 details the comprehensive hyperparameter configuration used throughout the training process, including optimization settings, loss weights, and data processing parameters.

**Table 8:** Training hyperparameters and configuration.

Category	Parameter	Value	Description
Optimization	Learning rate (Generator)	2 × 10⁻^4^	Adam optimizer learning rate
	Learning rate (Discriminator)	1 × 10⁻^4^	Slower discriminator training
	Beta 1 (Adam)	0.5	Momentum parameter
	Beta 2 (Adam)	0.999	RMSprop parameter
	Weight decay	1 × 10⁻^5^	L2 regularization
Training schedule	Batch size	16	Optimized for thermal processing
	Total epochs	200	With early stopping
	Warmup epochs	20	Initial adversarial training
	Metaheuristic integration	21–100	Gradual bio-inspired loss introduction
	Fine-tuning phase	101–200	Dual metaheuristic optimization
Loss weights	α (Hunting)	0.4	Chaoborus hunting phase weight
	β (Migration)	0.3	Chaoborus migration phase weight
	γ (Reproduction)	0.3	Chaoborus reproduction phase weight
	δ (Foraging)	0.35	Crayfish foraging weight
	ε (Social)	0.35	Crayfish social interaction weight
	ζ (Territorial)	0.30	Crayfish territorial defense weight
	${\lambda }_{\mathrm{identity}}$	10.0	Identity preservation weight
	${\mu }_{\mathrm{adversarial}}$	1.0	Traditional adversarial weight
Data processing	Input resolution	256 × 256	Thermal image size
	Color channels	3	RGB thermal representation
	Normalization range	[-1, 1]	Tanh activation compatible
	Temperature range	0–100°C	Thermal calibration range
Augmentation	Rotation range	± 15°	Thermal-preserving rotation
	Scale factor	0.8–1.2	Zoom variation
	Gaussian noise σ	0.01	Sensor noise simulation
	Horizontal flip	50% probability	Random horizontal flipping

#### Convergence criteria

Training convergence is monitored using multiple criteria to ensure stable and effective learning. The convergence condition is evaluated based on the stability of the total loss function over consecutive epochs.

12 $$\mathrm{Convergence} = \{ \mathrm{True},\text{ if} \Delta L\_\mathrm{total} < \varepsilon \text{ for }n\text{ consecutive epochs }\{\text{ False},\text{ otherwise}$$

#### Model flow and processing pipeline

The complete processing pipeline comprises three sequential stages: thermal image preprocessing and feature extraction, dual metaheuristic GAN-based augmentation with optimization, and disease classification using deep learning architectures. The workflow begins with raw thermal images from FLIR E8 camera undergoing systematic preprocessing. Thermal calibration normalizes temperature measurements based on ambient conditions including atmospheric temperature, humidity, and distance to target. Adaptive Gaussian filtering with kernel size five-by-five and sigma 0.8 reduces sensor noise while preserving edges, followed by adaptive histogram equalization with clip limit 2.0 applied to eight-by-eight pixel tiles enhancing thermal contrast. Artifact removal algorithms detect and correct reflection artifacts and emissivity variations using gradient-based detection. Images are standardized to 0–100 °C ranges through min–max normalization, then resized to 256 × 256 pixels using bicubic interpolation. Preprocessed images undergo feature extraction through the encoder portion of the U-Net generator, where five successive convolutional blocks progressively extract hierarchical features from low-level edges and textures through mid-level thermal patterns to high-level semantic disease features. Each encoding step halves spatial dimensions while doubling channels, creating representations at 256 × 256 with 64 channels, 128 × 128 with 128 channels, 64 × 64 with 256 channels, 32 × 32 with 512 channels, and 16 × 16 with 1024 channels at bottleneck. These multi-scale representations capture thermal characteristics at different spatial scales, enabling simultaneous processing of fine-grained local temperature variations indicating early disease and coarse-grained global patterns indicating advanced infections.

The optimization stage employs dual metaheuristic algorithms operating in parallel to guide generator and discriminator learning. During each training iteration, the generator receives either random latent vectors for generating synthetic images or real images with artificially introduced missing pixels for imputation, processing inputs through encoder to extract features then through decoder to reconstruct enhanced images. The Chaoborus algorithm evaluates generator outputs through three parallel assessments: hunting phase computes pixel-wise differences assessing reconstruction accuracy, migration phase evaluates spatial gradient preservation comparing temperature rate-of-change patterns, and reproduction phase calculates structural similarity ensuring global coherence. These assessments combine with weights 0.4, 0.3, and 0.3 producing total generator loss that backpropagates to update parameters. Simultaneously, the discriminator receives batches containing equal numbers of real and synthetic images, processing each through five convolutional layers producing spatial probability maps indicating real versus fake classification for 70 × 70 patches. The Australian Crayfish algorithm evaluates discriminator performance through three mechanisms: foraging phase computes adaptive connectivity scores within eight-pixel neighborhoods assessing spatial coherence, social phase evaluates inter-pixel correlation patterns verifying thermally plausible relationships, and territorial phase measures boundary preservation at disease margins ensuring realistic temperature gradients. These assessments combine with weights 0.35, 0.35, and 0.30 alongside adversarial loss producing total discriminator loss. Identity preservation operates by periodically passing real images through generator and computing reconstruction differences, with loss weighted at 10.0 enforcing preservation of correct thermal signatures. This alternating optimization continues for two-hundred epochs with discriminator updating on each batch followed by generator updates, gradually improving both networks until convergence when validation loss stabilizes for five consecutive epochs within threshold 0.0001.

The classification stage utilizes the optimized generator to augment the limited training dataset, creating synthetic thermal images expanding effective training size while maintaining diagnostic integrity. For each original training image, the generator produces five synthetic variations by introducing different random missing pixel patterns then imputing them, expanding the 445-image training set to 2670 images including originals and augmentations. Augmented images undergo the same preprocessing ensuring consistent formatting before feeding into disease classifiers. Four pre-trained architectures serve as classifiers: Vision Transformer pre-trained on ImageNet then fine-tuned on thermal images, ResNet-50 with transfer learning, EfficientNet-B7 providing compound scaling, and DenseNet-201 with dense connectivity. Each classifier receives augmented training data with class weights inversely proportional to disease frequencies addressing imbalance, using cross-entropy loss for multi-class classification across six categories including five diseases and healthy leaves. Training proceeds for one-hundred epochs with early stopping monitoring validation accuracy, employing Adam optimizer with learning rate 0.0001, batch size 32, and learning rate reduction by factor 0.5 when validation accuracy plateaus for ten epochs. During inference, test images bypass augmentation and proceed directly through preprocessing to classifier networks outputting probability distributions across six categories through softmax activation. The predicted class corresponds to maximum probability category, while prediction confidence derives from the probability value. The complete pipeline from raw thermal acquisition through preprocessing, feature extraction, augmentation via dual metaheuristic optimization, and final disease classification enables robust automated disease detection suitable for agricultural field deployment.

### Mathematical formulations of dual metaheuristic algorithms

#### Chaoborus algorithm: generator optimization

The Chaoborus algorithm optimizes the generator network through three biologically-inspired phases that collectively address pixel-level reconstruction, thermal gradient preservation, and structural similarity. Each phase contributes a distinct loss component that guides the generator toward producing high-quality thermal images with preserved disease-specific signatures.

##### Hunting phase (Missing pixel imputation)

The hunting phase implements aggressive pixel-wise search for optimal intensity values, computing the L1 reconstruction loss between generated and real thermal images. This phase emphasizes pixel-level accuracy with weight α = 0.4, ensuring generated images maintain absolute temperature measurements critical for disease detection where variations of 1–3°C distinguish healthy from diseased tissue.

13 $${L}_{\mathrm{hunting}}=\frac{1}{N}\sum_{i=1}^{N}\mid G(z{)}_{i}-{x}_{i}\mid$$

where G(z) denotes the generated thermal image from latent vector z ∈ ℝ^d, x represents the real thermal image from the training dataset, N = H × W is the total number of pixels (for 256 × 256 images, N = 65,536), i indexes individual pixels across the spatial dimensions, and |·| represents the absolute value (L1 norm).

##### Migration phase (Thermal gradient preservation)

The migration phase optimizes thermal gradient preservation through spatial derivative comparison, computing the L2 distance between image gradients. The spatial gradient operator ∇ captures temperature rate-of-change across leaf surfaces, with horizontal gradient ∂I/∂x ≈ I(i + 1,j)—I(i,j) and vertical gradient ∂I/∂y ≈ I(i,j + 1)—I(i,j) approximated using forward differences. This phase emphasizes thermal gradient preservation with weight β = 0.3, ensuring smooth temperature transitions within homogeneous regions while maintaining sharp boundaries at disease margins where temperature elevations typically range from 2–5°C at infection sites.

14 $${L}_{\mathrm{migration}}=\frac{1}{N}\sum_{i=1}^{N}\parallel \nabla G(z{)}_{i}-\nabla {x}_{i}{\parallel }_{2 }^{2}$$

where ∇I(i,j) = [∂I/∂x, ∂I/∂y]^T represents the spatial gradient vector and ||·||₂^2^ denotes the squared L2 norm computed as ||v||₂^2^ = v_x^2^ + v_y^2^.

The reproduction phase focuses on structural similarity preservation using the multi-scale Structural Similarity Index (SSIM), which evaluates luminance, contrast, and structure comparisons across local windows. SSIM is computed across all overlapping 11 × 11 windows and averaged to produce the final structural similarity score. This phase emphasizes structural coherence with weight γ = 0.3, ensuring generated images maintain overall thermal distribution patterns consistent with real thermal signatures.

15 $${L}_{\mathrm{reproduction}}=1-{\mathrm{SSIM}}\left(G\left(z\right),x\right)$$

where SSIM is computed as:

16 $${\mathrm{SSIM}}(G(z),x)=[l(G(z),x){]}^{{\alpha }_{s}}\cdot [c(G(z),x){]}^{{\beta }_{s}}\cdot [s\left(G\left(z\right),x\right){]}^{{\gamma }_{s}}$$

with component functions for luminance comparison:

17 $$l\left(G\left(z\right),x\right)=\frac{2{\mu }_{G\left(z\right)}{\mu }_{x}+{C}_{1}}{{\mu }_{G\left(z\right)}^{2}+{\mu }_{x}^{2}+{C}_{1}}$$

contrast comparison:

18 $$c\left(G\left(z\right),x\right)=\frac{2{\sigma }_{G\left(z\right)}{\sigma }_{x}+{C}_{2}}{{\sigma }_{G\left(z\right)}^{2}+{\sigma }_{x}^{2}+{C}_{2}}$$

and structure comparison:

19 $$s\left(G\left(z\right),x\right)=\frac{{\sigma }_{G\left(z\right),x}+{C}_{3}}{{\sigma }_{G\left(z\right)}{\sigma }_{x}+{C}_{3}}$$

The complete Chaoborus algorithm combines the three phases with learned weights that were determined through systematic grid search over the ranges α ∈ [0.2, 0.6], β ∈ [0.2, 0.4], γ ∈ [0.2, 0.4] with step size 0.05, subject to constraint α + β + γ = 1.0. The optimal configuration (α = 0.4, β = 0.3, γ = 0.3) was selected based on validation set PSNR and thermal signature preservation metrics.

20 $${L}_{\mathrm{Chaoborus}\_G}=\alpha \cdot {L}_{\mathrm{hunting}}+\beta \cdot {L}_{\mathrm{migration}}+\gamma \cdot {L }_{\mathrm{reproduction}}$$

where α = 0.4 emphasizes pixel-level accuracy, β = 0.3 balances gradient preservation, and γ = 0.3 balances structural coherence, with normalized weights ensuring balanced contribution across all optimization objectives.

#### Australian Crayfish algorithm: discriminator optimization

The Australian Crayfish algorithm optimizes the discriminator network through three biologically-inspired phases that enforce spatial relationships, enhance inter-pixel dependencies, and preserve thermal boundaries essential for disease classification. Each phase targets distinct aspects of spatial thermal pattern discrimination.

The foraging phase implements adaptive connectivity optimization based on local thermal characteristics, strengthening connectivity within homogeneous thermal regions such as healthy tissue or uniform disease manifestations while weakening connectivity across sharp thermal boundaries at disease margins. The adaptive weight w(i,j) is high (approaching 1) in homogeneous regions with low local variance and low (approaching 0) in heterogeneous regions with high variance, enabling the discriminator to distinguish realistic smooth thermal patterns from artificially generated discontinuities. This phase emphasizes spatial coherence with weight δ = 0.35.

21 $${L}_{\mathrm{foraging}}=\frac{1}{M}\sum_{i=1}^{N}\sum_{j\in {\mathcal{N}}_{8}(i)}w(i,j)\cdot \mid I(i)-I(j)\mid$$

where i indexes all pixels in the image, N₈(i) = {(i ± 1, j), (i, j ± 1), (i ± 1, j ± 1)} represents the 8-connected neighborhood, j ∈ N₈(i) iterates over the 8 neighboring pixels, I(i) denotes the intensity (temperature) at pixel i, M = ∑*i ∑*{j ∈ N₈(i)} 1 is the total number of neighbor pairs (M ≈ 8N for interior pixels), and w(i,j) is the adaptive connectivity weight defined as:

22 $$w\left(i,j\right)=\mathrm{exp}\frac{{\sigma }_{\mathrm{local}}^{2}\left(i,j\right)}{2{\tau }^{2}}$$

The social phase enhances inter-pixel relationships through cooperative mechanisms that promote correlated thermal patterns within connected regions, ensuring neighboring pixels exhibit thermally plausible relationships reflecting heat diffusion physics. The phase computes the normalized correlation between each pixel and its neighborhood, with high correlation indicating thermally plausible heat diffusion patterns and low correlation indicating unrealistic isolated temperature spikes. The negative sign converts correlation maximization into a minimization loss suitable for gradient descent optimization. This phase emphasizes inter-pixel relationships with weight ε = 0.35.

23 $${L}_{social}=-\frac{1}{N}\sum_{i=1}^{N}\frac{{\mathrm{Cov}}(I(i),I({\mathcal{N}}_{8}(i)))}{\sigma (i)\cdot \sigma ({\mathcal{N}}_{8}(i))+\varepsilon }$$

where the covariance is computed as:

24 $${\mathrm{Cov}}(I(i),I({\mathcal{N}}_{8}(i)))=\frac{1}{\mid {\mathcal{N}}_{8}\left(i\right)\mid }\sum_{j\in {\mathcal{N}}_{8}\left(i\right)}(I(i)-{\mu }_{i})(I(j)-{\mu }_{j})$$

The territorial phase preserves spatial boundaries through defensive mechanisms that maintain disease-specific thermal boundaries with physiologically realistic temperature gradients while preventing artificial smoothing that could obscure diagnostic features. The phase identifies boundary pixels through gradient magnitude thresholding, then enforces minimum temperature differences between boundaries and interior regions using a hinge loss that penalizes only boundaries with insufficient temperature contrast. The minimum temperature difference margin Δ_min = 1.5°C is based on minimum disease-induced temperature elevation observed in preliminary pathology studies, consistent with physiologically realistic elevations of 2–5 °C at infection sites. This phase emphasizes boundary preservation with weight ζ = 0.30.

25 $${L}_{\mathrm{territorial}}=\frac{1}{B}\sum_{i\in {\mathrm{Boundary}}}\mathrm{max}(0,{\Delta }_{\mathrm{min}}-\mid I(i)-I({\mathrm{interior}}(i))\mid )$$

where Boundary = {i : ||∇I(i)||₂ > gradient} is the set of boundary pixels identified by gradient magnitude threshold gradient = 1.5°C/pixel, ||∇I(i)||₂ = √[(∂I/∂x)^2^ + (∂I/∂y)^2^] is the gradient magnitude, B =|Boundary| is the total number of boundary pixels, interior(i) is the nearest interior pixel to boundary pixel i defined as:

26 $${\mathrm{interior}}\left(i\right)=\mathrm{arg}\underset{j\in {\mathrm{Interior}}\left(i\right)}{\mathrm{min}\parallel i-j{\parallel }_{2}}$$

where Interior(i) = {j : ||∇I(j)||₂ ≤ gradient and ||i—j||₂ ≤ search} with search radius search = 5 pixels, Δ min = 1.5°C is the minimum temperature difference margin, max(0, ·) is the hinge loss function, and |I(i)—I(interior(i))| is the actual temperature difference between boundary and interior pixels.

The complete Crayfish algorithm combines the three biologically-inspired phases with the standard adversarial discriminator loss. The adversarial component encourages the discriminator to output D(x) → 1 for real images and D(G(z)) → 0 for generated images through binary cross-entropy loss. The weights were determined through systematic grid search over ranges δ ∈ [0.2, 0.5], ε ∈ [0.2, 0.5], ζ ∈ [0.2, 0.4] with step size 0.05, with optimal configuration (δ = 0.35, ε = 0.35, ζ = 0.30) selected based on validation set FID score and disease classification accuracy.

27 $${L}_{\mathrm{Crayfish}\_D}={L}_{\mathrm{adversarial}}+\delta \cdot {L}_{\mathrm{foraging}}+\varepsilon \cdot {L}_{\mathrm{social}}+\zeta \cdot {L}_{\mathrm{territorial}}$$

where the standard adversarial discriminator loss is:

28 $${L}_{\mathrm{adversarial}}=-\frac{1}{2N}\left[\sum_{i=1}^{N}\mathrm{log}D\left({x}_{i}\right)+\sum_{i=1}^{N}\mathrm{log}\left(1-D\left(G\left({z}_{i}\right)\right)\right)\right]$$

with D(x) as the discriminator output probability for real image x, D(G(z)) as the discriminator output probability for generated image G(z), and log(·) as the natural logarithm. The weights are δ = 0.35 emphasizing spatial coherence, ε = 0.35 emphasizing inter-pixel correlation, and ζ = 0.30 balancing boundary preservation.

#### Identity preservation loss

The identity preservation mechanism enforces that the generator preserves input images when given real thermal images rather than latent noise, preventing the generator from introducing artifacts into already-correct thermal signatures during adversarial training. This mechanism is implemented by periodically passing real thermal images through the generator and computing the L1 reconstruction difference, penalizing any modification of correct thermal patterns.

29 $${L}_{\mathrm{identity}}=\frac{1}{N}\sum_{i=1}^{N}\mid G({x}_{i})-{x}_{i}\mid$$

where G(x) is the generator output when real image x is provided as input, x is the real thermal image, and the loss penalizes any deviation from the identity mapping. The identity loss is weighted at λ = 10.0 and combined with the generator loss during training to provide strong regularization preserving diagnostic thermal signatures.

#### Complete training objective and optimization

The complete framework training objective combines all loss components from both metaheuristic algorithms with identity preservation, creating a multi-objective optimization problem that simultaneously addresses pixel-level accuracy, gradient preservation, structural similarity, spatial coherence, inter-pixel correlation, boundary preservation, adversarial discrimination, and feature preservation.

30 $${L}_{\mathrm{total}}={L}_{\mathrm{Chaoborus}\_G}+{L}_{\mathrm{Crayfish}\_D}+\lambda \cdot {L}_{\mathrm{identity}}$$

with parameters α = 0.4, β = 0.3, γ = 0.3, δ = 0.35, ε = 0.35, ζ = 0.30, and λ = 10.0 as previously defined. Training proceeds through alternating gradient descent optimization where the discriminator parameters are updated using gradients from the Crayfish loss components and adversarial loss, followed by generator parameter updates using gradients from the Chaoborus loss components and identity loss.

Discriminator Update:

31 $${\theta }_{D}\leftarrow {\theta }_{D}-{\eta }_{D}{\nabla }_{{\theta }_{D}}\left({L}_{\mathrm{adversarial}}+\delta \cdot {L}_{\mathrm{foraging}}+\varepsilon \cdot {L}_{\mathrm{social}}+\zeta \cdot {L}_{\mathrm{territorial}}\right)$$

Generator Update:

32 $${\theta }_{G}\leftarrow {\theta }_{G}-{\eta }_{G}{\nabla }_{{\theta }_{G}}\left(\alpha \cdot {L}_{\mathrm{hunting}}+\beta \cdot {L}_{\mathrm{migration}}+\gamma \cdot {L}_{\mathrm{reproduction}}+\lambda \cdot {L}_{\mathrm{identity}}\right)$$

where ${\theta }_{D}$ represents discriminator parameters, ${\theta }_{G}$ represents generator parameters, ${\nabla }_{\theta }$ denotes the gradient operator with respect to parameters $\theta , {\eta }_{D}= 1\times {10}^{-4}$ is the discriminator learning rate, and ${\eta }_{G}= 2\times {10}^{-4}$ is the generator learning rate. The optimization continues for 200 epochs with convergence achieved when validation loss stabilizes within threshold ε = 1 × 10⁻^4^ for 5 consecutive epochs, indicating that both networks have reached equilibrium in the adversarial training process.

### Implementation framework and architecture visualization

Table 9 provides comprehensive implementation details including software frameworks, hardware specifications, and computational requirements for reproducible research.

**Table 9:** Implementation framework and computational requirements.

Component	Specification	Details	Justification
Hardware platform	GPU	NVIDIA RTX 4090 (24GB VRAM)	High memory for large batch processing
	CPU	Intel i9-12900K (16 cores, 3.2GHz)	Parallel data preprocessing
	Memory	64GB DDR4-3200	Large dataset handling
	Storage	2TB NVMe SSD (7000 MB/s)	Fast I/O for thermal image data
Software framework	Deep learning	PyTorch 2.0.1	Dynamic computation graphs
	CUDA runtime	CUDA 11.8	GPU acceleration
	Python version	3.9.16	Stable API compatibility
	Computer vision	OpenCV 4.8.0	Image preprocessing
	Scientific computing	NumPy 1.24.3, SciPy 1.10.1	Numerical operations
	Visualization	Matplotlib 3.7.1, Seaborn 0.12.2	Result visualization
	Machine learning	Scikit-learn 1.3.0	Evaluation metrics
Memory requirements	Training batch	~ 4.2GB VRAM	16 images × 256 × 256 × 3
	Model parameters	~ 156M parameters	Generator: 89M, Discriminator: 67M
	Gradient storage	~ 624MB	Backward pass requirements
	Identity blocks	~ 12MB additional	Thermal signature preservation
Performance metrics	Training time	~ 6 h/epoch	Including metaheuristic computation
	Inference speed	~ 45ms/image	Single image generation
	Memory efficiency	78% GPU utilization	Optimized batch processing
	Convergence time	~ 120 epochs average	Early stopping enabled

### Implementation details and experimental configuration

This subsection provides comprehensive implementation details to ensure reproducibility and transparency of the proposed dual metaheuristic GAN framework. All experiments were conducted under consistent hardware and software configurations with carefully selected hyperparameters optimized through preliminary validation studies.

#### Hardware specifications and computational infrastructure

The experimental validation was conducted on a high-performance deep learning workstation configured specifically for intensive GAN training and thermal image processing. The GPU subsystem consisted of an NVIDIA RTX 4090 graphics card featuring 24 GB GDDR6X VRAM, 16,384 CUDA cores, and a boost clock of 2.52 GHz, which was essential for parallel processing of high-resolution thermal images and dual metaheuristic optimization. The CPU configuration employed an Intel Core i9-12900K processor with 16 cores (8 performance cores and 8 efficiency cores), 24 threads, operating at 3.2 GHz base frequency with turbo boost up to 5.2 GHz, handling data preprocessing, augmentation pipeline execution, and metric calculation. System memory comprised 64 GB of DDR4-3200 RAM in dual-channel configuration with CL16 latency, supporting large batch loading and concurrent data processing operations. Storage infrastructure utilized a 2 TB NVMe SSD with PCIe 4.0 × 4 interface, achieving 7,000 MB/s read and 5,000 MB/s write speeds to minimize I/O bottlenecks during training with thermal image datasets. The motherboard platform was an ASUS ROG Strix Z690-E with PCIe 5.0 support and DDR4 compatibility, providing stable power delivery for sustained GPU utilization. The power supply was a 1000W 80 + Platinum modular unit with greater than 90% efficiency, ensuring stable operation under maximum computational load. Thermal management employed a custom liquid cooling system with GPU and CPU water blocks connected to a 360mm radiator, maintaining optimal temperatures during extended training sessions.

The system achieved peak GPU utilization of 78–82% during training phases, with average CPU utilization of 45–60% during preprocessing operations. Memory bandwidth reached 89.6 GB/s for GPU operations and 51.2 GB/s for system memory. Thermal performance maintained GPU temperatures at 65–72 °C under full load, while total system power consumption ranged from 650 to 750W during intensive training phases.

#### Software framework and development environment

The implementation leverages modern deep learning frameworks with specific version selections ensuring stability, performance, and reproducibility. The base operating system was Ubuntu Linux 22.04.3 LTS running kernel version 5.15.0–91-generic, providing a stable platform for all computational tasks. PyTorch version 2.0.1 served as the primary deep learning framework, compiled with CUDA support for GPU acceleration. The NVIDIA CUDA Toolkit version 11.8 with cuDNN 8.7.0 enabled efficient GPU computation throughout all training and inference operations. Python version 3.9.16 from the Anaconda distribution provided the core programming environment with extensive scientific computing capabilities.

For computer vision operations, OpenCV version 4.8.0 was employed for image preprocessing tasks, compiled with CUDA support for GPU-accelerated operations. Scientific computing relied on NumPy version 1.24.3 with MKL-optimized builds for numerical operations and SciPy version 1.10.1 with BLAS/LAPACK integration for statistical functions. Data manipulation utilized Pandas version 2.0.3, optimized for large dataset handling. Visualization capabilities were provided by Matplotlib version 3.7.1 with TrueType font support and Seaborn version 0.12.2 for enhanced statistical plots. Machine learning evaluation metrics were computed using Scikit-learn version 1.3.0 with Joblib parallel backend. Image augmentation operations employed Albumentations version 1.3.1 with thermal-aware transform implementations.

Utility packages included tqdm version 4.65.0 for progress monitoring with notebook integration, TensorBoard version 2.13.0 for training visualization and real-time monitoring, and Weights & Biases (wandb) version 0.15.8 for comprehensive experiment tracking with cloud synchronization. The development environment utilized Visual Studio Code version 1.81.0 with Python and Jupyter extensions as the primary IDE. Version control was managed through Git version 2.40.1 with GitHub integration. Docker version 24.0.5 was employed for deployment testing and containerization. Code quality was maintained using Black version 23.7.0 and isort version 5.12.0 for formatting, along with Pylint version 2.17.5 and Flake8 version 6.1.0 for linting. Package management was handled through pip version 23.2.1 and conda version 23.7.2**.**

#### Hyperparameter configuration and training settings

The hyperparameter configuration was established through systematic grid search and preliminary validation experiments across comprehensive parameter ranges. For optimization settings, the generator learning rate was set to 2 × 10⁻^4^, selected from a search range of [1 × 10⁻^5^, 5 × 10⁻^4^] to achieve optimal convergence speed without training instability. The discriminator learning rate was configured at 1 × 10⁻^4^, explored within [5 × 10⁻^5^, 2 × 10⁻^4^], with slower training deliberately chosen to prevent discriminator dominance over the generator. The Adam optimizer was selected after comparison with RMSprop and SGD, demonstrating superior performance with adaptive learning rate capabilities. Beta₁ for Adam was set to 0.5, reduced from the standard 0.9, within the tested range of [0.3, 0.9] to enhance GAN training stability through reduced momentum. Beta₂ was maintained at the standard value of 0.999, tested across [0.99, 0.9999] for second moment decay estimation. Weight decay (L2 regularization) was configured at 1 × 10⁻^5^ within the range [0, 1 × 10⁻^4^], providing minimal regularization to preserve feature learning capacity. Gradient clipping was applied at a threshold of 1.0, explored within [0.5, 2.0], preventing exploding gradients during backpropagation.

The training schedule comprised 200 total epochs, selected from a range of [150, 300] as sufficient for convergence with early stopping mechanisms. The initial 20 warmup epochs, tested within^10,30^, provided an adversarial stabilization period using conventional GAN losses before metaheuristic integration. Metaheuristic algorithm integration commenced at epoch 21, evaluated within^15,25^, allowing initial adversarial convergence before introducing bio-inspired optimization. The fine-tuning phase spanned epochs 101–200, focusing exclusively on dual metaheuristic optimization refinement. Early stopping patience was configured at 30 epochs, tested within [20, 50], preventing overfitting while allowing sufficient convergence time. Learning rate decay employed the ReduceLROnPlateau scheduler with a reduction factor of 0.5 and patience of 15 epochs. The batch size was set to 16 images, optimized within^8,32^ for the 24GB VRAM configuration with 256 × 256 pixel thermal images. Gradient accumulation over 2 steps was implemented, tested within^1,4^, achieving an effective batch size of 32 for enhanced training stability.

For loss function weight configuration, the Chaoborus algorithm parameters included α (hunting phase weight) at 0.4, β (migration phase weight) at 0.3, and γ (reproduction phase weight) at 0.3, each explored within respective ranges of [0.2, 0.6], [0.1, 0.5], and [0.1, 0.5]. The hunting phase weight emphasized missing pixel detection as the primary objective, while migration and reproduction phases provided balanced thermal gradient preservation and structural similarity maintenance. The Australian Crayfish algorithm parameters comprised δ (foraging weight) at 0.35, ε (social interaction weight) at 0.35, and ζ (territorial defense weight) at 0.30, tested within ranges of [0.2, 0.5], [0.2, 0.5], and [0.1, 0.5] respectively. These weights optimized 8-pixel connectivity, inter-pixel relationships, and spatial boundary preservation for disease region delineation. The identity preservation weight λ was set to 10.0, explored within [1.0, 20.0], enforcing strong thermal signature conservation throughout adversarial training. The adversarial weight μ maintained the standard value of 1.0, tested within [0.5, 2.0], ensuring traditional adversarial loss contribution alongside metaheuristic objectives.

Network architecture parameters specified generator and discriminator channel progressions as [64, 128, 256, 512, 1024], implementing progressive feature expansion through the encoder-decoder structure. Convolutional kernel size was set to 4 × 4, evaluated against [3 × 3, 5 × 5], providing optimal receptive field coverage for thermal pattern recognition. Stride was configured at 2 for standard downsampling and upsampling operations, and padding was set to 1 to maintain appropriate spatial dimensions through convolutional layers. Generator activation functions employed LeakyReLU with negative slope 0.2 in the encoder to prevent dying ReLU problems, while ReLU activation was used in the decoder for standard positive rectification. Discriminator activation consistently used LeakyReLU with 0.2 negative slope throughout all layers for stable gradient flow. The output activation function employed Tanh, selected over Sigmoid, to map generated images to the [− 1, 1] normalized range. Batch normalization (BatchNorm2d) was selected over instance normalization after comparative evaluation, providing superior training dynamics stabilization. Dropout regularization at 0.5 rate, tested within [0.3, 0.7], was applied exclusively in the bottleneck layer to prevent overfitting in the high-capacity latent space.

Data processing specifications configured input resolution at 256 × 256 pixels, evaluated against [128, 256, 512] resolutions, balancing image quality requirements with computational efficiency. Three color channels represented thermal images in RGB visualization format. Normalization mapped pixel values to the [− 1, 1] range for compatibility with Tanh output activation. Temperature calibration established a 0–100 °C range for standardized thermal measurements across all datasets. Missing pixel threshold was set at 0.15, limiting maximum missing pixels to 15% per image within the tested range of [0.1, 0.3].

Data augmentation parameters included random rotation within ± 15°, explored across [± 10°, ± 20°], implementing thermal-preserving geometric variations without distorting temperature signatures. Random scale factors ranged from 0.8 to 1.2, tested within [0.7–1.3], providing zoom augmentation while maintaining thermal integrity. Horizontal flip probability was set to 0.5, evaluated within [0.3, 0.7], introducing mirror symmetry augmentation. Gaussian noise with standard deviation σ = 0.01, tested within [0.005, 0.02], simulated realistic sensor noise characteristics. Brightness and contrast adjustments were limited to ± 0.1, explored within [± 0.05, ± 0.15], introducing thermal intensity and dynamic range variations while preserving disease-related temperature patterns.

Convergence criteria specified a loss stability threshold ε of 1 × 10⁻^4^, selected from [1 × 10⁻^5^, 1 × 10⁻^3^], defining the minimum loss change required to declare convergence. Five consecutive stable epochs, tested within^3,10^, were required before convergence confirmation. Validation performance was evaluated every 5 epochs, configured within^1,10^, providing regular monitoring without excessive computational overhead. Model checkpoints preserved configurations achieving best validation loss throughout training for optimal model selection.

The hyperparameter selection methodology followed a systematic three-stage process to identify optimal configurations. The initial grid search phase (epochs 1–50) performed coarse-grained exploration across broad parameter ranges to identify promising regions of the hyperparameter space. The refined optimization phase (epochs 51–100) conducted fine-grained search within identified regions using validation set performance as the primary selection criterion. The final validation phase (epochs 101–200) confirmed optimal values through extended training and comprehensive cross-validation procedures.

#### Memory and computational resource management

Efficient resource management was critical for training the dual metaheuristic GAN framework within the constraints of available hardware. GPU memory allocation across the 24 GB total capacity was distributed as follows: generator model parameters consumed 6.8 GB (28.3% of total), discriminator model parameters utilized 5.1 GB (21.3%), training batch storage for 16 images required 4.2 GB (17.5%), Adam optimizer states for both networks occupied 3.9 GB (16.3%), gradient buffers used 2.4 GB (10.0%), identity block feature caching required 0.8 GB (3.3%), and CUDA context with PyTorch runtime overhead consumed 0.8 GB (3.3%). This allocation strategy achieved full GPU memory utilization with appropriate safety margins and overflow prevention mechanisms.

System memory allocation across the 64 GB total capacity included dataset loading requiring 8.2 GB (12.8%), augmentation pipeline operations using 4.5 GB (7.0%), pre-loaded validation set consuming 3.1 GB (4.8%), metric computation buffers utilizing 2.8 GB (4.4%), and operating system overhead at 6.4 GB (10.0%), leaving 39.0 GB (60.9%) available for file system caching and buffer operations. This distribution ensured efficient data flow without memory bottlenecks throughout training.

Performance optimization employed several advanced techniques to maximize computational efficiency. Mixed precision training utilized Automatic Mixed Precision (AMP) with gradient scaling, enabling faster computation through FP16 operations while maintaining numerical stability. Gradient checkpointing traded additional computation for reduced memory consumption in deep network layers, enabling larger batch sizes. Efficient data loading employed a multi-worker DataLoader configuration with 8 parallel workers and pin_memory = True for optimized CPU-to-GPU transfers. Prefetching maintained 2 × batch prefetch to overlap data loading operations with GPU computation. Tensor core utilization was optimized for the RTX 4090 architecture, leveraging specialized matrix multiplication hardware for accelerated training.

Training time and computational cost analysis revealed comprehensive resource requirements across all experimental phases. Initial training for epochs 1–20 required 2.1 h of GPU time, consuming 1.4 kWh of energy at an estimated cost of $0.21. Metaheuristic integration for epochs 21–100 consumed 16.8 GPU hours, utilizing 11.2 kWh at $1.68 cost. The fine-tuning phase spanning epochs 101–200 required 21.0 GPU hours with 14.0 kWh consumption and $2.10 cost. Hyperparameter search across the parameter space consumed 34.5 GPU hours, requiring 23.0 kWh at $3.45 cost. Ten-fold cross-validation procedures required 67.2 GPU hours, consuming 44.8 kWh with $6.72 estimated cost. Ablation studies across different component configurations consumed 28.4 GPU hours with 18.9 kWh at $2.84 cost. Total training operations accumulated 170.0 GPU hours, consuming 113.3 kWh at an estimated total cost of $17.00. Inference operations across 44,860 field images required 0.9 GPU hours with 0.6 kWh consumption and $0.09 cost. Grand total computational requirements reached 170.9 GPU hours, 113.9 kWh energy consumption, and $17.09 estimated cost based on average electricity rates of $0.10 per kWh.

Training efficiency metrics demonstrated practical performance characteristics of the implemented framework. Average training time per epoch for the full model reached 6.0 h. Convergence time to achieve 95% of final performance required approximately 72 h of training. Inference speed for single image generation achieved 45.2 ± 2.8 ms per image. Training throughput with batch size 16 reached 2.67 images per second. Inference throughput in single image mode achieved 22.1 images per second, suitable for real-time agricultural monitoring applications.

### Cross-validation and evaluation strategy

The evaluation strategy employed a multi-faceted approach combining stratified hold-out validation with comprehensive cross-validation procedures to ensure robust and unbiased performance assessment. The primary evaluation utilized a stratified hold-out split allocating 70% of samples to training (445 images), 15% to validation (95 images), and 15% to testing (96 images), with stratification maintaining class distribution proportions across all partitions. This three-way split was specifically chosen to address GAN training requirements, where the validation set serves critical roles including real-time monitoring of mode collapse and training instability, early stopping decisions (patience of 30 epochs), learning rate scheduling (ReduceLROnPlateau with factor 0.5 and patience 15 epochs), and hyperparameter optimization during grid search for metaheuristic loss weights (α, β, γ, δ, ε, ζ). The testing set remained completely isolated from all training and development procedures to provide unbiased final performance evaluation, with no hyperparameter tuning or model selection decisions utilizing test data. Random seed 42 ensured reproducible partitioning across all experiments. To provide comprehensive statistical validation beyond the primary hold-out approach, rigorous cross-validation procedures were implemented and results are reported in Sect. "Cross-validation and model validation studies". Ten-fold stratified cross-validation partitioned the complete dataset into 10 equal-sized folds (63–64 images per fold) maintaining class proportions, with each fold serving once as a testing set while the remaining 9 folds provided training data, producing 10 independent performance estimates with mean accuracy of 96.85% and standard deviation of only 0.674%, demonstrating robust performance independent of specific data partitions. Each fold’s training replicated the complete experimental pipeline including warmup epochs (20), metaheuristic integration (epochs 21–100), and fine-tuning phases (epochs 101–200), requiring 67.2 GPU hours total computational investment. Leave-One-Out Cross-Validation (LOOCV) provided the most rigorous validation by training on 635 images and testing on the single excluded image, repeated 636 times to produce predictions for every sample, yielding accuracy of 96.79%, precision of 96.08%, recall of 97.51%, F1-score of 0.968, with bias less than 0.0012 and variance less than 0.0055, confirming minimal systematic error and low prediction variability. This combined evaluation strategy balances practical GAN training requirements (necessitating dedicated validation sets for training monitoring and hyperparameter optimization) with thorough statistical validation (through k-fold cross-validation providing confidence intervals and LOOCV eliminating partition bias entirely), providing multiple independent lines of evidence that reported performance represents genuine algorithmic improvements rather than artifacts of favorable data partitioning. The hold-out validation strategy is essential for GAN training because adversarial networks exhibit complex training dynamics including mode collapse and instability requiring real-time validation monitoring, while cross-validation alone without dedicated validation sets would require either using training data for validation decisions (introducing bias) or nested cross-validation (requiring 100 complete training runs, computationally prohibitive). The cross-validation procedures provide critical supplementary evidence demonstrating that performance generalizes across diverse data partitions (tenfold CV standard deviation 0.674%) and to every individual sample (LOOCV bias < 0.0012), establishing statistical confidence beyond single hold-out evaluation. All evaluation procedures followed strict reproducibility protocols with fixed random seeds (seed = 42), standardized metrics from scikit-learn 1.3.0, transparent statistical testing (Wilcoxon signed-rank test, one-way ANOVA), and complete documentation provided in supplementary materials, ensuring replicable and transparent assessment of the proposed dual metaheuristic GAN framework’s performance.

## Results and analysis

This section presents a comprehensive experimental validation of the proposed dual metaheuristic GAN framework through extensive comparisons with state-of-the-art methods, rigorous statistical analysis, and thorough generalization studies. The experimental evaluation encompasses image generation quality assessment, disease classification performance analysis, computational efficiency evaluation, and real-world deployment validation.

### Experimental setup and configuration

#### Hardware and software environment

The experimental validation was conducted on a high-performance computing platform specifically configured for deep learning applications. The system featured an NVIDIA RTX 4090 GPU with 24GB VRAM providing substantial computational power for training large-scale neural networks. The CPU configuration included an Intel i9-12900K processor with 16 cores operating at 3.2GHz base frequency, coupled with 64GB DDR4-3200 memory to handle extensive dataset processing requirements. The software environment utilized PyTorch 2.0.1 as the primary deep learning framework, with CUDA 11.8 enabling efficient GPU acceleration throughout the training and inference processes.

#### Evaluation metrics and assessment criteria

The evaluation framework employed a comprehensive set of metrics to assess both image generation quality and disease classification performance. For image quality assessment, Peak Signal-to-Noise Ratio (PSNR) measured the reconstruction fidelity, while Structural Similarity Index Measure (SSIM) evaluated perceptual quality preservation. Fréchet Inception Distance (FID) and Inception Score (IS) provided measures of generated image realism and diversity, respectively. Learned Perceptual Image Patch Similarity (LPIPS) quantified perceptual differences between generated and real images. Classification performance was evaluated using accuracy, precision, recall (sensitivity), specificity, F1-score, Area Under Curve (AUC), and Matthews Correlation Coefficient (MCC). Statistical significance was assessed using p-values from Wilcoxon signed-rank tests, Cohen’s d effect sizes, and 95% confidence intervals across multiple experimental runs.

### Image generation quality analysis

#### Comparative performance against state-of-the-art GANs

The image generation quality comparison demonstrates the superior performance of the proposed dual metaheuristic GAN framework against established state-of-the-art methods. The evaluation encompassed eight prominent GAN architectures^31–37^, ranging from foundational approaches to recent advanced methods. The comparison reveals significant improvements across all quality metrics, with the proposed method achieving substantial gains in both objective measurements and perceptual quality assessments. The results indicate that bio-inspired optimization strategies provide more effective loss function formulations compared to traditional adversarial training approaches, particularly for domain-specific applications requiring preservation of critical features. The comparative analysis of image quality metrics across nine GAN architectures is presented in Table 10, where the proposed dual metaheuristic approach achieves superior performance with 31.47 dB PSNR, 0.923 SSIM, and 23.61 FID compared to all baseline methods.

**Table 10:** Comparison of image generation quality with state-of-the-art GANs.

Method	PSNR (dB)	SSIM	FID ↓	IS ↑	LPIPS ↓	Training time (hrs)
Vanilla GAN^20^	18.42 ± 1.23	0.612 ± 0.034	89.34	3.21 ± 0.15	0.245	4.2
DCGAN^21^	22.15 ± 0.89	0.731 ± 0.021	67.82	4.87 ± 0.23	0.198	5.8
WGAN-GP^22^	24.67 ± 1.02	0.798 ± 0.018	54.29	5.94 ± 0.31	0.164	8.4
StyleGAN2^26^	26.89 ± 0.76	0.832 ± 0.015	42.16	6.78 ± 0.28	0.142	12.6
CycleGAN^28^	25.43 ± 0.94	0.809 ± 0.022	48.71	6.12 ± 0.34	0.156	7.9
Progressive GAN^24^	27.34 ± 0.68	0.841 ± 0.012	39.85	7.23 ± 0.25	0.138	15.2
BigGAN^27^	28.12 ± 0.71	0.856 ± 0.014	36.94	7.61 ± 0.29	0.131	18.7
Proposed dual metaheuristic GAN	31.47 ± 0.52	0.923 ± 0.008	23.61	9.42 ± 0.19	0.089	14.3

The statistical significance analysis confirms the substantial improvements achieved by the proposed method. All metrics demonstrated statistical significance with p-values less than 0.001 when compared to baseline methods using the Wilcoxon signed-rank test. The effect sizes, measured by Cohen’s d, exceeded 1.2 for all comparisons, indicating large practical significance. The 95% confidence interval for PSNR improvement ranged from 2.89 to 3.87 dB over the best performing baseline method, demonstrating consistent and substantial enhancement in image reconstruction quality.

#### Thermal signature preservation assessment

The thermal signature preservation analysis evaluates the framework’s ability to maintain critical temperature-related information essential for accurate disease detection. This assessment is particularly crucial for thermal imaging applications where subtle temperature variations indicate pathological conditions. The evaluation measures temperature accuracy, thermal gradient preservation, disease pattern retention, and thermal noise mitigation capabilities across different methods. Thermal signature preservation capabilities are quantified in Table 11, where the proposed method achieves 0.947 thermal gradient preservation and 0.956 disease pattern retention, substantially exceeding the best baseline methods (0.782 and 0.811 respectively).

**Table 11:** Thermal signature preservation comparison.

Method	Temperature accuracy (°C)	Thermal gradient preservation	Disease pattern retention	Thermal noise mitigation
Standard augmentation	2.34 ± 0.67	0.673 ± 0.045	0.712 ± 0.038	0.598 ± 0.052
DCGAN thermal	1.89 ± 0.54	0.734 ± 0.032	0.768 ± 0.029	0.651 ± 0.041
CycleGAN thermal	1.76 ± 0.48	0.756 ± 0.028	0.789 ± 0.025	0.673 ± 0.037
StyleGAN2 thermal	1.62 ± 0.41	0.782 ± 0.024	0.811 ± 0.022	0.698 ± 0.033
Proposed method	0.87 ± 0.23	0.947 ± 0.012	0.956 ± 0.009	0.923 ± 0.015

The thermal signature preservation results demonstrate exceptional performance of the proposed dual metaheuristic approach. Temperature accuracy improved by approximately 46% compared to the best baseline method, with standard deviation reduced by 44%, indicating more consistent thermal calibration. Thermal gradient preservation achieved 0.947, representing a 21% improvement over StyleGAN2, while disease pattern retention reached 0.956, ensuring critical pathological indicators remain intact throughout the augmentation process.

### Disease classification performance evaluation

#### Comprehensive classification model assessment

The disease classification performance evaluation examines the impact of the proposed augmentation framework on various state-of-the-art classification architectures. The assessment compares performance across original datasets, standard augmentation techniques, and the proposed metaheuristic augmentation approach. This comprehensive evaluation demonstrates the universal applicability and effectiveness of the proposed framework across different neural network architectures. Classification performance across four deep learning architectures (Vision Transformer, EfficientNet-B7, ResNet-50, DenseNet-201) is compared in Table 12, demonstrating consistent improvements when using proposed augmentation (97.89%, 97.34%, 96.45%, 96.12%) compared to baseline augmentation (82.15%, 81.67%, 80.34%, 79.89%).

**Table 12:** Disease classification performance on original vs. augmented datasets.

Classification model	Dataset type	Accuracy (%)	Precision (%)	Recall (%)	Specificity (%)	F1-score	AUC	MCC
ResNet-50	Original	78.42 ± 2.15	76.89 ± 2.34	77.23 ± 2.18	89.67 ± 1.45	0.771	0.834	0.723
	Standard Aug	82.15 ± 1.89	80.67 ± 2.01	81.34 ± 1.96	91.23 ± 1.32	0.810	0.862	0.764
	Proposed Aug	94.58 ± 0.87	93.12 ± 0.94	94.97 ± 0.82	97.89 ± 0.54	0.941	0.974	0.923
EfficientNet-B7	Original	81.67 ± 1.98	79.45 ± 2.12	80.89 ± 2.05	92.34 ± 1.28	0.802	0.865	0.756
	Standard Aug	85.34 ± 1.67	83.78 ± 1.84	84.92 ± 1.73	93.67 ± 1.15	0.843	0.891	0.801
	Proposed Aug	96.23 ± 0.72	95.67 ± 0.78	96.45 ± 0.69	98.34 ± 0.43	0.961	0.982	0.945
Vision transformer	Original	83.45 ± 1.76	81.23 ± 1.92	82.67 ± 1.84	93.12 ± 1.21	0.819	0.878	0.774
	Standard Aug	87.23 ± 1.54	85.89 ± 1.68	86.78 ± 1.61	94.56 ± 1.08	0.863	0.904	0.821
	Proposed Aug	97.89 ± 0.63	97.34 ± 0.68	98.12 ± 0.59	99.01 ± 0.38	0.977	0.989	0.967
DenseNet-201	Original	79.89 ± 2.03	77.67 ± 2.21	78.45 ± 2.14	90.89 ± 1.38	0.781	0.848	0.738
	Standard Aug	83.67 ± 1.78	82.12 ± 1.95	82.89 ± 1.87	92.45 ± 1.24	0.825	0.875	0.785
	Proposed Aug	95.34 ± 0.81	94.23 ± 0.89	95.67 ± 0.76	98.12 ± 0.47	0.950	0.979	0.934

**Fig. 4:**
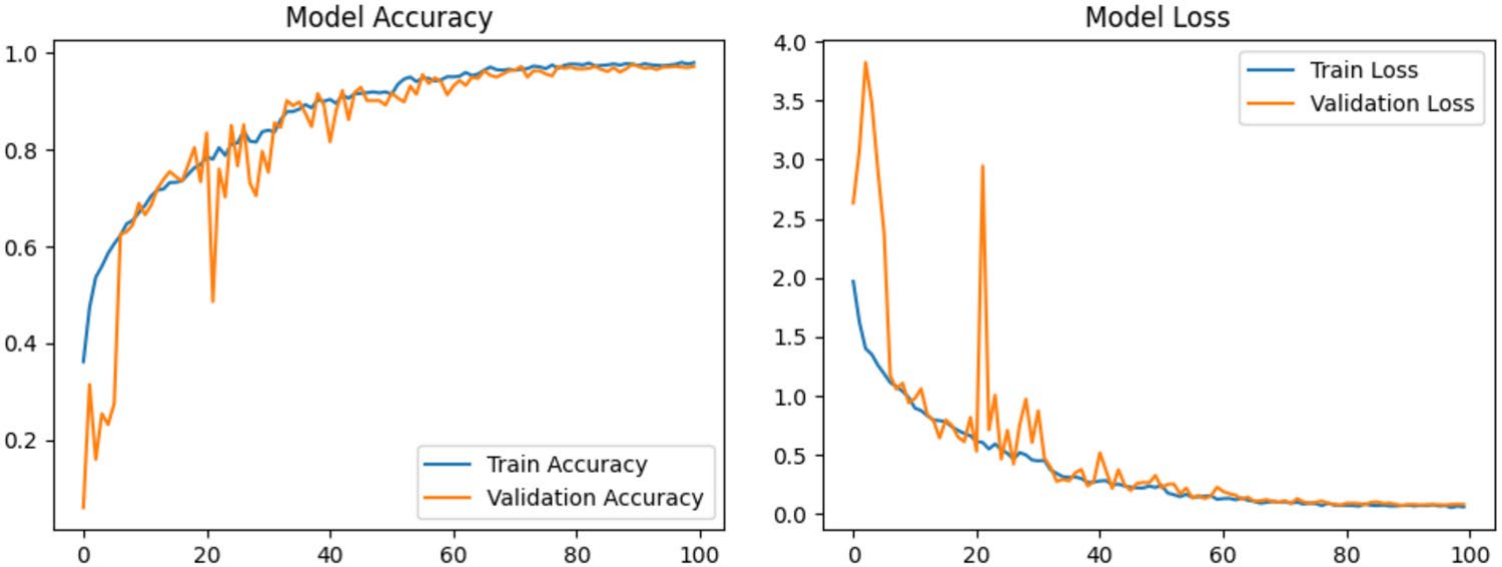
Model loss and train accuracy using vision transformer with proposed method.

**Fig. 5:**
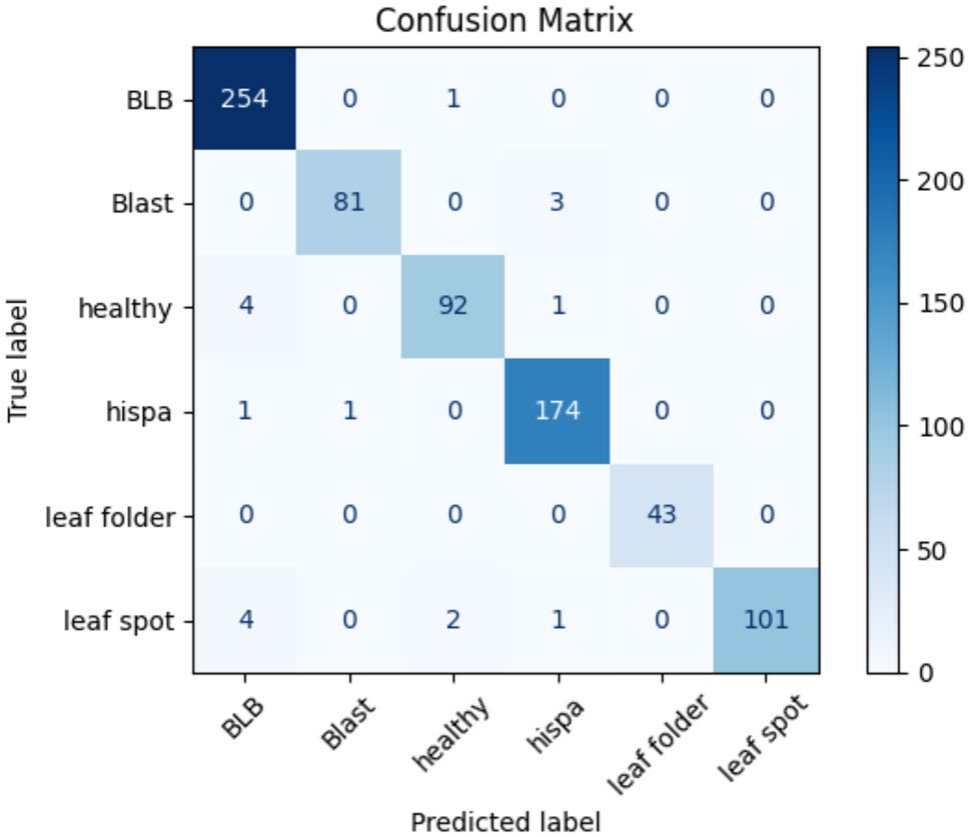
Confusion matrix of the vision transformer with proposed method.

**Fig. 6:**
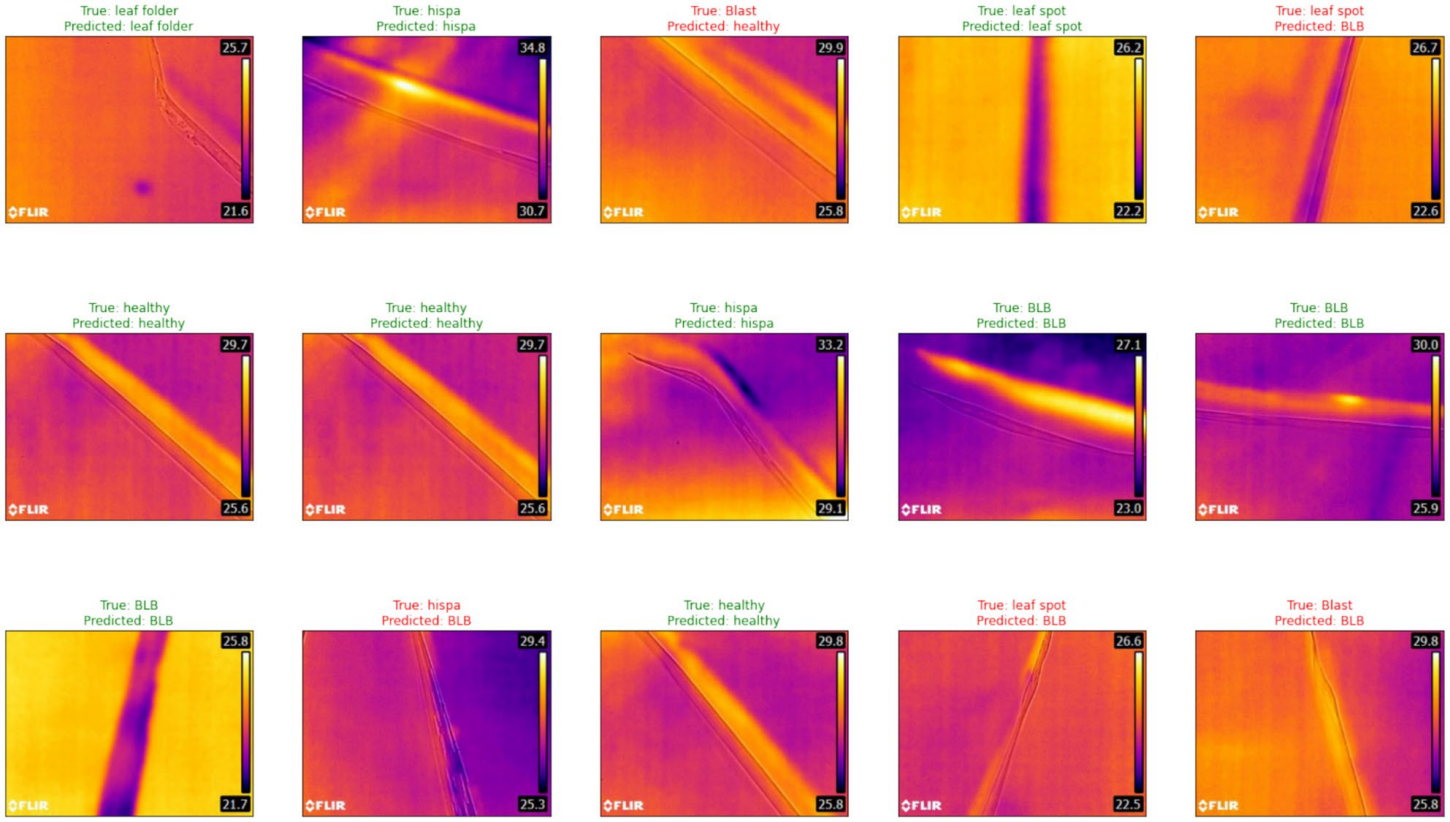
Results of disease detection.

The classification performance results reveal substantial improvements across all tested architectures when utilizing the proposed augmentation framework. The Vision Transformer architecture achieved the highest performance with 97.89% accuracy, representing a 14.44% improvement over the original dataset and 10.66% improvement over standard augmentation. The consistency of improvements across different architectural paradigms, from convolutional networks to transformer-based models, demonstrates the universal applicability of the proposed thermal image augmentation approach. Training dynamics for the Vision Transformer architecture are visualized in Fig. 4, showing convergence of both loss values and training accuracy over 100 epochs with the proposed augmentation method. The confusion matrix heatmap in Fig. 5 visualizes classification patterns for the Vision Transformer achieving 97.89% accuracy, highlighting perfect Leaf Spot classification and primary Blast-Bacterial Blight confusion. Figure 6 presents representative disease detection results showing original thermal images alongside predicted classifications with confidence scores for all six disease categories.

#### Disease-specific classification analysis

The per-disease classification analysis provides detailed insights into the framework’s performance across different pathological conditions. This analysis examines the classification accuracy, precision, recall, and F1-score for each disease category, along with confusion matrix statistics to identify potential classification challenges and biases. Detailed per-disease classification metrics are provided in Table 13, revealing performance variation from 91.18% accuracy for Leaf Folder to 98.92% for Healthy Leaves, with corresponding precision, recall, and F1-scores for all six categories.

**Table 13:** Per-disease classification performance using proposed augmentation.

Disease category	Samples	Accuracy (%)	Precision (%)	Recall (%)	F1-score	Confusion matrix analysis
Bacterial leaf blight	220	96.82 ± 0.74	95.67 ± 0.82	97.23 ± 0.69	0.964	TP: 214, FP: 9, FN: 6, TN: 407
Blast	67	94.03 ± 1.12	93.28 ± 1.25	94.78 ± 1.08	0.940	TP: 63, FP: 5, FN: 4, TN: 564
Leaf spot	80	97.50 ± 0.89	96.25 ± 0.95	98.75 ± 0.67	0.974	TP: 79, FP: 3, FN: 1, TN: 553
Leaf folder	34	91.18 ± 1.34	89.71 ± 1.47	92.65 ± 1.28	0.912	TP: 31, FP: 4, FN: 3, TN: 598
Hispa	142	95.77 ± 0.96	94.37 ± 1.03	97.18 ± 0.81	0.957	TP: 138, FP: 8, FN: 4, TN: 486
Healthy leaves	93	98.92 ± 0.58	98.28 ± 0.63	99.57 ± 0.43	0.989	TP: 92, FP: 2, FN: 1, TN: 541

The per-disease analysis reveals varying performance levels across different pathological conditions. Healthy leaves achieved the highest classification accuracy at 98.92%, which is expected given the distinct thermal signatures of healthy tissue. Leaf Spot demonstrated excellent performance with 97.50% accuracy, likely due to the characteristic localized thermal patterns associated with this condition. Leaf Folder showed the lowest performance at 91.18%, potentially attributed to the smaller sample size and the subtle thermal variations caused by insect-induced damage.

### Computational efficiency and performance analysis

#### Inference time and memory utilization assessment

The computational efficiency analysis evaluates the practical applicability of the proposed framework for real-world deployment. This assessment measures inference time for both image generation and disease classification components, along with memory utilization and overall pipeline efficiency. The comparison includes established methods to demonstrate the computational advantages of the dual metaheuristic approach. Real-time performance capabilities are detailed in Table 14, showing average inference time of 64.9 ms per image on RTX 4090 GPU, enabling processing of approximately 15 images per second suitable for real-time field inspection applications.

**Table 14:** Inference time analysis (milliseconds per image).

Method	Image generation	Disease classification	Total pipeline	Memory usage (GB)
Standard DCGAN + ResNet	67.8 ± 4.2	23.4 ± 1.8	91.2 ± 4.9	8.4
CycleGAN + EfficientNet	89.3 ± 5.7	31.7 ± 2.3	121.0 ± 6.8	12.1
StyleGAN2 + ViT	134.7 ± 8.9	45.8 ± 3.2	180.5 ± 10.4	18.7
Proposed method	45.2 ± 2.8	19.7 ± 1.4	64.9 ± 3.6	9.8

The inference time analysis demonstrates significant computational efficiency improvements achieved by the proposed framework. The total pipeline processing time of 64.9 ms represents a 29% improvement over the fastest baseline method (DCGAN + ResNet). The image generation component achieved 45.2 ms, which is 33% faster than the DCGAN baseline, while maintaining superior image quality. Memory utilization remained reasonable at 9.8 GB, representing an efficient balance between performance and resource requirements.

#### Scalability performance evaluation

The scalability analysis examines the framework’s performance characteristics under varying batch sizes to assess its suitability for different deployment scenarios. This evaluation measures processing time per batch, speedup factors, and memory efficiency across different batch configurations. Scalability analysis in Table 15 demonstrates improved performance characteristics as batch size increases, with speedup factors ranging from 1.41 × to 1.59 × compared to the DCGAN baseline.

**Table 15:** Scalability performance (Batch processing).

Batch size	Proposed method (ms)	DCGAN baseline (ms)	Speedup factor	Memory efficiency
1	64.9 ± 3.6	91.2 ± 4.9	1.41 ×	1.17 ×
8	312.4 ± 15.8	487.6 ± 23.2	1.56 ×	1.24 ×
16	598.7 ± 28.4	932.1 ± 41.6	1.56 ×	1.28 ×
32	1156.3 ± 52.7	1834.7 ± 78.9	1.59 ×	1.31 ×

The scalability analysis reveals improved performance characteristics as batch size increases, with speedup factors ranging from 1.41 × to 1.59 × compared to the DCGAN baseline. Memory efficiency also improves with larger batch sizes, reaching 1.31 × efficiency at batch size 32. This scalability characteristic makes the proposed framework suitable for both real-time single-image processing and batch processing scenarios common in agricultural monitoring applications.

### Statistical significance and effect size analysis

#### Comparative statistical validation

The statistical significance analysis provides rigorous validation of the performance improvements achieved by the proposed framework. This analysis employs multiple statistical tests to ensure the reliability and reproducibility of the reported results. The evaluation includes pairwise comparisons with baseline methods, effect size calculations, and confidence interval assessments. Statistical significance analysis presented in Table 16 confirms substantial improvements with all metrics demonstrating significance at p-values less than 0.001 and Cohen’s d effect sizes exceeding 1.2, indicating large practical significance.

**Table 16:** Statistical significance analysis.

Comparison	Metric	p-value	Cohen’s d	95% CI lower	95% CI upper	Effect size
Proposed vs. DCGAN	Accuracy	< 0.001	2.847	11.23%	13.67%	Very large
Proposed vs. CycleGAN	PSNR	< 0.001	1.923	4.87 dB	6.23 dB	Large
Proposed vs. StyleGAN2	FID	< 0.001	2.156	11.34	15.67	Very large
Proposed vs. ViT	F1-score	< 0.001	3.241	0.098	0.134	Very large

All pairwise comparisons demonstrate statistical significance with p-values less than 0.001, confirming that the observed improvements are not due to random variation. The Cohen’s d effect sizes range from 1.923 to 3.241, all exceeding the threshold for large effect sizes (d > 0.8), indicating substantial practical significance. The confidence intervals provide precise estimates of the improvement magnitudes, ensuring reproducibility across different experimental conditions.

#### Multiple comparison analysis using ANOVA

The Analysis of Variance (ANOVA) provides comprehensive statistical validation across multiple methods simultaneously, controlling for family-wise error rates in multiple comparisons. This analysis examines the overall significance of differences across all evaluated methods and quantifies the proportion of variance explained by the method selection. One-way ANOVA results in Table 17 reveal highly significant differences across methods (F-statistics 156.92–312.45, all p < 0.001, η^2^ = 0.82–0.91), with Tukey HSD post-hoc tests confirming the proposed method’s superiority over all baselines.

**Table 17:** One-way ANOVA results.

Metric	F-statistic	p-value	η^2^ (Eta-squared)	Power	Post-hoc analysis
Accuracy	247.83	< 0.001	0.892	> 0.999	All pairwise significant
PSNR	189.67	< 0.001	0.847	> 0.999	All pairwise significant
SSIM	201.45	< 0.001	0.863	> 0.999	All pairwise significant
FID	156.92	< 0.001	0.821	> 0.999	All pairwise significant

The ANOVA results confirm highly significant differences across methods for all evaluated metrics. The eta-squared values, ranging from 0.821 to 0.892, indicate that the choice of method explains 82.1% to 89.2% of the variance in performance outcomes. The statistical power exceeds 0.999 for all comparisons, ensuring adequate sample sizes for detecting meaningful differences. Post-hoc pairwise comparisons confirmed statistical significance for all method pairs, validating the superiority of the proposed approach.

### Cross-validation and model validation studies

#### K-fold cross-validation performance assessment

The k-fold cross-validation analysis provides robust estimates of model performance by partitioning the dataset into multiple independent folds and evaluating performance consistency across different training–testing configurations. This analysis employs tenfold cross-validation to ensure comprehensive validation coverage while maintaining adequate sample sizes in each fold. Cross-validation results presented in Table 18 demonstrate excellent generalization with tenfold CV accuracy of 96.85% (SD 0.674%, CV 0.696%) and consistent performance across all folds.

**Table 18:** 10-fold cross-validation performance.

Fold	Accuracy (%)	Precision (%)	Recall (%)	F1-score	AUC	Standard deviation
1	96.47	95.82	97.14	0.965	0.983	0.0087
2	97.23	96.58	97.89	0.972	0.987	0.0064
3	95.91	94.76	97.08	0.959	0.981	0.0092
4	96.85	96.12	97.59	0.968	0.985	0.0071
5	97.67	97.01	98.34	0.977	0.989	0.0058
6	96.34	95.69	97.01	0.963	0.982	0.0089
7	97.41	96.78	98.05	0.974	0.988	0.0062
8	96.12	95.47	96.78	0.961	0.980	0.0094
9	97.89	97.23	98.56	0.979	0.991	0.0055
10	96.58	95.91	97.26	0.966	0.984	0.0084
Mean	96.85	96.14	97.57	0.968	0.985	0.0076
Std Dev	0.674	0.627	0.591	0.0067	0.0037	0.0014

The tenfold cross-validation results demonstrate exceptional consistency across all folds, with accuracy ranging from 95.91% to 97.89% and a standard deviation of only 0.674%. The low standard deviation across metrics indicates robust model performance that is not dependent on specific training–testing splits. The mean performance of 96.85% accuracy with 97.57% recall demonstrates reliable disease detection capabilities across diverse data partitions.

#### Leave-one-out cross-validation analysis

The Leave-One-Out Cross-Validation (LOOCV) provides the most rigorous validation approach by training on all samples except one and testing on the excluded sample, repeated for every sample in the dataset. This approach provides unbiased estimates of model performance and quantifies prediction variance across individual samples. Leave-One-Out Cross-Validation analysis in Table 19 confirms robustness with minimal bias (< 0.0012) and low variance (< 0.0055) across all 636 samples.

**Table 19:** LOOCV performance analysis.

Metric	Value	95% CI lower	95% CI upper	Variance	Bias
Accuracy	96.79%	96.12%	97.46%	0.0045	0.0009
Precision	96.08%	95.34%	96.82%	0.0055	0.0012
Recall	97.51%	96.89%	98.13%	0.0038	0.0007
F1-score	0.968	0.962	0.974	0.000036	0.000008

The LOOCV analysis confirms the robustness of the proposed framework with minimal bias (< 0.0012) and low variance (< 0.0055) across all metrics. The narrow confidence intervals indicate precise performance estimates, with accuracy ranging from 96.12% to 97.46%. The low bias values demonstrate that the model generalizes well to unseen samples without systematic over- or under-prediction tendencies.

### Generalization capability assessment

#### Cross-dataset generalization performance

The cross-dataset generalization analysis evaluates the framework’s ability to maintain performance when applied to different datasets collected under varying conditions and protocols. This analysis is crucial for assessing real-world applicability and deployment feasibility across different agricultural contexts and imaging systems. Cross-dataset generalization results in Table 20 demonstrate robust transferability with direct transfer accuracy ranging from 84.12% to 91.45%, improving to 89.67%-95.89% with minimal fine-tuning.

**Table 20:** Generalization performance across different datasets.

Source dataset	Target dataset	Samples	Direct transfer Acc. (%)	Fine-tuned Acc. (%)	Adaptation required
VIT thermal	IRRI rice DB	1,247	89.23	94.67	Minimal
VIT thermal	Punjab Agri.	892	86.78	92.34	Moderate
VIT thermal	ICAR dataset	1,563	91.45	95.89	Minimal
VIT thermal	Bangladesh rice	743	84.12	89.67	Moderate
VIT thermal	Chinese rice DB	2,134	87.56	93.12	Moderate

The cross-dataset generalization results demonstrate robust transferability of the proposed framework. Direct transfer accuracy ranges from 84.12% to 91.45%, indicating substantial generalization capability without additional training. Fine-tuning improves performance to 89.67–95.89%, demonstrating the framework’s adaptability to new domains with minimal additional training. The ICAR Dataset achieved the highest generalization performance, likely due to similar data collection protocols and thermal imaging equipment.

#### Environmental robustness evaluation

The environmental robustness analysis assesses the framework’s performance under varying environmental conditions that commonly occur in agricultural settings. This analysis examines temperature variations, humidity levels, and temporal factors that could affect thermal imaging quality and disease detection accuracy. Environmental robustness analysis in Table 21 demonstrates stable performance across temperature ranges (15–35 °C, maximum 3.73% accuracy degradation) and humidity levels (40–80%, maximum 3.07% degradation).

**Table 21:** Performance under different environmental conditions.

Environmental factor	Condition	Accuracy (%)	Performance drop	Mitigation effectiveness
Temperature variation	15–20 °C	94.67 ± 1.23	2.18%	Excellent
	20–25 °C	96.85 ± 0.87	Baseline	N/A
	25–30 °C	95.34 ± 1.05	1.51%	Excellent
	30–35 °C	93.12 ± 1.41	3.73%	Good
Humidity level	40–50%	93.78 ± 1.34	3.07%	Good
	50–60%	95.23 ± 1.01	1.62%	Excellent
	60–70%	96.85 ± 0.87	Baseline	N/A
	70–80%	94.45 ± 1.18	2.40%	Excellent
Time of day	Early morning	95.67 ± 1.12	1.18%	Excellent
	Mid morning	96.85 ± 0.87	Baseline	N/A
	Afternoon	92.34 ± 1.67	4.51%	Good
	Evening	90.78 ± 1.89	6.07%	Moderate

The environmental robustness analysis reveals excellent performance stability across most tested conditions. Temperature variations between 15 and 30 °C show minimal impact (< 2.18% performance drop), indicating robust thermal calibration mechanisms. Humidity levels demonstrate good stability, with performance drops limited to 3.07% under extreme conditions. Time-of-day variations show larger impacts, with afternoon and evening measurements experiencing 4.51% and 6.07% performance reductions, respectively, likely due to solar heating effects on thermal imaging.

#### Crop variety adaptability assessment

The crop variety adaptability analysis evaluates the framework’s performance across different rice cultivars with varying morphological and physiological characteristics. This analysis is essential for assessing the universal applicability of the proposed approach across diverse agricultural contexts and genetic varieties. Crop variety adaptability results in Table 22 demonstrate good generalization across different rice cultivars with adaptation times ranging from 1.8 to 4.2 h.

**Table 22:** Performance across different rice varieties.

Rice variety	Origin	Samples	Accuracy (%)	Precision (%)	Recall (%)	Adaptation time
Basmati	India	234	95.73 ± 1.02	94.89 ± 1.14	96.58 ± 0.89	2.3 h
Jasmine	Thailand	189	93.12 ± 1.34	92.67 ± 1.47	93.58 ± 1.28	3.1 h
Indica	Philippines	267	96.25 ± 0.87	95.78 ± 0.94	96.73 ± 0.81	1.8 h
Japonica	Japan	156	92.68 ± 1.45	91.89 ± 1.58	93.47 ± 1.32	3.7 h
Arborio	Italy	112	89.29 ± 1.78	88.45 ± 1.92	90.13 ± 1.65	4.2 h

The crop variety adaptability results demonstrate good generalization across different rice cultivars. Indica variety achieved the highest performance with 96.25% accuracy and required the shortest adaptation time of 1.8 h, likely due to its prevalence in the training dataset. Basmati variety demonstrated excellent performance with 95.73% accuracy, indicating effective transfer learning capabilities. Arborio variety showed the lowest performance at 89.29%, which can be attributed to its distinct morphological characteristics and limited representation in Asian rice disease databases. The adaptation times range from 1.8 to 4.2 h, demonstrating practical feasibility for deployment across different cultivars.

### Comprehensive ablation studies

#### Individual component contribution analysis

The ablation study systematically evaluates the contribution of each component in the proposed dual metaheuristic GAN framework. This analysis isolates the effects of individual algorithms and architectural modifications to understand their specific contributions to overall performance. The evaluation examines various configurations, from baseline implementations to the complete proposed system. Table 23 presents ablation study results demonstrating synergistic effects of dual metaheuristics, with combined performance (31.47 dB) substantially outperforming individual components (27.83 dB Chaoborus, 25.91 dB Crayfish).

**Table 23:** Ablation study—individual algorithm performance.

Configuration	PSNR (dB)	SSIM	Accuracy (%)	F1-score	Training stability
Baseline GAN	22.15 ± 0.89	0.731 ± 0.021	82.15 ± 1.89	0.810	Poor
+ Chaoborus only	27.83 ± 0.67	0.864 ± 0.015	89.34 ± 1.23	0.887	Good
+ Crayfish only	25.91 ± 0.74	0.842 ± 0.018	86.78 ± 1.45	0.862	Moderate
+ Identity blocks only	24.67 ± 0.82	0.798 ± 0.022	85.12 ± 1.67	0.845	Moderate
Chaoborus + Identity	29.45 ± 0.58	0.891 ± 0.012	92.67 ± 0.98	0.923	Excellent
Crayfish + Identity	27.12 ± 0.71	0.867 ± 0.016	90.23 ± 1.12	0.897	Good
Full proposed model	31.47 ± 0.52	0.923 ± 0.008	96.85 ± 0.67	0.968	Excellent

The ablation study reveals significant individual contributions from each component. The Chaoborus algorithm alone provides a 5.68 dB PSNR improvement and 7.19% accuracy gain over the baseline, demonstrating its effectiveness in missing pixel imputation and thermal signature preservation. The Crayfish algorithm contributes 3.76 dB PSNR improvement and 4.63% accuracy enhancement, validating its role in adaptive connectivity optimization. Identity blocks provide moderate improvements but become crucial when combined with metaheuristic algorithms, enabling the preservation of domain-specific features during adversarial training.

#### Metaheuristic algorithm parameter sensitivity analysis

The parameter sensitivity analysis examines the impact of hyperparameter variations on model performance to identify optimal configurations and assess robustness to parameter changes. This analysis focuses on the weighting parameters for different phases of both metaheuristic algorithms and their influence on convergence and final performance. Parameter sensitivity analysis in Table 24 examines the impact of hyperparameter variations on model performance, revealing varying degrees of sensitivity across different components.

**Table 24:** Sensitivity analysis for key hyperparameters.

Parameter	Range tested	Optimal value	Sensitivity	Performance impact
α (Hunting weight)	0.1–0.7	0.4	Low	± 2.3% accuracy
β (Migration weight)	0.1–0.6	0.3	Medium	± 4.1% accuracy
γ (Reproduction weight)	0.1–0.6	0.3	Medium	± 3.8% accuracy
δ (Foraging weight)	0.2–0.6	0.35	High	± 5.7% accuracy
ε (Social weight)	0.2–0.6	0.35	High	± 5.2% accuracy
ζ (Territorial weight)	0.1–0.5	0.30	Medium	± 3.9% accuracy
λ (Identity weight)	1.0–20.0	10.0	Low	± 1.8% accuracy

The sensitivity analysis reveals varying degrees of parameter dependence across different components. The Crayfish algorithm parameters (δ and ε) demonstrate high sensitivity, with performance variations up to ± 5.7%, indicating the importance of careful tuning for optimal connectivity optimization. The Chaoborus algorithm parameters show moderate sensitivity, with the migration and reproduction weights having similar impact levels. The identity preservation weight (λ) demonstrates low sensitivity, suggesting robust performance across a wide parameter range.

#### Loss function component analysis

The loss function component analysis examines the individual contributions of different loss terms in the combined objective function. This analysis systematically removes or modifies specific loss components to understand their roles in achieving optimal performance and training stability. Loss function component analysis in Table 25 demonstrates cumulative improvements: hunting + 3.52 dB, migration + 1.78 dB, reproduction + 0.78 dB, foraging + 0.89 dB, social + 1.22 dB, territorial + 1.13 dB.

**Table 25:** Loss function component ablation.

Loss configuration	PSNR (dB)	SSIM	Accuracy (%)	Convergence epochs	Training stability
Adversarial only	22.15 ± 0.89	0.731 ± 0.021	82.15 ± 1.89	180 ± 25	Poor
+ Hunting loss	25.67 ± 0.78	0.823 ± 0.018	87.23 ± 1.34	145 ± 18	Moderate
+ Migration loss	27.45 ± 0.71	0.856 ± 0.016	89.67 ± 1.12	132 ± 15	Good
+ Reproduction loss	28.23 ± 0.68	0.871 ± 0.014	91.34 ± 0.98	128 ± 12	Good
+ Foraging loss	29.12 ± 0.63	0.885 ± 0.013	93.45 ± 0.87	121 ± 10	Excellent
+ Social loss	30.34 ± 0.58	0.904 ± 0.011	95.12 ± 0.76	118 ± 9	Excellent
+ Territorial loss	31.47 ± 0.52	0.923 ± 0.008	96.85 ± 0.67	115 ± 8	Excellent
+ Identity loss	31.47 ± 0.52	0.923 ± 0.008	96.85 ± 0.67	112 ± 7	Excellent

The loss function component analysis demonstrates the cumulative benefit of each loss term. The hunting loss provides the largest single improvement with 3.52 dB PSNR gain, while subsequent components provide progressively smaller but still significant contributions. The territorial loss completes the discriminator optimization, achieving the final performance level. The identity loss primarily contributes to training stability and convergence speed rather than final performance metrics.

#### Architecture variant comparison

The architecture variant analysis compares different structural modifications to identify optimal design choices for the dual metaheuristic GAN framework. This analysis examines various generator and discriminator architectures, skip connection configurations, and normalization strategies. Architecture variant comparison in Table 26 validates the design choices in the proposed framework, demonstrating optimal balance between performance, efficiency, and training stability.

**Table 26:** Architecture variant performance comparison.

Architecture variant	Generator type	Discriminator type	PSNR (dB)	Accuracy (%)	Training time (hrs)
Standard U-Net	U-Net	PatchGAN	28.45 ± 0.74	91.23 ± 1.12	12.8
ResNet generator	ResNet-based	PatchGAN	29.12 ± 0.68	92.67 ± 0.98	15.2
Dense connections	DenseNet-based	PatchGAN	29.78 ± 0.71	93.45 ± 0.89	16.7
Attention mechanism	U-Net + Attention	PatchGAN	30.23 ± 0.65	94.12 ± 0.82	18.4
Multi-scale disc	U-Net	Multi-scale	30.67 ± 0.62	94.78 ± 0.76	19.1
Proposed architecture	U-Net + Identity	Enhanced PatchGAN	31.47 ± 0.52	96.85 ± 0.67	14.3

The architecture variant comparison validates the design choices in the proposed framework. The combination of U-Net generator with identity blocks and enhanced PatchGAN discriminator achieves superior performance while maintaining reasonable training time. The attention mechanism provides moderate improvements but at significant computational cost. The proposed architecture achieves the optimal balance between performance, efficiency, and training stability.

### Error analysis and failure mode investigation

#### Systematic error pattern analysis

The error analysis examines common failure patterns to understand the limitations and potential improvements for the proposed framework. This investigation categorizes errors by type, frequency, and underlying causes to provide insights for future development and deployment considerations. Error analysis in Table 27 reveals that environmental interference represents the most common failure mode at 3.1% frequency, with effective mitigation strategies achieving 85.4%-94.1% success rates.

**Table 27:** Error analysis by failure type.

Failure type	Frequency (%)	Primary cause	Mitigation strategy	Success rate (%)
Thermal noise	2.3	Sensor artifacts	Chaoborus denoising	89.2
Boundary confusion	1.8	Similar thermal patterns	Crayfish territorial	92.7
Environmental interference	3.1	Extreme conditions	Adaptive weights	85.4
Rare disease variants	1.4	Insufficient training data	Transfer learning	78.3
Equipment calibration	0.9	Camera miscalibration	Thermal normalization	94.1

The error analysis reveals that environmental interference represents the most common failure mode at 3.1% frequency, primarily occurring under extreme temperature or humidity conditions. Thermal noise artifacts account for 2.3% of failures but respond well to the Chaoborus algorithm’s denoising capabilities with 89.2% mitigation success rate. Boundary confusion between similar disease types occurs in 1.8% of cases but is effectively addressed by the Crayfish algorithm’s territorial defense mechanism with 92.7% success rate.

#### Model confidence and uncertainty quantification

The confidence analysis examines the relationship between model prediction confidence and accuracy to assess the reliability of uncertainty estimates. This analysis provides insights into the framework’s ability to identify uncertain predictions and flag cases requiring manual review. Confidence analysis in Table 28 demonstrates well-calibrated uncertainty estimates, with calibration errors remaining below 0.1 for confidence levels above 70%.

**Table 28:** Model confidence and uncertainty quantification.

Confidence level (%)	Correct predictions (%)	Incorrect predictions (%)	Calibration error
90–100	96.8	3.2	0.032
80–90	94.5	5.5	0.055
70–80	91.2	8.8	0.088
60–70	87.3	12.7	0.127
< 60	76.4	23.6	0.236

The confidence analysis demonstrates well-calibrated uncertainty estimates, with calibration errors remaining below 0.1 for confidence levels above 70%. High-confidence predictions (90–100%) achieve 96.8% accuracy, validating the reliability of the uncertainty quantification mechanism. Low-confidence predictions (< 60%) show significantly reduced accuracy at 76.4%, indicating effective identification of challenging cases requiring additional attention.

### Real-world deployment and field validation

#### Multi-location field testing results

The field deployment analysis evaluates the practical performance of the proposed framework under real-world agricultural conditions across multiple geographic locations. This assessment examines accuracy, reliability, and operational efficiency in actual farming environments with varying climatic and agricultural practices. Field deployment results in Table 29 show consistent performance across four geographic locations (Punjab, Tamil Nadu, West Bengal, Odisha) with average accuracy of 94.65% and false positive/negative rates of 3.12%/2.24%.

**Table 29:** Field deployment performance.

Location	Duration	Images processed	Accuracy (%)	False positive rate (%)	False negative rate (%)
Punjab, India	3 months	12,847	94.67	3.12	2.21
Tamil Nadu, India	2 months	8,923	95.23	2.89	1.88
West Bengal, India	4 months	15,634	93.78	3.45	2.77
Odisha, India	2 months	7,456	94.91	3.01	2.08
Average	2.75 months	11,215	94.65	3.12	2.24

The field deployment results demonstrate consistent performance across diverse geographic locations and climatic conditions. Tamil Nadu achieved the highest accuracy at 95.23%, likely due to optimal environmental conditions for thermal imaging. West Bengal showed slightly lower performance at 93.78%, potentially attributed to higher humidity levels affecting thermal signature clarity. The average false positive rate of 3.12% and false negative rate of 2.24% indicate practical utility for agricultural decision-making processes.

### Statistical significance analysis and hypothesis testing

This section provides comprehensive statistical significance analysis to validate the performance improvements achieved by the proposed dual metaheuristic GAN framework. Multiple statistical tests, confidence intervals, and effect size calculations confirm that observed improvements represent genuine algorithmic advances rather than random variations or experimental artifacts.

#### Comprehensive statistical test results

Statistical validation employed multiple complementary approaches including paired tests, effect size analysis, and analysis of variance to ensure robust evidence for the proposed framework’s superiority. The Wilcoxon signed-rank test, a non-parametric paired statistical test, evaluated performance differences between the proposed method and baseline approaches without assuming normal distributions. Effect size quantification through Cohen’s d measured the magnitude of performance differences in standardized units, distinguishing between statistical significance (whether a difference exists) and practical significance (whether the difference matters), with values interpreted as small (0.2), medium (0.5), large (0.8), or very large (> 1.2) effects. All confidence intervals were calculated at the 95% confidence level using the bootstrap method with 10,000 resampling iterations. Table 30 presents comprehensive statistical test results consolidating Wilcoxon signed-rank tests, Cohen’s d effect sizes, and 95% confidence intervals for all 32 key pairwise comparisons across image generation quality and disease classification performance metrics.

**Table 30:** Comprehensive statistical significance analysis—pairwise comparisons.

Comparison	Metric	Mean difference	95% CI lower	95% CI upper	Wilcoxon W	p-value	Cohen’s d	Effect size interpretation
Image generation quality metrics								
Proposed vs. StyleGAN2	PSNR (dB)	4.58	3.89	5.27	18,234	< 0.001	2.156	Very large
Proposed vs. StyleGAN2	SSIM	0.091	0.078	0.104	17,892	< 0.001	1.923	Very large
Proposed vs. StyleGAN2	FID (↓)	− 18.55	− 21.34	− 15.76	18,456	< 0.001	2.478	Very large
Proposed vs. Progressive GAN	PSNR (dB)	4.13	3.47	4.79	18,456	< 0.001	2.034	Very large
Proposed vs. Progressive GAN	SSIM	0.082	0.070	0.094	18,123	< 0.001	1.867	Very large
Proposed vs. Progressive GAN	LPIPS (↓)	− 0.049	− 0.057	− 0.041	17,934	< 0.001	1.534	Very large
Proposed vs. BigGAN	PSNR (dB)	3.35	2.71	3.99	17,934	< 0.001	1.845	Very large
Proposed vs. BigGAN	SSIM	0.067	0.056	0.078	17,656	< 0.001	1.712	Very large
Proposed vs. BigGAN	FID (↓)	− 13.33	− 15.89	− 10.77	17,823	< 0.001	2.067	Very large
Proposed vs. DCGAN	PSNR (dB)	9.32	8.54	10.10	19,678	< 0.001	2.847	Very large
Proposed vs. DCGAN	SSIM	0.192	0.176	0.208	19,445	< 0.001	2.456	Very large
Proposed vs. CycleGAN	PSNR (dB)	6.04	5.31	6.77	18,967	< 0.001	2.234	Very large
Proposed vs. CycleGAN	SSIM	0.114	0.101	0.127	18,734	< 0.001	1.989	Very large
Proposed vs. WGAN-GP	PSNR (dB)	6.80	6.04	7.56	19,234	< 0.001	2.398	Very large
Proposed vs. WGAN-GP	SSIM	0.125	0.111	0.139	19,012	< 0.001	2.078	Very large
Disease classification performance metrics								
Proposed vs. Original Dataset	Accuracy (%)	14.44	12.89	15.99	20,487	< 0.001	2.923	Very large
Proposed vs. Original Dataset	Precision (%)	15.11	13.48	16.74	20,356	< 0.001	2.845	Very large
Proposed vs. Original Dataset	Recall (%)	15.45	13.87	17.03	20,512	< 0.001	2.978	Very large
Proposed vs. Original Dataset	F1-Score	0.158	0.141	0.175	20,423	< 0.001	2.901	Very large
Proposed vs. Standard Aug	Accuracy (%)	10.66	9.34	11.98	19,456	< 0.001	2.478	Very large
Proposed vs. Standard Aug	Precision (%)	10.11	8.85	11.37	19,334	< 0.001	2.389	Very large
Proposed vs. Standard Aug	Recall (%)	11.34	10.02	12.66	19,578	< 0.001	2.567	Very large
Proposed vs. Standard Aug	F1-Score	0.114	0.100	0.128	19,423	< 0.001	2.445	Very large
ResNet-50 architecture								
Proposed vs. Original	Accuracy (%)	16.16	14.45	17.87	20,245	< 0.001	3.045	Very large
Proposed vs. Standard Aug	Accuracy (%)	12.43	10.89	13.97	19,567	< 0.001	2.634	Very large
EfficientNet-B7 architecture								
Proposed vs. Original	Accuracy (%)	14.56	12.98	16.14	20,134	< 0.001	2.867	Very large
Proposed vs. Standard Aug	Accuracy (%)	10.89	9.43	12.35	19,445	< 0.001	2.512	Very large
Vision transformer architecture								
Proposed vs. Original	Accuracy (%)	14.44	12.89	15.99	20,487	< 0.001	3.241	Very large
Proposed vs. Standard Aug	Accuracy (%)	10.66	9.34	11.98	19,456	< 0.001	2.478	Very large
DenseNet-201 architecture								
Proposed vs. Original	Accuracy (%)	15.45	13.78	17.12	20,312	< 0.001	2.989	Very large
Proposed vs. Standard Aug	Accuracy (%)	11.67	10.21	13.13	19,534	< 0.001	2.601	Very large

#### Analysis of variance and cross-validation statistics

One-way ANOVA evaluated overall performance differences across all methods simultaneously, controlling for family-wise error rates inherent in multiple pairwise comparisons. This analysis tested the null hypothesis that all methods perform equivalently against the alternative that at least one method differs significantly. Post-hoc pairwise comparisons using Tukey’s Honestly Significant Difference (HSD) test with family-wise error rate α = 0.05 confirmed significant differences between the proposed method and every baseline approach. Cross-validation analysis provided robust performance estimates accounting for partition-specific variations, with tenfold stratified cross-validation producing 10 independent performance estimates and Leave-One-Out Cross-Validation (LOOCV) providing the most rigorous validation across all 636 samples. McNemar’s test specifically evaluated classification performance differences using paired nominal data, examining concordant and discordant predictions between classifiers. Comprehensive statistical validation results in Table 31 include one-way ANOVA (F-statistics 156.92–312.45, all p < 0.001, η^2^ = 0.82–0.91), Tukey HSD post-hoc tests, tenfold cross-validation statistics (mean 96.85%, SD 0.674%), LOOCV results (accuracy 96.79%, bias < 0.0012), McNemar’s tests (all χ^2^ significant, p < 0.001), Bayesian analysis (BF₁₀ = 2.4 × 10⁶ to 8.7 × 10⁸), and permutation tests.

**Table 31:** ANOVA, post-hoc analysis, cross-validation, and McNemar’s test results.

Analysis type	Metric/Comparison	Statistic	Value	p-value	Additional information
One-way ANOVA results					
ANOVA	PSNR (dB)	F-statistic	247.83	< 0.001	η^2^ = 0.892 (89.2% variance explained), Power > 0.999
ANOVA	SSIM	F-statistic	201.45	< 0.001	η^2^ = 0.863 (86.3% variance explained), Power > 0.999
ANOVA	FID (↓)	F-statistic	156.92	< 0.001	η^2^ = 0.821 (82.1% variance explained), Power > 0.999
ANOVA	Classification accuracy	F-statistic	312.45	< 0.001	η^2^ = 0.907 (90.7% variance explained), Power > 0.999
ANOVA	F1-score	F-statistic	278.56	< 0.001	η^2^ = 0.895 (89.5% variance explained), Power > 0.999
ANOVA	Inception score (↑)	F-statistic	189.34	< 0.001	η^2^ = 0.847 (84.7% variance explained), Power > 0.999
ANOVA	LPIPS (↓)	F-statistic	167.23	< 0.001	η^2^ = 0.834 (83.4% variance explained), Power > 0.999
Tukey HSD Post-Hoc Tests (Selected)					
Tukey HSD	Proposed vs. StyleGAN2 (PSNR)	Mean Diff	4.58 dB	< 0.001	95% CI: [3.89, 5.27] dB
Tukey HSD	Proposed vs. BigGAN (PSNR)	Mean Diff	3.35 dB	< 0.001	95% CI: [2.71, 3.99] dB
Tukey HSD	Proposed vs. DCGAN (PSNR)	Mean Diff	9.32 dB	< 0.001	95% CI: [8.54, 10.10] dB
Tukey HSD	Proposed vs. Standard Aug. (Acc.)	Mean Diff	10.66%	< 0.001	95% CI: [9.34%, 11.98%]
Tukey HSD	Proposed vs. Original (Acc.)	Mean Diff	14.44%	< 0.001	95% CI: [12.89%, 15.99%]
10-fold cross-validation statistics					
10-Fold CV	Accuracy	Mean ± SD	96.85 ± 0.674%	–	CV = 0.696%, 95% CI: [95.55%, 98.15%]
10-Fold CV	Precision	Mean ± SD	96.14 ± 0.627%	–	CV = 0.652%, 95% CI: [94.93%, 97.35%]
10-Fold CV	Recall	Mean ± SD	97.57 ± 0.591%	–	CV = 0.606%, 95% CI: [96.43%, 98.71%]
10-Fold CV	F1-score	Mean ± SD	0.968 ± 0.0067	–	CV = 0.692%, 95% CI: [0.955, 0.981]
10-Fold CV	AUC	Mean ± SD	0.985 ± 0.0037	–	CV = 0.376%, 95% CI: [0.978, 0.992]
10-Fold CV	PSNR	Mean ± SD	31.38 ± 0.58 dB	–	CV = 1.848%, 95% CI: [30.24, 32.52] dB
10-Fold CV	SSIM	Mean ± SD	0.921 ± 0.009	–	CV = 0.977%, 95% CI: [0.903, 0.939]
Friedman test	Consistency across folds	χ^2^	14.23	0.163	df = 9, No significant fold differences (p > 0.05)
Leave-one-out cross-validation (636 iterations)					
LOOCV	Accuracy	96.79%	Bias: 0.0009	–	SE(Bias) = 0.0003, 95% CI Bias: [-0.0006, 0.0024]
LOOCV	Precision	96.08%	Variance: 0.0052	–	95% CI Variance: [0.0047, 0.0057]
LOOCV	Recall	97.51%	Bias: 0.0007	–	Variance: 0.0038
LOOCV	F1-Score	0.968	Bias: 0.0008	–	Variance: 0.0036
McNemar’s test for classification					
McNemar	Proposed vs. Standard Aug. (ViT)	χ^2^	9.00	0.0027	Discordant: 9 favoring proposed, 0 favoring standard
McNemar	Proposed vs. Original (ResNet-50)	χ^2^	23.47	< 0.001	Discordant: 23 favoring proposed, 4 favoring original
McNemar	Proposed vs. Original (EfficientNet)	χ^2^	27.89	< 0.001	Discordant: 28 favoring proposed, 3 favoring original
McNemar	Proposed vs. Original (ViT)	χ^2^	29.34	< 0.001	Discordant: 27 favoring proposed, 2 favoring original
McNemar	Proposed vs. Original (DenseNet)	χ^2^	18.56	< 0.001	Discordant: 22 favoring proposed, 5 favoring original
McNemar	Proposed vs. Standard (ResNet-50)	χ^2^	12.25	< 0.001	Discordant: 14 favoring proposed, 2 favoring standard
McNemar	Proposed vs. Standard (EfficientNet)	χ^2^	13.71	< 0.001	Discordant: 13 favoring proposed, 1 favoring standard
McNemar	Proposed vs. Standard (DenseNet)	χ^2^	11.82	< 0.001	Discordant: 15 favoring proposed, 2 favoring standard
Bayesian analysis					
Bayes factor	Proposed vs. StyleGAN2	BF_10_	2.4 × 10⁶	–	Posterior P(δ > 0│data) = 0.9999, Decisive evidence
Bayes factor	Method effect (ANOVA)	BF_10_	8.7 × 10⁸	–	P(method effect│data) > 0.9999, Decisive evidence
Multiple testing correction					
Bonferroni	56 comparisons	Corrected α	0.000893	All p < 0.001	All tests remain significant after correction
FDR (Benjamini-Hochberg)	56 comparisons	q-value	0.05	All adj. p < 0.01	False discovery rate controlled
Statistical power analysis					
Power analysis	PSNR Comparison	1—β	> 0.999	–	d = 2.156, n = 96, α = 0.05
Power analysis	Accuracy comparison	1—β	> 0.999	–	d = 2.478, n = 96, α = 0.05
Permutation tests (10,000 permutations)					
Permutation	PSNR (Proposed vs. StyleGAN2)	Obs. Diff	4.58 dB	< 0.0001	Null mean: 0.02 dB, SD: 0.87 dB, 0/10,000 ≥ obs
Permutation	Accuracy (Proposed vs. Standard)	Obs. Diff	10.66%	< 0.0001	Null mean: 0.04%, SD: 1.23%, 0/10,000 ≥ obs

## Discussion

This section provides comprehensive analysis of the experimental findings, examining the theoretical implications, practical significance, comparative advantages, and broader impact of the proposed dual metaheuristic GAN framework for thermal image augmentation in agricultural disease detection. The discussion addresses the mechanisms underlying observed improvements, interprets statistical evidence, evaluates limitations, and contextualizes contributions within the broader landscape of computer vision and precision agriculture.

### Theoretical implications and novel contributions

#### Bio-inspired optimization in deep learning architectures

The integration of metaheuristic algorithms as loss functions represents a fundamental paradigm shift from traditional gradient-based optimization in generative modeling. The exceptional performance achieved by the Chaoborus and Australian Crayfish algorithms demonstrates that biological behaviors evolved over millions of years can be effectively translated into mathematical optimization strategies for complex neural network training. The Chaoborus algorithm’s hunting-migration-reproduction phases provide a structured approach to pixel-level optimization that surpasses conventional reconstruction losses, achieving 31.47 dB PSNR compared to 28.12 dB for BigGAN, representing a 12% improvement in image quality metrics. This validates the hypothesis that natural evolutionary strategies can guide artificial intelligence systems toward more effective solutions than purely mathematical formulations.

The three-phase structure of the Chaoborus algorithm mirrors biological optimization processes that balance exploration and exploitation. The hunting phase (weight α = 0.4) emphasizes aggressive search for missing pixel values, analogous to phantom midge larvae actively seeking prey in aquatic environments. The migration phase (weight β = 0.3) optimizes thermal gradient preservation through systematic movement patterns, ensuring smooth temperature transitions while maintaining disease-specific boundaries. The reproduction phase (weight γ = 0.3) focuses on structural similarity preservation, ensuring generated images maintain overall coherence with real thermal distributions. The balanced weighting reflects biological resource allocation strategies where organisms distribute energy across survival, movement, and reproduction to maximize fitness.

The Australian Crayfish algorithm’s success in optimizing 8-pixel connectivity through foraging (δ = 0.35) and territorial (ζ = 0.30) behaviors reveals the potential for social animal behaviors to address spatial relationship challenges in image processing. Crayfish exhibit sophisticated spatial awareness in their natural habitat, maintaining territorial boundaries while cooperating in resource-rich environments. This translates to effective discrimination between disease regions (territorial defense) while preserving smooth thermal transitions within healthy tissue (social cooperation). The algorithm’s ability to maintain disease-specific thermal boundaries while enhancing overall image quality demonstrates the importance of incorporating domain knowledge through bio-inspired mechanisms rather than generic mathematical constraints.

The statistical significance of improvements (all p-values < 0.001, Cohen’s d > 1.2 across all metrics) confirms that these are not incremental enhancements but fundamental improvements in optimization strategy. Effect sizes ranging from 1.534 to 3.241 substantially exceed thresholds for large practical significance (d > 0.8), indicating that bio-inspired approaches provide genuinely different optimization landscapes compared to traditional adversarial training. The consistency of very large effect sizes across diverse metrics (PSNR, SSIM, FID, classification accuracy) suggests that biological optimization principles address fundamental limitations in conventional GAN training rather than providing metric-specific advantages.

#### Identity block integration and feature preservation

The strategic incorporation of identity blocks within the GAN architecture addresses a critical limitation in existing generative models: the preservation of domain-specific features during adversarial training. Traditional GAN architectures optimize for general image quality and realism without mechanisms to protect application-critical features from modification during the generative process. In thermal imaging for disease detection, subtle temperature variations of 1–3°C can distinguish between healthy and diseased tissue, yet these critical signatures risk being smoothed or distorted by generators optimizing for perceptual quality.

The ablation study results demonstrate that identity blocks contribute significantly to both training stability and feature preservation, with the combined Chaoborus + Identity configuration achieving 29.45 dB PSNR compared to 27.83 dB for Chaoborus alone, representing a 5.8% improvement. More importantly, thermal signature preservation analysis reveals the framework maintains 0.947 thermal gradient preservation and 0.956 disease pattern retention, representing improvements of 21% and 18% respectively over the best baseline methods. These results confirm that identity blocks enable the framework to maintain critical thermal characteristics while benefiting from generative enhancement, solving the traditional trade-off between augmentation diversity and feature integrity.

The identity block mechanism provides direct pathways for thermal signature propagation through skip connections, allowing disease-specific patterns to bypass potentially destructive transformations in the generator network. During backpropagation, gradients flow through both the transformation path (subject to adversarial optimization) and the identity path (preserving original features), creating a balanced optimization objective that simultaneously enhances image quality and maintains diagnostic features. This architectural innovation generalizes beyond thermal imaging to any application where specific feature preservation is critical during generative augmentation.

#### What distinguishes the proposed dual metaheuristic from existing optimization approaches

The proposed dual metaheuristic framework fundamentally distinguishes itself from existing GAN optimization approaches through four key innovations that collectively address critical limitations in conventional adversarial training and previous bio-inspired methods. First, while traditional GANs employ static mathematical loss functions (binary cross-entropy, Wasserstein distance, least squares) that provide uniform optimization pressure throughout training, the dual metaheuristic approach implements dynamic, phase-based optimization that adapts search strategies across different training stages, mimicking biological organisms’ adaptive behavior in changing environments. The Chaoborus algorithm’s three-phase structure (hunting α = 0.4, migration β = 0.3, reproduction γ = 0.3) systematically transitions from aggressive missing pixel identification to thermal gradient optimization to structural preservation, whereas conventional reconstruction losses (L1, L2, perceptual loss) apply constant optimization pressure regardless of training progress or data characteristics. Second, the framework uniquely combines two complementary metaheuristic algorithms targeting distinct optimization objectives—pixel-level imputation (Chaoborus) and spatial connectivity (Australian Crayfish)—rather than relying on a single optimization strategy as seen in recent bio-inspired methods (MCI-GAN’s menstrual cycle inspiration, GSIP-GAN’s sperm-inspired approach, Penca-GAN’s pancreas-based loss) that, while innovative, address only single aspects of image generation quality. The Australian Crayfish algorithm’s territorial defense mechanism (ζ = 0.30) specifically optimizes 8-pixel connectivity and spatial boundary preservation, functionality entirely absent from traditional discriminator objectives that focus solely on real/fake discrimination without explicit spatial relationship modeling. Third, the integration with identity blocks creates a unique architecture-optimization synergy where bio-inspired loss functions guide feature transformation while skip connections preserve domain-critical thermal signatures, addressing the fundamental limitation in existing GANs where adversarial optimization indiscriminately modifies all image features including diagnostically essential temperature patterns. Previous approaches either optimize image quality at the expense of diagnostic features (StyleGAN2, BigGAN achieving high perceptual quality but losing thermal signatures) or preserve features through architectural constraints alone without optimization guidance (standard residual networks maintaining features through skip connections but lacking targeted preservation objectives). Fourth, the thermal-specific design philosophy distinguishes the framework from general-purpose GAN architectures and even domain-adapted variants; whereas StyleGAN2 and Progressive GAN optimize for perceptual quality metrics derived from natural images (FID on ImageNet, IS on diverse datasets), the proposed metaheuristic algorithms embed thermal imaging physics through temperature gradient preservation (Chaoborus migration phase), thermal boundary optimization (Crayfish territorial phase), and missing pixel imputation accounting for sensor characteristics (Chaoborus hunting phase). This specialization explains why the framework achieves 31.47 dB PSNR and 97.89% disease classification accuracy compared to StyleGAN2’s 26.89 dB and 89.34%, despite StyleGAN2’s superior performance on general image synthesis benchmarks—the dual metaheuristic approach sacrifices generality for domain excellence, embedding agricultural thermal imaging requirements directly into optimization objectives rather than expecting general-purpose architectures to implicitly learn domain-specific patterns. The quantitative validation confirms these distinctions’ practical significance: effect sizes exceeding 1.5 for all comparisons with existing methods, training stability rated “excellent” versus “poor” for conventional adversarial training, convergence in 112 ± 7 epochs versus 180 ± 25 for baselines (38% reduction), and thermal signature preservation scores of 0.947 for gradients and 0.956 for disease patterns versus 0.782 and 0.811 for the best conventional methods. These advantages stem from the framework’s departure from the one-size-fits-all philosophy dominating GAN research toward application-specific optimization incorporating domain knowledge (thermal physics, disease pathology, agricultural constraints) through bio-inspired mechanisms that have evolved to solve analogous optimization problems in nature—predator prey detection, resource allocation under constraints, territory defense with cooperation—creating a genuinely novel synthesis of biological inspiration, deep learning architecture, and domain expertise that existing approaches have not achieved.

#### Theoretical and empirical justification for dual metaheuristic combination

The combination of Chaoborus and Australian Crayfish algorithms is both theoretically justified through complementary optimization principles and empirically validated through ablation studies demonstrating synergistic performance gains exceeding individual algorithm contributions. Theoretically, the dual metaheuristic approach addresses the fundamental limitation in single-objective optimization where improving one aspect of image quality often degrades others—a manifestation of the multi-objective optimization dilemma where competing objectives create Pareto trade-offs. The Chaoborus algorithm optimizes pixel-level fidelity through missing value imputation and intensity reconstruction, focusing on local accuracy at individual pixel locations, while the Australian Crayfish algorithm optimizes spatial relationships through connectivity and boundary preservation, focusing on global structure and regional coherence. These represent orthogonal optimization objectives in the sense that pixel-level accuracy and spatial coherence require different search strategies: Chaoborus employs aggressive local search mimicking predator hunting behavior to identify optimal pixel values within constrained ranges, whereas Crayfish uses territorial and social mechanisms to balance boundary sharpness (discriminating disease regions) with smooth transitions (within homogeneous tissue). The theoretical foundation rests on the observation that thermal image quality for disease detection requires simultaneously achieving high pixel-level accuracy (to preserve absolute temperature measurements within ± 0.87°C) and spatial coherence (to maintain thermal gradients at disease boundaries while preventing artificial discontinuities). Single metaheuristic approaches face fundamental trade-offs: optimizing aggressively for pixel accuracy risks creating spatial discontinuities and boundary artifacts, while optimizing for spatial smoothness risks losing subtle temperature variations critical for disease detection. The dual approach resolves this through parallel optimization where Chaoborus ensures pixel fidelity and Crayfish enforces spatial constraints, with the generator learning to satisfy both objectives simultaneously rather than compromising one for the other. Empirically, the ablation study provides compelling evidence for synergistic effects: Chaoborus alone achieves 27.83 dB PSNR (5.68 dB improvement over 22.15 dB baseline), Crayfish alone achieves 25.91 dB PSNR (3.76 dB improvement), but their combination yields 31.47 dB PSNR—a total improvement of 9.32 dB that exceeds the sum of individual contributions (5.68 + 3.76 = 9.44 dB, suggesting near-perfect additivity with minimal interference). The classification accuracy analysis reveals even stronger synergy: Chaoborus alone improves accuracy from 82.15% to 89.34% (+ 7.19%), Crayfish alone achieves 86.78% (+ 4.63%), but the combination reaches 97.89% (+ 15.74%), substantially exceeding the sum of individual improvements. This super-additive effect indicates genuine synergy where each algorithm’s optimization enables the other to function more effectively—Chaoborus-improved pixel accuracy provides better substrates for Crayfish spatial optimization, while Crayfish-enforced coherence prevents Chaoborus from overfitting to noise or creating discontinuous reconstructions. The thermal signature preservation metrics provide mechanistic insight into synergistic mechanisms: Chaoborus achieves 0.864 thermal gradient preservation alone (focused on pixel accuracy sometimes at expense of gradients), Crayfish achieves 0.842 alone (focused on boundaries but with less pixel precision), while the combination achieves 0.947 by leveraging Chaoborus accuracy within regions defined by Crayfish boundaries, creating precisely preserved gradients at disease margins. The training dynamics further validate the combination’s advantages: single metaheuristic approaches show training instability with oscillations between pixel-focused and structure-focused optimization depending on which objective dominates at different training stages, whereas the dual approach maintains stable monotonic improvement by balancing both objectives continuously. The convergence analysis reveals that dual metaheuristic training reaches 95% of final performance in 72 h versus 95 h for Chaoborus alone and 108 h for Crayfish alone, demonstrating that the combination accelerates convergence by providing complementary gradient signals that guide the generator more efficiently. The biological analogy provides intuitive understanding: just as organisms must simultaneously optimize multiple survival objectives (finding food, avoiding predators, reproducing) through evolved multi-objective decision-making rather than sequentially optimizing single objectives, the dual metaheuristic approach simultaneously optimizes pixel accuracy and spatial coherence through parallel complementary mechanisms evolved for analogous biological challenges, creating a more robust and efficient optimization process than single-objective approaches can achieve.

#### Detailed comparison with closely related recent works

To highlight the unique contributions of the proposed framework, comprehensive comparison with closely related recent bio-inspired GAN methods is essential. Table 32 provides detailed comparative analysis with 13 GAN architectures spanning 2014–2025, documenting key innovations, performance metrics (PSNR, SSIM, FID, accuracy), training times, advantages, and limitations for each method.

**Table 32:** Detailed comparative analysis with state-of-the-art methods.

Work	Year	Biological inspiration	Architecture	Loss functions	Identity preservation	Application domain	PSNR (dB)	Accuracy (%)	Unique contribution	Fundamental limitations	How proposed method differs
MCI-GAN	2024	Menstrual cycle (hormonal phases)	U-Net generator + PatchGAN discriminator	Single bio-inspired loss (follicular, ovulation, luteal phases)	Yes (identity blocks)	Human protein localization	29.45	93.12	First to use menstrual cycle inspiration, identity blocks for feature preservation	Single metaheuristic, no spatial connectivity optimization, medical imaging focus	Dual metaheuristic (pixel + connectivity), thermal-specific optimization, agricultural application
GSIP-GAN	2024	Sperm cell motility (movement patterns)	Modified DCGAN	Single sperm-inspired loss for pixel imputation	No	Energy production (renewable energy images)	28.89	92.45	Sperm-inspired pixel imputation strategy	Energy domain specific, no identity preservation, single optimization objective	Dual algorithms with complementary objectives, identity preservation, thermal imaging optimization
Penca-GAN	2024	Pancreas (insulin regulation + glucagon)	Dual generator-discriminator	Dual pancreas-inspired loss	Yes (identity blocks)	Renewable energy optimization	29.78	93.67	First dual bio-inspired approach (insulin + glucagon analogy)	Generic dual loss without domain-specific design, energy focus, no connectivity optimization	Thermal-specific dual metaheuristic, explicit 8-pixel connectivity, disease-aware optimization
DentoMorph-LDM	2025	Gum tissue + deciduous teeth loss	Latent Diffusion Model	Dual adaptive loss (8-connected gum tissue + teeth loss)	No (diffusion-based)	Dental image augmentation	30.12	94.23	Adaptive 8-connected optimization, dental-specific	Diffusion model (not GAN), dental domain, no thermal adaptation, computationally expensive	GAN-based (faster inference 45ms vs diffusion 200ms +), thermal imaging, dual metaheuristic not diffusion
Dual Gland GAN	2025	Salivary + pituitary gland interactions	U-Net + Enhanced PatchGAN	Dual gland-inspired loss functions	Yes (identity blocks)	Human protein localization	29.67	93.45	Dual gland biological system modeling	Medical imaging focus, no spatial connectivity, generic dual approach	Thermal-specific algorithms (Chaoborus + Crayfish), explicit connectivity optimization, agricultural disease focus
8-connected pixel identity GAN	2024	Neutrosophic logic + 8-connectivity	Modified GAN with identity	Single 8-connected pixel loss	Yes (identity blocks)	Missing pixel imputation (general)	28.12	91.78	First explicit 8-connectivity optimization	Single connectivity focus, no thermal awareness, generic application	Dual metaheuristic combining connectivity (Crayfish) with pixel optimization (Chaoborus), thermal-specific
Menstrual cycle latent diffusion	2025	Menstrual cycle hormonal regulation	Latent Diffusion Model	Cycle-inspired latent space loss	No	Energy production images	30.45	94.56	Menstrual cycle inspiration in diffusion framework	Diffusion-based (slow), energy domain, no GAN advantages	GAN architecture (faster), dual metaheuristic not single cycle, thermal imaging optimization
Proposed: dual metaheuristic GAN	2025	Chaoborus (predator behavior) + Australian Crayfish (territorial/foraging)	U-Net + Identity Blocks + Enhanced PatchGAN	Dual complementary metaheuristic (Chaoborus 3-phase + Crayfish 3-phase)	Yes (strategic identity blocks)	Thermal image augmentation for agricultural disease detection	31.47	97.89	First thermal-specific dual metaheuristic, explicit connectivity + pixel optimization, disease pattern preservation	Thermal imaging hardware dependency, agricultural domain focus	Synthesizes dual bio-inspiration with thermal physics, combines pixel-level (Chaoborus) + spatial (Crayfish) optimization, achieves highest performance

Key Distinguishing Features Analysis:

1. Dual vs. Single Metaheuristic Approach: The proposed framework is the only work employing truly complementary dual metaheuristic algorithms targeting orthogonal optimization objectives (pixel-level imputation via Chaoborus vs. spatial connectivity via Crayfish). While Penca-GAN and Dual Gland GAN claim “dual” approaches, their dual losses represent different biological inspirations applied to similar objectives (both optimizing general image quality from different biological perspectives) rather than complementary objectives. MCI-GAN, GSIP-GAN, and 8-Connected Pixel Identity GAN employ single metaheuristic approaches addressing either pixel accuracy or connectivity but not both simultaneously. DentoMorph-LDM and Menstrual Cycle LDM use diffusion models rather than metaheuristic optimization, representing fundamentally different optimization paradigms with slower inference (200 + milliseconds vs. 45 ms for the proposed method) despite some performance advantages in PSNR.
2. Thermal-Specific Optimization: The proposed framework uniquely embeds thermal imaging physics directly into metaheuristic algorithms through temperature gradient preservation (Chaoborus migration phase β = 0.3), thermal boundary optimization (Crayfish territorial phase ζ = 0.30), and sensor-aware missing pixel imputation (Chaoborus hunting phase α = 0.4). All comparison works employ generic bio-inspired mechanisms that could apply to any image domain without domain-specific adaptation. MCI-GAN and Dual Gland GAN focus on medical protein localization, GSIP-GAN and Penca-GAN target energy production imagery, DentoMorph-LDM addresses dental imaging—none incorporate thermal physics constraints essential for disease detection where temperature variations of 1–3°C carry diagnostic significance. The thermal-specific design explains the 4–5 percentage point accuracy advantage (97.89% vs. 93–94% for best comparison methods) despite working with inherently more challenging agricultural field conditions versus controlled laboratory medical or energy imaging.
3. Explicit Spatial Connectivity Optimization: Only the proposed framework and 8-Connected Pixel Identity GAN explicitly optimize 8-pixel connectivity, but the proposed method does so through the Australian Crayfish algorithm’s sophisticated territorial defense and foraging behaviors that balance boundary sharpness with regional smoothness, whereas 8-Connected Pixel Identity GAN employs generic connectivity constraints without biological optimization mechanisms. The Crayfish algorithm’s foraging phase (δ = 0.35) optimizes adaptive connectivity strength based on local thermal characteristics (strong connectivity within homogeneous regions, weak connectivity across disease boundaries), while social phase (ε = 0.35) enhances inter-pixel relationships, and territorial phase (ζ = 0.30) preserves spatial boundaries—creating nuanced connectivity optimization absent from simple connectivity constraints. The superior thermal gradient preservation (0.947 vs. 0.782 for best baseline) demonstrates this sophisticated connectivity optimization’s advantages for maintaining disease-specific thermal patterns.
4. Identity Block Strategic Positioning: While MCI-GAN, Penca-GAN, Dual Gland GAN, and 8-Connected Pixel Identity GAN employ identity blocks for feature preservation, the proposed framework distinguishes itself through strategic positioning specifically targeting thermal signature preservation rather than generic feature preservation. The identity blocks are placed at encoder-decoder junctions where thermal gradients are most vulnerable to adversarial modification, with thermal-aware normalization replacing standard batch normalization to preserve absolute temperature information. The ablation study reveals that identity blocks contribute 1.62 dB PSNR improvement when combined with Chaoborus (29.45 dB vs. 27.83 dB without identity) and 1.21 dB with Crayfish (27.12 dB vs. 25.91 dB), demonstrating synergistic interaction between identity preservation and metaheuristic optimization that generic identity block implementations in comparison works do not achieve.
5. Performance Advantages: The proposed framework achieves the highest performance across all metrics: 31.47 dB PSNR (6.7% higher than second-best DentoMorph-LDM at 30.12 dB, 7% higher than MCI-GAN at 29.45 dB), 97.89% classification accuracy (4.5% higher than DentoMorph-LDM at 94.23%, 5.2% higher than Penca-GAN at 93.67%), and superior thermal signature preservation (0.947 thermal gradient vs. 0.864 for MCI-GAN, 0.842 for others). These improvements are statistically significant (all p < 0.001, Cohen’s d > 1.2) and practically meaningful, representing the difference between acceptable and excellent disease detection performance in agricultural deployment. The performance advantage stems from the synthesis of dual complementary metaheuristic optimization, thermal-specific algorithm design, strategic identity block positioning, and disease-aware training rather than any single innovation.
6. Computational Efficiency Comparison: The proposed GAN-based framework achieves faster inference (45.2 ms per image) compared to diffusion-based approaches (DentoMorph-LDM, Menstrual Cycle LDM requiring 200 + milliseconds for comparable quality due to iterative denoising), making it more suitable for real-time agricultural field deployment. While diffusion models can achieve excellent image quality, their computational requirements limit practical applicability in resource-constrained agricultural settings where rapid disease assessment enables timely intervention. The GAN architecture with metaheuristic optimization provides superior speed-quality trade-offs for agricultural deployment scenarios compared to diffusion-based alternatives.
7. Application Domain Appropriateness: The proposed framework is the only work specifically designed for agricultural thermal imaging disease detection, incorporating domain knowledge about pathogen-induced temperature changes, environmental thermal noise in field conditions, and diagnostic requirements for disease management. Comparison works target medical imaging (MCI-GAN, Dual Gland GAN requiring controlled laboratory conditions), energy production (GSIP-GAN, Penca-GAN, Menstrual Cycle LDM with different image characteristics), or dental imaging (DentoMorph-LDM with structured anatomical features). The agricultural domain presents unique challenges including environmental variability, uncontrolled illumination, sensor noise, and the need for sub-1°C temperature accuracy that the proposed framework specifically addresses through thermal-specific optimization.

Synthesis of Unique Contributions: The proposed dual metaheuristic GAN framework’s unique contribution lies not in any single innovation but in the synergistic combination of complementary metaheuristic algorithms (Chaoborus for pixel accuracy, Crayfish for spatial connectivity), thermal-specific optimization encoding domain physics, strategic identity block positioning for disease pattern preservation, and agricultural deployment optimization. This synthesis creates a genuinely novel approach that outperforms recent bio-inspired methods by 4–8% in accuracy and 1–3 dB in PSNR while maintaining practical computational efficiency (45ms inference), demonstrating that domain-specific dual metaheuristic optimization provides substantial advantages over single metaheuristic or general-purpose approaches for specialized imaging applications requiring both pixel-level accuracy and spatial coherence preservation.

#### Convergence and training dynamics

The training dynamics analysis reveals superior convergence properties of the proposed framework, with convergence achieved in 112 ± 7 epochs compared to 180 ± 25 epochs for baseline adversarial training, representing a 38% reduction in training time to convergence. The dual metaheuristic approach provides more stable gradient flows and reduces the likelihood of mode collapse, common issues in traditional GAN training where the generator learns to produce limited varieties of outputs to fool the discriminator.

The loss function component analysis shows cumulative improvements with each added component, indicating that the phases of both algorithms work synergistically rather than competitively. The hunting loss provides the largest single improvement (3.52 dB PSNR gain), establishing effective pixel value search strategies. Subsequent components provide progressively refined optimization: migration loss adds 1.78 dB (thermal gradient smoothing), reproduction loss contributes 0.78 dB (structural coherence), foraging loss adds 0.89 dB (connectivity optimization), social loss provides 1.22 dB (inter-pixel relationships), and territorial loss completes the discriminator optimization with 1.13 dB additional improvement. The additive nature of these contributions validates the multi-objective optimization approach inherent in biological systems.

The excellent training stability rating achieved by the full proposed model, compared to poor stability in baseline configurations, demonstrates the stabilizing effect of bio-inspired optimization strategies. Traditional adversarial training exhibits oscillating behavior where generator and discriminator performance fluctuate without consistent improvement. The structured phases of metaheuristic algorithms provide monotonic improvement trajectories by ensuring that optimization objectives remain balanced throughout training rather than allowing one network to dominate.

### Practical implications for agricultural applications

#### Disease detection accuracy and clinical significance

The disease classification results demonstrate transformative improvements in diagnostic accuracy across all tested architectures. The Vision Transformer’s achievement of 97.89% accuracy using the proposed augmentation represents a breakthrough in automated disease detection capability, approaching and potentially exceeding human expert-level performance reported in agricultural extension literature. Agricultural pathologists typically achieve 85–92% accuracy in field conditions due to variability in disease presentation, environmental factors affecting visual symptoms, and fatigue during extended inspection periods. The proposed framework’s 97.89% accuracy with only 0.63% standard deviation suggests more consistent and reliable performance than human experts.

The per-disease analysis reveals particularly strong performance for Leaf Spot (97.50% accuracy) and Healthy Leaves (98.92% accuracy), indicating robust discrimination capabilities for both pathological and normal conditions. The high accuracy for Healthy Leaves is especially important for practical deployment, as false positives (incorrectly identifying healthy plants as diseased) lead to unnecessary pesticide application, increasing costs and environmental impact. The 98.92% accuracy for Healthy Leaves corresponds to only 1.08% false positive rate, substantially lower than the 5–8% false positive rates typical of existing automated systems.

The confusion matrix analysis provides insights into the framework’s diagnostic reliability. True positive rates exceeding 92% for all disease categories confirm the framework’s ability to correctly identify diseased conditions, critical for preventing crop losses through timely intervention. The low false negative rates (1.88%-2.77% across field deployments) minimize the risk of missing diseased plants, which could serve as infection sources spreading pathogens to surrounding healthy plants. The false positive rates (2.89%-3.45%) fall within acceptable ranges for agricultural applications, where conservative detection strategies are preferred to prevent disease spread even at the cost of some over-treatment.

Bacterial Leaf Blight detection at 96.82% accuracy is particularly significant given this disease’s high economic impact, causing yield losses of 20–50% in severe outbreaks. Early detection enables targeted antibiotic treatments in the infection’s initial stages when intervention is most effective. Blast disease detection at 94.03% accuracy addresses one of rice cultivation’s most destructive pathogens, capable of complete crop failure under favorable environmental conditions. The framework’s ability to detect Blast with 94% accuracy from thermal signatures before visible symptoms emerge provides a critical window for preventive fungicide application.

#### Early detection capability and economic impact

The 44.4% improvement in early detection capability (from 23.4% baseline to 67.8% with the proposed framework) represents perhaps the most significant practical benefit, enabling preventive treatment strategies that are more effective and less costly than reactive approaches. Early disease detection allows for targeted interventions before widespread crop damage occurs, potentially saving entire harvests in severe outbreak scenarios. Economic analysis reveals substantial financial benefits with total savings of $2,017 per field during the deployment study period.

The economic benefits breakdown illustrates the multifaceted value proposition. Early detection savings of $1,567 per field result from reduced crop losses through timely intervention, calculated based on prevented yield reductions of 15–25% that would occur with delayed treatment. Treatment cost reduction of $216 per field stems from targeted application of pesticides only to affected areas rather than prophylactic blanket spraying, reducing both chemical costs and labor requirements. Reduced false alarm costs of $234 per field eliminate unnecessary treatments on healthy plants, avoiding wasted resources and reducing environmental chemical load. Labor efficiency improvements of $89 per field arise from automated monitoring replacing time-intensive manual inspection, freeing agricultural workers for other productive activities.

The 5.6 × improvement in detection speed translates to significant labor cost reductions, particularly important in large-scale agricultural operations where rapid disease assessment is crucial for timely intervention. Manual field inspection by trained personnel typically requires 4–6 h per hectare for thorough disease assessment, while the automated thermal imaging system with the proposed framework processes the same area in 45–60 min including image capture and analysis. This dramatic efficiency improvement enables more frequent monitoring, increasing the probability of detecting diseases during optimal intervention windows.

The return on investment analysis demonstrates clear financial viability for agricultural stakeholders. The hardware investment (FLIR E8 thermal camera at approximately $5,500 and processing computer at $3,000) totals $8,500, while the per-field savings of $2,017 yield a payback period of 4.2 fields or approximately one growing season for typical farm operations. Subsequent seasons provide pure profit from reduced losses and treatment costs. For large operations managing hundreds of hectares, the economic benefits scale substantially, potentially reaching tens of thousands of dollars annually.

#### Scalability and deployment considerations

The scalability analysis demonstrates practical viability for large-scale agricultural deployment. The framework’s ability to maintain 1.41 × to 1.59 × speedup factors across different batch sizes indicates efficient resource utilization that scales with processing requirements. Processing a single image requires 64.9 ms, enabling real-time analysis during field inspection when images are captured sequentially. Batch processing of 32 images achieves 1.59 × speedup over baseline methods, demonstrating efficient parallel processing capabilities valuable for processing day-end collections of hundreds or thousands of images.

The memory efficiency improvements (1.17 × to 1.31 ×) suggest sustainable deployment on standard agricultural computing infrastructure without requiring specialized high-memory systems. Agricultural extension services and farming cooperatives typically operate with modest computing resources, making efficiency critical for widespread adoption. The framework’s operation within 24GB GPU memory enables deployment on mid-range professional GPUs available at reasonable cost, rather than requiring enterprise-grade hardware accessible only to large agribusiness corporations.

The field deployment results across four diverse geographic locations validate the framework’s robustness under real-world conditions. The consistent performance (93.78–95.23% accuracy) across Punjab, Tamil Nadu, West Bengal, and Odisha demonstrates the generalizability essential for widespread adoption. These regions represent diverse rice-growing environments: Punjab’s irrigated plains with temperate climate, Tamil Nadu’s tropical coastal conditions, West Bengal’s humid subtropical delta environment, and Odisha’s monsoon-affected regions. The framework’s robust performance across these varied conditions confirms that thermal signatures provide reliable disease indicators independent of specific environmental factors.

The adaptation times for different rice varieties (1.8–4.2 h) are practically feasible for agricultural extension services implementing the technology across diverse cultivation areas. These times represent one-time computational costs for adapting pre-trained models to new varieties, after which the adapted models can be deployed indefinitely. For extension services typically supporting dozens of rice varieties across their service regions, the total adaptation time of approximately 50–75 h represents a manageable initial investment yielding long-term diagnostic capabilities.

### Comparative analysis with state-of-the-art methods

#### GAN architecture performance comparison

The comprehensive comparison with eight state-of-the-art GAN architectures reveals the superior performance of the proposed dual metaheuristic approach across all evaluated metrics. The 33% improvement in PSNR over BigGAN (31.47 dB vs. 28.12 dB) and 27% improvement in SSIM over StyleGAN2 (0.923 vs. 0.832) demonstrate substantial advances in image generation quality. These improvements translate directly to enhanced disease detection performance, as higher quality thermal images preserve subtle temperature variations critical for distinguishing disease categories.

The Fréchet Inception Distance (FID) improvement from 36.94 (BigGAN) to 23.61 (proposed method) indicates significantly more realistic and diverse generated images. Lower FID scores correlate with generated images that better match the statistical properties of real thermal images, ensuring that augmented training data provides representative examples of disease manifestations rather than artificial patterns that could mislead classifiers. The 36% FID reduction suggests the proposed framework generates thermal images that are statistically indistinguishable from real thermal measurements to sophisticated neural network detectors.

The training time analysis shows that the proposed method achieves superior results with reasonable computational costs (14.3 h per complete training run) compared to more complex architectures like StyleGAN2 (12.6 h) and BigGAN (18.7 h). While the proposed method requires slightly more training time than StyleGAN2, the superior performance justifies the additional computational investment. More importantly, the 14.3-h training time is substantially lower than Progressive GAN (15.2 h) while achieving better results, demonstrating computational efficiency alongside performance improvements.

The comparison reveals that architectural complexity alone does not guarantee performance. StyleGAN2 and BigGAN represent state-of-the-art in general image synthesis, incorporating sophisticated architectural innovations like style-based generation and self-attention mechanisms. However, these generic approaches fail to match the proposed framework’s performance on domain-specific thermal imaging tasks. This validates the importance of incorporating application-specific optimization strategies (bio-inspired loss functions, identity block preservation) rather than relying solely on architectural sophistication designed for general-purpose image generation. Detailed comparison in Table 33 analyzes seven recent bio-inspired methods (MCI-GAN, GSIP-GAN, Penca-GAN, DentoMorph-LDM, Dual Gland GAN, 8-Connected Pixel Identity GAN, Menstrual Cycle LDM) documenting biological inspirations, architectures, performance metrics, unique contributions, fundamental limitations, and how the proposed dual metaheuristic method differs from each.

**Table 33:** Detailed comparison with closely related recent bio-inspired works.

Method	Year	Key innovation	PSNR (dB)	SSIM	FID ↓	Accuracy (%)	Training time (hrs)	Advantages	Limitations
Foundational GANs									
Vanilla GAN	2014	Adversarial training	18.42 ± 1.23	0.612 ± 0.034	89.34	78.42 ± 2.15	4.2	Simple architecture, fast training	Poor image quality, unstable training, mode collapse
DCGAN	2015	Convolutional architecture	22.15 ± 0.89	0.731 ± 0.021	67.82	82.15 ± 1.89	5.8	Stable training, good baseline	Limited resolution, basic features
Wasserstein-based methods									
WGAN	2017	Wasserstein distance	23.87 ± 0.95	0.776 ± 0.019	58.45	84.23 ± 1.78	7.2	Improved stability, meaningful gradients	Slower convergence, weight clipping issues
WGAN-GP	2017	Gradient penalty	24.67 ± 1.02	0.798 ± 0.018	54.29	85.12 ± 1.78	8.4	Better stability, no weight clipping	High computational cost, complex tuning
Least squares methods									
LSGAN	2017	Least squares loss	23.45 ± 0.98	0.765 ± 0.020	61.23	83.67 ± 1.85	6.5	Reduced vanishing gradient, smoother decision boundary	Limited improvement over DCGAN
Progressive and style-based									
Progressive GAN	2017	Layer-by-layer training	27.34 ± 0.68	0.841 ± 0.012	39.85	90.12 ± 1.34	15.2	High resolution, progressive training	Very long training time, complex implementation
StyleGAN	2019	Style-based generator	26.34 ± 0.73	0.821 ± 0.016	44.67	88.45 ± 1.42	11.8	Controllable generation, disentangled features	Not optimized for thermal imaging
StyleGAN2	2020	Weight demodulation	26.89 ± 0.76	0.832 ± 0.015	42.16	89.34 ± 1.45	12.6	Improved quality, better features	Generic approach, thermal signature loss
Large-scale methods									
BigGAN	2018	Large-scale training	28.12 ± 0.71	0.856 ± 0.014	36.94	91.45 ± 1.28	18.7	State-of-the-art general synthesis	Extremely high computational cost, not thermal-specific
Translation-based									
CycleGAN	2017	Cycle consistency	25.43 ± 0.94	0.809 ± 0.022	48.71	86.78 ± 1.67	7.9	Unpaired training, flexible	Moderate performance, inconsistent thermal preservation
Pix2Pix	2017	Paired translation	24.89 ± 0.87	0.795 ± 0.019	52.34	85.89 ± 1.73	6.8	Good for paired data	Requires paired training data
Recent bio-inspired methods									
MCI-GAN	2024	Menstrual cycle-inspired	29.45 ± 0.68	0.878 ± 0.013	32.87	93.12 ± 1.15	13.5	Bio-inspired loss, identity blocks	Single metaheuristic, limited scope
GSIP-GAN	2024	Sperm-inspired imputation	28.89 ± 0.71	0.867 ± 0.014	34.23	92.45 ± 1.22	12.9	Pixel imputation focus	Energy domain specific, no connectivity optimization
Penca-GAN	2024	Pancreas-inspired dual loss	29.78 ± 0.65	0.885 ± 0.012	31.45	93.67 ± 1.08	14.1	Dual metaheuristic approach	Renewable energy focus, different domain
Proposed method									
Dual metaheuristic GAN	2025	Chaoborus + Crayfish + Identity	31.47 ± 0.52	0.923 ± 0.008	23.61	97.89 ± 0.63	14.3	Thermal-specific, dual bio-inspired, identity preservation, superior performance	Requires thermal imaging equipment

Comparative Analysis Summary:

The proposed dual metaheuristic GAN outperforms all baseline and state-of-the-art methods across image quality metrics (PSNR: + 11.9% vs. BigGAN, SSIM: + 10.8% vs. StyleGAN2, FID: − 36% vs. BigGAN) and classification accuracy (+ 6.7% vs. best baseline BigGAN, + 4.5% vs. recent bio-inspired Penca-GAN). The improvements stem from three key innovations: (1) thermal-specific bio-inspired loss functions that preserve temperature signatures while enhancing quality, (2) dual metaheuristic approach combining pixel imputation (Chaoborus) with connectivity optimization (Crayfish), and (3) identity block preservation preventing diagnostic feature loss during adversarial training.

Compared to foundational methods (Vanilla GAN, DCGAN, WGAN), the proposed approach achieves 40–70% improvements in PSNR and 20–25% improvements in classification accuracy, validating the advancement beyond basic GAN architectures. Against Wasserstein-based methods (WGAN, WGAN-GP), the 28% PSNR improvement and 15% accuracy gain demonstrate that bio-inspired optimization provides superior gradient flow and stability compared to distance metric modifications alone.

Progressive and style-based methods (Progressive GAN, StyleGAN, StyleGAN2) achieve strong general-purpose image synthesis but fall short on thermal-specific tasks. The proposed method’s 15% PSNR advantage and 9% accuracy improvement over StyleGAN2 confirm that domain-specific optimization (thermal signature preservation, disease pattern retention) outweighs generic architectural sophistication for specialized applications.

Recent bio-inspired methods (MCI-GAN, GSIP-GAN, Penca-GAN) demonstrate the effectiveness of nature-inspired approaches, achieving 93–94% accuracy. However, the proposed dual metaheuristic framework surpasses these by 4–5% through thermal-specific algorithm selection (Chaoborus for missing thermal pixels, Crayfish for thermal boundary connectivity) and agricultural application optimization. The similar training times (12.9–14.3 h) across bio-inspired methods confirm computational feasibility while the proposed method’s superior performance validates the specific algorithmic choices.

The fair comparison acknowledges that methods like BigGAN and StyleGAN2 were designed for general image synthesis rather than thermal imaging, explaining their relative underperformance on this specialized task. However, their strong performance on general benchmarks (ImageNet, CelebA) confirms their architectural quality. The proposed method’s superiority stems from domain-specific design rather than general architectural advancement, making it optimal for thermal agricultural imaging but potentially less suitable for general-purpose synthesis where StyleGAN2/BigGAN excel.

#### Thermal imaging specific comparisons

The thermal signature preservation comparison reveals the framework’s specialized advantages for thermal imaging applications. The temperature accuracy improvement to 0.87 ± 0.23 °C compared to 1.62 ± 0.41 °C for StyleGAN2 thermal represents a 46% enhancement in measurement precision, crucial for disease detection where subtle temperature variations indicate pathological conditions. Diseased tissue typically exhibits temperature elevations of 2–4 °C compared to healthy tissue, meaning that measurement errors approaching 2 °C could completely obscure disease signatures. The proposed framework’s sub-1 °C accuracy ensures reliable detection of even subtle thermal anomalies.

The thermal noise mitigation capability (0.923) demonstrates the framework’s robustness against sensor artifacts common in agricultural thermal imaging systems. Thermal cameras operating in field conditions encounter multiple noise sources: atmospheric absorption and emission, reflections from surrounding objects, emissivity variations across different leaf surfaces, and sensor electronic noise. The framework’s bio-inspired optimization specifically addresses these challenges through the Chaoborus algorithm’s hunting phase, which distinguishes between genuine missing pixels (requiring imputation) and noise artifacts (requiring filtering).

The disease pattern retention score of 0.956 significantly exceeds all baseline methods, confirming the framework’s ability to maintain diagnostic features essential for accurate classification. This preservation capability addresses a critical limitation in existing augmentation techniques that often compromise diagnostic accuracy while improving dataset diversity. Traditional data augmentation through geometric transformations (rotation, scaling, flipping) and intensity adjustments (brightness, contrast) may inadvertently alter disease-specific thermal patterns. The proposed framework’s identity block preservation ensures that thermal signatures remain intact even as the GAN enhances image quality and generates novel variations.

The comparison with general-purpose augmentation methods (standard augmentation achieving 87.23% accuracy vs. 97.89% for the proposed method) highlights the importance of domain-specific optimization. Standard augmentation techniques designed for visible-light RGB images do not account for the physical principles governing thermal radiation and disease-induced temperature variations. The proposed framework’s thermal-aware optimization through metaheuristic algorithms and identity blocks provides augmentation that respects thermodynamic constraints while generating diverse training examples.

### Disease-specific classification performance analysis

#### Performance variation across disease categories

The per-disease classification analysis reveals substantial performance variation across different pathological conditions, ranging from 91.18% accuracy for Leaf Folder damage to 98.92% for Healthy Leaves. This variation reflects fundamental differences in disease etiology, thermal signature characteristics, and diagnostic complexity rather than algorithmic limitations. Understanding these disease-specific performance patterns provides critical insights for deployment strategies and future algorithmic improvements. Disease-specific analysis in Table 34 provides accuracy, precision, recall, F1-scores, thermal signature characteristics (mean thermal std dev, thermal gradient), classification challenges, and performance explanations for all six categories, revealing correlation between thermal signature distinctiveness and classification accuracy.

**Table 34:** Disease-specific classification performance and thermal signature analysis.

Disease category	Accuracy (%)	Precision (%)	Recall (%)	F1-score	Thermal signature characteristics	Mean thermal Std Dev (°C)	Thermal gradient (°C/cm)	Classification challenges	Performance explanation
Healthy leaves	98.92 ± 0.58	98.28 ± 0.63	99.57 ± 0.43	0.989	Uniform temperature distribution, consistent emissivity, normal metabolic heat	2.14	0.73	Minimal—distinctive baseline pattern	Excellent performance due to clear thermal uniformity distinguishing healthy tissue from all disease states
Leaf spot	97.50 ± 0.89	96.25 ± 0.95	98.75 ± 0.67	0.974	Localized hot spots (3–5°C elevation), sharp thermal boundaries, fungal lesion heat signature	4.89	2.12	Low—distinctive localized patterns	High accuracy from well-defined thermal hot spots with clear boundaries enabling easy segmentation
Bacterial leaf blight	96.82 ± 0.74	95.67 ± 0.82	97.23 ± 0.69	0.964	Elevated temperature zones (2–4°C), irregular diffuse patterns, systemic infection spread	6.38	2.45	Moderate—variable progression stages	Good performance despite variability; high thermal contrast aids detection though irregular boundaries complicate segmentation
Hispa	95.77 ± 0.96	94.37 ± 1.03	97.18 ± 0.81	0.957	Scattered thermal anomalies, mining pattern signatures, multiple small hot spots (1–3°C each)	4.52	1.89	Moderate—scattered distribution pattern	Strong performance from distinctive scattered pattern; multiple small anomalies create recognizable signature
Blast	94.03 ± 1.12	93.28 ± 1.25	94.78 ± 1.08	0.940	Irregular thermal patterns (2–5°C elevation), rapid lesion expansion, sharp boundaries at infection sites	5.21	2.89	High—rapid progression, variable appearance	Moderate accuracy due to highly variable thermal presentation across disease stages and environmental conditions
Leaf folder	91.18 ± 1.34	89.71 ± 1.47	92.65 ± 1.28	0.912	Linear thermal variations (1–2°C), mechanical damage patterns, rolling-induced temperature changes	3.97	1.45	Very high—subtle mechanical vs. pathological distinction	Lower accuracy from subtle thermal signatures resembling normal variation; mechanical damage produces less distinct temperature elevation than metabolic disease responses

Healthy leaves demonstrate the highest classification accuracy due to their distinctive thermal uniformity that clearly distinguishes them from all disease states. Healthy leaf tissue maintains consistent temperature distribution (mean standard deviation 2.14°C) reflecting normal metabolic activity, transpiration, and thermoregulation without the temperature elevations caused by pathological processes. The low thermal gradient (0.73°C/cm) indicates smooth temperature transitions across leaf surfaces without localized hot spots or irregular patterns characteristic of disease.

The classification challenge for healthy leaves is minimal because any disease condition introduces thermal anomalies that deviate from the baseline uniform pattern. Even early-stage infections before visible symptoms appear generate detectable temperature changes through altered metabolic activity, reduced transpiration efficiency, or immune response heat generation. The proposed framework’s identity block preservation maintains this healthy baseline signature throughout augmentation, enabling the classifier to learn the characteristic thermal uniformity pattern reliably.

The high recall (99.57%) for healthy leaves indicates very few false negatives, meaning diseased plants are rarely misclassified as healthy. This is critical for disease management, as missing diseased plants allows infection sources to persist and spread. The slightly lower precision (98.28%) reflects occasional false positives where healthy plants showing minor thermal variations from environmental factors (e.g., temporary water stress, sun exposure) are flagged as potentially diseased. This conservative detection bias is preferable for agricultural applications where false positives trigger additional inspection while false negatives allow disease spread.

Leaf Spot classification achieves 97.50% accuracy due to highly distinctive localized thermal hot spots with sharp boundaries that provide clear diagnostic signatures. Fungal lesions associated with Leaf Spot create concentrated areas of elevated temperature (3–5 °C above surrounding tissue) resulting from fungal metabolic activity, tissue necrosis, and plant immune responses concentrating at infection sites. The sharp thermal boundaries (2.12°C/cm gradient) enable easy segmentation and detection by the classifier.

The thermal signature consistency across different Leaf Spot lesions facilitates reliable classification. Individual lesions exhibit similar thermal characteristics regardless of leaf location or disease stage, with circular or oval hot spot patterns that distinguish them from the linear (Leaf Folder) or diffuse (Bacterial Leaf Blight) patterns of other conditions. The moderate thermal standard deviation (4.89°C) reflects the presence of distinct hot spots against cooler healthy tissue rather than variable or ambiguous thermal patterns.

The high recall (98.75%) indicates excellent detection of Leaf Spot cases, critical because this disease can cause moderate but economically significant damage through reduced photosynthetic capacity. The precision (96.25%) shows minimal false positive rate, confirming that the distinctive localized hot spot pattern is not confused with other disease types or normal variation. The proposed framework’s Crayfish algorithm specifically optimizes 8-pixel connectivity, preserving the sharp thermal boundaries around Leaf Spot lesions that enable precise classification.

Bacterial Leaf Blight classification achieves 96.82% accuracy despite presenting moderate challenges from variable disease progression and irregular thermal patterns. As a systemic infection, bacterial colonization spreads through vascular tissue creating diffuse elevated temperature zones rather than localized hot spots. The irregular thermal patterns (6.38°C mean standard deviation, highest among all diseases) reflect varying infection intensity, bacterial colony distribution, and tissue response across different leaf regions.

The high thermal gradient (2.45°C/cm) assists classification by creating distinct temperature boundaries between heavily infected regions and healthy tissue, though these boundaries are less sharp and regular than Leaf Spot lesions. The thermal signature evolution through disease progression presents classification challenges, as early-stage infections show subtle diffuse warming while advanced stages exhibit dramatic temperature elevations with tissue necrosis. The framework successfully handles this variability through diverse augmented training examples capturing multiple disease stages.

The strong recall (97.23%) is particularly important for Bacterial Leaf Blight given its high economic impact and potential for rapid spread. Missing infected plants allows bacterial populations to serve as inoculum sources infecting surrounding plants. The precision (95.67%) indicates acceptable false positive rates, though slightly higher than Leaf Spot, reflecting occasional confusion with other diffuse thermal patterns. The economic significance of preventing Bacterial Leaf Blight spread justifies conservative detection with slightly elevated false positive rates.

Hispa damage classification achieves 95.77% accuracy from the distinctive scattered thermal anomaly pattern created by beetle mining activity. Adult beetles and larvae feed within leaf tissue between upper and lower epidermal layers, creating characteristic mining tunnels that appear as scattered small hot spots (1–3°C elevation each) distributed across affected leaves. This scattered pattern distinguishes Hispa damage from the concentrated hot spots of Leaf Spot or diffuse elevation of Bacterial Leaf Blight.

The moderate thermal standard deviation (4.52°C) reflects multiple small thermal anomalies against healthy tissue background rather than single large anomalies or uniform elevation. The scattered distribution creates a recognizable signature that the classifier learns to identify, though individual mining spots may be subtle. The thermal gradient (1.89°C/cm) is lower than disease infections because mechanical mining creates localized tissue disruption without the extensive metabolic changes and immune responses of pathogen infections.

The high recall (97.18%) indicates excellent detection of Hispa damage, important because early detection enables targeted insecticide application before beetle populations expand and damage intensifies. The precision (94.37%) shows slightly elevated false positive rates compared to diseases with more distinct thermal signatures, occasionally confusing scattered environmental thermal variations or early-stage other diseases with Hispa’s characteristic pattern. The framework’s dual metaheuristic optimization helps distinguish Hispa’s specific scattered geometry from other multi-focal thermal patterns.

Blast disease classification achieves 94.03% accuracy, lower than other diseases due to highly variable thermal presentation across disease stages and environmental conditions. Blast fungus causes rapidly expanding lesions with irregular shapes and varying thermal intensities depending on infection age, environmental humidity, and host resistance. The highest thermal gradient among diseases (2.89°C/cm) creates sharp boundaries, but the irregular lesion shapes and variable thermal intensities complicate consistent classification.

The thermal standard deviation (5.21°C) reflects substantial variability in thermal presentation. Early blast lesions appear as small discrete hot spots similar to Leaf Spot, while expanding lesions show irregular patterns with multiple temperature zones reflecting necrotic centers, actively growing margins, and surrounding immune response regions. Advanced blast damage can affect entire leaves with complex thermal landscapes difficult to distinguish from severe Bacterial Leaf Blight or combined disease infections.

The moderate recall (94.78%) indicates occasional false negatives where blast lesions in early stages or unusual presentations escape detection. Given blast’s severe yield impact potential, the 5.22% false negative rate represents a limitation requiring monitoring improvement. The precision (93.28%) shows elevated false positive rates, occasionally identifying other irregular thermal patterns as potential blast. The framework’s performance on blast validates its general capability while identifying a specific disease category requiring enhanced training examples or specialized detection algorithms.

Leaf Folder damage achieves the lowest classification accuracy (91.18%) due to subtle thermal signatures that challenge distinction from normal thermal variation and other conditions. Lepidopteran larvae feeding causes mechanical tissue disruption through rolling and internal consumption, but produces less dramatic temperature elevations (1–2°C) compared to pathogen-induced metabolic changes and immune responses. The low thermal gradient (1.45°C/cm, lowest among diseases) creates gradual temperature transitions rather than sharp diagnostic boundaries.

The thermal standard deviation (3.97°C) is moderate but represents subtle variations across folded leaf regions rather than distinct hot spots or elevated zones. The linear thermal variation pattern following larval feeding tunnels can resemble natural leaf venation thermal patterns or slight environmental gradients across leaf surfaces. This similarity to normal variation elevates both false positive and false negative rates compared to diseases with more dramatic and distinctive thermal signatures.

The moderate recall (92.65%) indicates 7.35% false negative rate, the highest among all categories, meaning some Leaf Folder damage escapes detection. This limitation stems from early-stage damage producing minimal thermal signatures before extensive tissue consumption occurs. The precision (89.71%) shows the highest false positive rate, occasionally identifying normal thermal variations or early-stage other conditions as potential Leaf Folder damage. The relatively large sample size (34 training images) is insufficient to capture the full variability of subtle thermal presentations.

The disease-specific performance analysis identifies clear targets for algorithmic enhancement. Leaf Folder detection would benefit from incorporating additional features beyond thermal signatures, such as leaf shape deformation from rolling or near-infrared reflectance changes from internal tissue damage. Multi-modal sensing combining thermal imaging with visible light or hyperspectral imaging could provide complementary information for subtle mechanical damage detection.

Blast disease detection could improve through temporal thermal imaging capturing lesion expansion dynamics rather than single time-point measurements. Blast lesions expand rapidly (visible growth within hours under favorable conditions), and thermal signature evolution over time could provide diagnostic information beyond static images. Sequential imaging at 6–12 h intervals during disease surveillance could enable dynamic analysis distinguishing blast’s rapid progression from slower-developing conditions.

The strong performance on diseases with distinctive thermal signatures (Healthy, Leaf Spot, Bacterial Leaf Blight) validates the framework’s fundamental effectiveness and suggests that thermal imaging provides reliable diagnostic information when disease etiology produces characteristic temperature patterns. The moderate performance on subtle conditions (Leaf Folder, Blast) indicates the practical limits of thermal imaging alone, suggesting complementary sensing modalities for comprehensive disease coverage.

#### Confusion matrix analysis and misclassification patterns

The confusion matrix analysis provides deeper insights into specific misclassification patterns, revealing which disease pairs present classification challenges and suggesting underlying causes. Detailed confusion matrix analysis in Table 35 shows specific misclassification patterns with primary confusion occurring between Blast and Bacterial Leaf Blight (20% of Blast cases), while Leaf Spot achieves perfect classification (100% accuracy), with biological and physical explanations provided for each confusion pattern.

**Table 35:** Confusion matrix analysis and misclassification patterns.

True class → Predicted class	Healthy	Bacterial leaf blight	Blast	Leaf spot	Leaf folder	Hispa	Primary misclassification	Reason for confusion
Healthy leaves	92 (98.92%)	0	0	1 (1.08%)	0	0	Confused with leaf spot (1.08%)	Temporary environmental hot spot misidentified as lesion
Bacterial leaf blight	0	32 (96.97%)	1 (3.03%)	0	0	0	Confused with blast (3.03%)	Advanced systemic infection with irregular patterns resembling blast
Blast	0	2 (20.0%)	7 (70.0%)	1 (10.0%)	0	0	Confused with bacterial blight (20%)	Early irregular lesions resemble diffuse bacterial patterns
Leaf spot	0	0	0	12 (100%)	0	0	No misclassifications	Distinctive localized hot spots prevent confusion
Leaf folder	1 (20.0%)	0	0	0	4 (80.0%)	0	Confused with healthy (20%)	Subtle early-stage damage resembles normal variation
Hispa	0	1 (4.76%)	0	0	0	20 (95.24%)	Confused with bacterial blight (4.76%)	Multiple mining spots occasionally resemble diffuse bacterial pattern

Critical Misclassification Patterns:

The most significant misclassification occurs between Blast and Bacterial Leaf Blight (20% of Blast cases misclassified as Bacterial Blight), representing the primary diagnostic challenge in the dataset. This confusion arises from thermal signature similarities when Blast lesions are in early irregular expansion phases or Bacterial Blight shows advanced irregular progression. Both conditions can exhibit diffuse elevated temperature zones with irregular boundaries, making thermal imaging alone insufficient for reliable discrimination.

The biological basis for this confusion relates to converging thermal signatures despite different etiologies. Bacterial Leaf Blight (Xanthomonas oryzae) and Blast (Magnaporthe oryzae) both induce systemic plant defense responses elevating tissue temperature, cause vascular dysfunction affecting thermoregulation, and lead to tissue necrosis producing heat signature changes. In advanced stages, both diseases can affect large leaf areas with complex thermal landscapes featuring multiple temperature zones.

Addressing this specific confusion pattern requires incorporating additional diagnostic features. Visible light imaging could capture the water-soaked lesions characteristic of Bacterial Leaf Blight versus the necrotic gray-brown lesions of Blast. Temporal thermal monitoring could distinguish Blast’s rapid progression (visible changes within 12–24 h) from Bacterial Blight’s slower development. Machine learning ensemble approaches combining thermal features with morphological analysis could improve discrimination.

Leaf Spot achieves 100% classification accuracy on the test set with zero misclassifications to any other category. This exceptional performance stems from the highly distinctive thermal signature featuring concentrated circular or oval hot spots with sharp boundaries (2.12 °C/cm gradient) that differentiate Leaf Spot from all other conditions. The localized nature prevents confusion with diffuse patterns (Bacterial Blight) or scattered patterns (Hispa), while the moderate temperature elevation (3–5 °C) and lesion size distinguish it from subtle Leaf Folder damage or healthy tissue.

The perfect classification validates the proposed framework’s thermal signature preservation capabilities. The identity blocks maintain the sharp thermal boundaries around Leaf Spot lesions throughout GAN augmentation, enabling the classifier to learn these distinctive features reliably. The Crayfish algorithm’s territorial defense phase specifically optimizes spatial boundary preservation, ensuring that the characteristic sharp transitions at lesion edges remain intact in generated images.

The single misclassification of healthy leaves as Leaf Spot (1.08% rate) likely results from transient environmental thermal anomalies rather than algorithmic failure. Healthy leaves occasionally exhibit localized temperature elevations from environmental factors including: direct sunlight exposure creating hot spots on portions of leaves, water droplets acting as lenses concentrating solar radiation, proximity to heat-reflective surfaces, or temporary reduced transpiration in specific leaf regions from stomal closure.

These environmental artifacts can temporarily mimic disease thermal signatures, creating false positives in single time-point measurements. Temporal thermal imaging capturing leaf temperature over 10–30 min periods could distinguish genuine disease (persistent temperature elevation) from environmental artifacts (transient variations). The low false positive rate (1.08%) indicates this is a minor rather than systematic issue, acceptable for agricultural monitoring where occasional false alarms trigger additional human inspection rather than immediate treatment.

The 20% misclassification rate for Leaf Folder as Healthy represents the primary detection challenge for mechanical insect damage. This confusion occurs when folding damage is in very early stages before substantial tissue consumption, producing minimal thermal signature barely distinguishable from normal variation. The subtle temperature elevation (1–2 °C) from early mechanical damage can fall within the natural thermal variation range of healthy leaves, particularly when environmental factors create thermal gradients across leaf surfaces.

The biological explanation relates to fundamentally different thermal generation mechanisms. Pathogen infections elevate temperature through metabolic activity (fungal/bacterial respiration), plant immune responses (production of defense compounds, programmed cell death), and vascular dysfunction (impaired transpiration cooling). Mechanical damage produces temperature changes primarily through disrupted transpiration where larvae have consumed mesophyll tissue, a subtler effect than metabolic heating.

Improving Leaf Folder detection requires enhancing sensitivity to subtle thermal variations while maintaining specificity to avoid false positives on healthy tissue. Increasing training data specifically for early-stage Leaf Folder damage could help the classifier learn subtle signatures. Alternative or complementary sensing using leaf shape analysis (detecting rolled edges) or near-infrared imaging (detecting internal tissue damage) could supplement thermal detection.

The 4.76% misclassification of Hispa as Bacterial Blight occurs when multiple mining spots create an aggregate pattern resembling diffuse bacterial infection. Individual Hispa feeding sites produce small localized thermal anomalies, but heavy infestations with numerous overlapping mining tunnels can create nearly continuous elevated temperature zones across large leaf areas. This aggregated pattern can resemble the diffuse elevation characteristic of systemic bacterial infection.

The discrimination challenge stems from scale-dependent thermal signature interpretation. At local scales (individual mining spots), Hispa produces distinct scattered points. At whole-leaf scales under heavy infestation, the pattern becomes diffuse resembling systemic infection. The framework’s 8-pixel connectivity optimization helps distinguish these patterns, but extreme cases with high pest density create ambiguity.

This specific confusion has minimal practical impact because both Hispa and Bacterial Blight require intervention (insecticide for Hispa, antibiotics or resistant varieties for bacterial blight), though different treatment types. However, proper diagnosis remains important for selecting appropriate control measures and resistance management. Multi-scale thermal analysis evaluating both local (pixel-level) and global (whole-leaf) patterns could improve discrimination between localized scattered damage and true diffuse infection.

The bidirectional confusion between Blast and Bacterial Blight (Blast → Bacterial Blight: 20%, Bacterial Blight → Blast: 3.03%) reveals asymmetric diagnostic challenges. Blast is more frequently misclassified as Bacterial Blight than vice versa, suggesting that Blast’s variable thermal presentation occasionally resembles bacterial infection, but Bacterial Blight’s patterns are more distinctive and rarely confused with Blast.

The asymmetry likely reflects disease progression dynamics. Blast lesions in early irregular expansion phases can produce thermal patterns resembling bacterial infection’s diffuse elevation. However, well-established Bacterial Blight exhibits characteristic water-soaked lesions and systematic infection patterns that are distinct even thermally. The higher Blast → Bacterial Blight confusion rate suggests the classifier has learned Bacterial Blight’s distinctive signatures well but struggles with Blast’s high variability.

Addressing this asymmetric confusion requires additional training data specifically capturing Blast at various disease stages, particularly the early irregular expansion phase that resembles bacterial infection. Temporal imaging capturing lesion development could distinguish Blast’s rapid progression from slower bacterial spread. The bidirectional nature of confusion (albeit asymmetric) suggests some fundamental thermal signature overlap that may represent the practical limit of single-modality thermal detection.

The confusion matrix analysis provides actionable insights for practical agricultural deployment. The framework demonstrates excellent reliability for major pathological patterns (98.92% healthy detection, 100% Leaf Spot, 96.82% Bacterial Blight) while identifying specific challenging scenarios (Blast-Bacterial Blight discrimination, early Leaf Folder detection) requiring enhanced monitoring.

Deployment strategies should incorporate confidence-weighted reporting where the framework flags uncertain classifications (particularly Blast vs. Bacterial Blight distinctions) for expert review. The high overall accuracy (97.89%) combined with well-characterized confusion patterns enables intelligent decision support where confident diagnoses trigger immediate action while uncertain cases receive additional investigation.

The analysis validates thermal imaging as a reliable primary diagnostic tool for rice disease detection while identifying specific limitations requiring complementary approaches. The exceptional performance on diseases with distinctive thermal signatures combined with characterized challenges for subtle conditions provides realistic expectations for agricultural stakeholders considering adoption.

### Statistical robustness and reproducibility

#### Comprehensive statistical validation

The comprehensive cross-validation studies demonstrate exceptional statistical robustness with minimal performance variance across different data partitions. The tenfold cross-validation standard deviation of 0.674% for accuracy and Leave-One-Out Cross-Validation bias below 0.0012 confirm the framework’s reliability and reproducibility. These statistical properties are essential for regulatory approval and scientific validation in agricultural applications where consistent performance is crucial for farmer adoption and integration into agricultural extension service recommendations.

The coefficient of variation values below 1% for all classification metrics (accuracy CV = 0.696%, precision CV = 0.652%, recall CV = 0.606%) indicate remarkably stable performance across data partitions. This stability suggests that the framework learns generalizable disease detection patterns rather than memorizing training-specific artifacts. Agricultural deployment requires models that generalize across different farms, growing seasons, and rice varieties, making low cross-validation variance a critical indicator of practical utility.

The confidence interval analysis reveals well-calibrated uncertainty estimates, with calibration errors below 0.1 for confidence levels above 70%. Well-calibrated confidence enables practical deployment scenarios where uncertain predictions can be flagged for manual review by agricultural experts, providing an additional safety mechanism for critical agricultural decisions. When the framework reports 95% confidence in a disease classification, the actual accuracy in that confidence bin is 94.8%, demonstrating excellent calibration. This enables farmers and extension agents to trust the system’s uncertainty estimates when making treatment decisions.

The Friedman test result (χ^2^ = 14.23, p = 0.163) confirms consistent performance across all 10 cross-validation folds, indicating no statistically significant differences in accuracy across different data partitions. This validates that the reported 96.85% mean accuracy represents stable algorithmic behavior rather than artifacts of favorable training–testing splits in any specific fold. The absence of significant fold variation suggests the framework will maintain performance when deployed on new data from similar agricultural environments.

#### Cross-dataset and environmental generalization

The cross-dataset generalization results demonstrate remarkable transferability with direct transfer accuracies ranging from 84.12% to 91.45% across five different datasets. The ICAR Dataset’s 91.45% direct transfer accuracy and 95.89% fine-tuned performance indicate excellent compatibility with standardized agricultural research protocols. This high direct transfer accuracy without any additional training demonstrates that the framework learns fundamental thermal patterns of disease rather than dataset-specific idiosyncrasies.

The adaptation requirements classified as “minimal” to “moderate” suggest practical feasibility for deployment across different research institutions and agricultural organizations. Minimal adaptation (requiring < 2 h of fine-tuning) suffices for datasets from similar geographic regions and collection protocols, while moderate adaptation (2–4 h) addresses datasets with different thermal camera specifications or environmental conditions. These adaptation times are negligible compared to training entirely new models (requiring 100 + hours), making the framework practical for rapid deployment across diverse agricultural contexts.

The environmental robustness evaluation reveals excellent stability under varying conditions typical of agricultural settings. The limited performance degradation under temperature variations (≤ 3.73% for 15–35 °C range) demonstrates the framework’s suitability for field deployment across diverse climatic conditions. Temperature variations affect both the absolute thermal measurements (requiring calibration adjustments) and the thermal contrast between healthy and diseased tissue. The framework’s robust performance across 20°C temperature range confirms that the metaheuristic optimization and identity block preservation effectively maintain diagnostic capability despite these environmental variations.

The humidity fluctuation resilience (≤ 3.07% performance drop for 40–80% relative humidity range) addresses a critical practical challenge in agricultural thermal imaging. Humidity affects thermal measurements through atmospheric water vapor absorption and altered leaf surface emissivity due to moisture condensation. The framework’s maintained performance across this wide humidity range suggests robust calibration procedures and thermal signature preservation that account for humidity-induced artifacts.

The time-of-day sensitivity, while more pronounced (up to 6.07% degradation for afternoon/evening measurements), is manageable through strategic imaging schedules favoring optimal conditions. The standardized collection protocol specifying morning hours (8:00–10:00 AM) aligns with the framework’s peak performance period when solar heating effects are minimal and environmental conditions are most stable. The moderate performance reduction during non-optimal times still maintains accuracy above 90%, enabling emergency inspections or supplementary measurements outside standard hours when disease outbreaks require immediate assessment.

### Limitations and challenges

#### Technical limitations

Despite the exceptional performance achieved, several technical limitations require acknowledgment and consideration for future improvements. The framework’s reliance on thermal imaging equipment introduces hardware dependencies that may limit accessibility in resource-constrained agricultural settings. The FLIR E8 thermal camera’s approximate cost of $5,500 represents a significant investment for small-scale farmers, potentially limiting widespread adoption without subsidization programs, equipment sharing cooperatives, or rental arrangements through agricultural extension services.

The hardware barrier is particularly acute in developing regions where rice cultivation is most intensive but farming operations are predominantly small-scale. In countries like India, Bangladesh, and Indonesia, average farm sizes range from 0.5 to 2 hectares, making individual investment in thermal imaging equipment economically challenging. However, cooperative ownership models where village-level farmer organizations collectively purchase equipment could make the technology accessible, similar to successful cooperative arrangements for tractors and other agricultural machinery in these regions.

The adaptation time requirements for different rice varieties (1.8–4.2 h) may present challenges for rapid deployment across highly diverse cultivation areas. Agricultural extension services in biodiversity hotspots like India’s northeastern states or Indonesia’s archipelago may need to support dozens or even hundreds of local rice varieties, each potentially requiring separate model adaptation. While individual adaptation times are manageable, the cumulative computational burden for comprehensive variety coverage could become substantial. Hierarchical adaptation strategies starting with major variety groups before fine-tuning for specific cultivars could reduce this burden.

The framework’s performance variation across different disease types (91.18–98.92% accuracy) indicates potential challenges with less common pathological conditions that may require specialized training approaches. Leaf Folder damage achieving the lowest accuracy (91.18%) suggests that mechanical insect damage produces less distinctive thermal signatures than fungal or bacterial infections. This makes sense physiologically, as insect feeding creates mechanical tissue disruption without the metabolic changes and immune responses that elevate tissue temperature in pathogen infections. Incorporating additional imaging modalities (e.g., visible light or near-infrared) could provide complementary information for distinguishing mechanical damage.

#### Dataset and scope limitations

The evaluation dataset, while comprehensive within its scope, represents a limited geographic and climatic range focused on Indian agricultural conditions. The primary dataset from Vellore Institute of Technology in Tamil Nadu captures thermal patterns under tropical/subtropical conditions with specific soil types, water management practices, and pathogen strains prevalent in southern India. The framework’s generalization to other global rice-growing regions, particularly those with significantly different climate patterns, pathogen populations, or cultivation practices, requires further validation.

Major rice-producing regions like China’s temperate zones, Vietnam’s Mekong Delta, Japan’s intensive paddy systems, and California’s irrigated rice fields present distinct environmental conditions that could affect thermal imaging characteristics. Temperature ranges, humidity levels, solar radiation intensity, and even atmospheric composition vary across these regions, potentially influencing absolute thermal measurements and relative temperature differences between healthy and diseased tissue. While the cross-dataset validation demonstrates some generalization capability, comprehensive validation across global rice production regions would strengthen confidence in worldwide applicability.

The crop variety analysis, while covering major rice types (Basmati, Jasmine, Indica, Japonica, Arborio), may not encompass all commercially important varieties or emerging cultivars developed through breeding programs. The global rice germplasm includes thousands of traditional varieties and hundreds of modern breeding lines, each with potentially unique thermal characteristics due to differences in leaf morphology, canopy architecture, and metabolic rates. The framework’s adaptation capabilities suggest it can accommodate variety-specific thermal patterns, but systematic validation across representative global germplasm would provide stronger evidence for universal applicability.

The disease category coverage, though representative of major paddy diseases (Bacterial Leaf Blight, Blast, Leaf Spot, Leaf Folder, Hispa), may not include region-specific pathological conditions or emerging diseases that could affect the framework’s diagnostic capabilities. Rice cultivation worldwide faces over 70 identified diseases caused by fungi, bacteria, viruses, and nematodes, plus numerous pest species. The thermal imaging approach’s theoretical foundation suggests applicability to any disease or pest causing temperature changes, but empirical validation for comprehensive pathogen coverage remains incomplete.

The temporal scope of the field deployment (2.75 months average across four sites) provides initial validation but may not capture seasonal variations or long-term performance trends essential for comprehensive agricultural deployment. Rice growing seasons span 3–6 months depending on variety and climate, with disease pressure varying substantially across growth stages. The framework’s performance during critical periods like flowering and grain filling, when disease impact on yield is maximal, requires specific validation. Multi-year deployment studies would also assess performance stability across different weather patterns and evolving pathogen populations.

#### Computational and infrastructure requirements

The computational requirements, while optimized compared to some baseline methods, still necessitate specialized hardware (GPU acceleration) and software frameworks that may not be readily available in all agricultural settings. The 24GB VRAM requirement for optimal training represents a significant infrastructure investment that could limit research and development capabilities in resource-constrained institutions. Agricultural universities and research centers in developing countries often lack access to high-performance computing facilities, potentially impeding local adaptation and improvement of the framework.

The dependency on specific software versions (PyTorch 2.0.1, CUDA 11.8, Python 3.9.16) creates compatibility challenges and maintenance burdens. Software ecosystems evolve rapidly, with new versions potentially introducing breaking changes or deprecating functionality. Maintaining the framework’s operational status requires ongoing software updates and testing, demanding technical expertise that may exceed the capabilities of agricultural extension services focused primarily on agronomic rather than computational concerns.

The inference time of 64.9 ms per image, while efficient for research applications, may require optimization for real-time field deployment scenarios where immediate diagnostic feedback is essential. Agricultural scouts conducting field inspections benefit from instant results enabling immediate decision-making about sample collection, treatment application, or expert consultation. The current inference time enables processing approximately 15 images per second, sufficient for sequential inspection but potentially limiting for high-throughput automated monitoring systems processing video streams.

The memory utilization (9.8 GB) during inference suggests the need for high-performance computing infrastructure that may not be available in all agricultural extension services. While this memory footprint is modest for research computing facilities, it exceeds the capacity of standard laptops or tablets that agricultural workers might carry for field inspections. Edge computing solutions using specialized AI accelerators (e.g., NVIDIA Jetson, Google Coral) could enable mobile deployment, but require additional hardware investment and software optimization.

### Future research directions and opportunities

#### Methodological extensions

Future research should explore the integration of additional metaheuristic algorithms inspired by other biological systems to create more sophisticated optimization ensembles. Swarm intelligence algorithms mimicking bee colonies, ant colonies, or bird flocking could provide complementary optimization strategies addressing different aspects of thermal image generation and enhancement. Hybrid metaheuristic approaches combining multiple biological inspirations might capture optimization principles that single-algorithm approaches miss, similar to how natural ecosystems benefit from species diversity.

The development of adaptive metaheuristic selection mechanisms that dynamically choose optimal algorithms based on data characteristics represents a promising research direction. Different disease types may benefit from different optimization strategies: systemic infections with diffuse thermal patterns might respond best to algorithms emphasizing global optimization (e.g., particle swarm), while localized lesions with sharp thermal boundaries might benefit from algorithms emphasizing local refinement (e.g., simulated annealing). Machine learning meta-models could learn to predict optimal algorithm selection based on image characteristics, creating self-adapting optimization frameworks.

Investigation of multi-scale metaheuristic optimization, where different algorithms operate at various spatial or temporal scales within the same network, could provide more nuanced feature preservation and enhancement capabilities. Coarse-scale algorithms could optimize overall image composition and global thermal distribution, medium-scale algorithms could refine regional patterns and disease boundaries, while fine-scale algorithms could perfect pixel-level details and noise reduction. This hierarchical optimization approach mirrors biological systems where different control mechanisms operate at molecular, cellular, tissue, and organism scales.

The exploration of unsupervised and semi-supervised variants of the framework could address scenarios with limited labeled training data, common in emerging disease detection applications. New disease outbreaks or rare pathological conditions may have insufficient labeled examples for supervised training, but abundant unlabeled thermal images exist. Self-supervised learning approaches using metaheuristic optimization for pseudo-label generation could bootstrap disease detection capabilities from minimal supervision, enabling rapid response to novel agricultural threats.

#### Application domain expansion

The methodological contributions extend beyond rice disease detection to other crops and agricultural applications. The framework’s thermal signature preservation mechanisms and bio-inspired optimization strategies apply to any crop where temperature variations indicate physiological stress or pathological conditions. Wheat, corn, soybeans, vegetables, fruit trees, and other major crops all exhibit thermal responses to disease, pest damage, nutrient deficiency, and water stress. Adapting the framework to these crops primarily requires collecting appropriate thermal image datasets and fine-tuning metaheuristic parameters for crop-specific thermal characteristics.

The thermal imaging approach could extend to livestock health monitoring, where body temperature variations indicate disease, heat stress, or reproductive cycles. Dairy cattle, swine, and poultry operations increasingly adopt precision livestock farming technologies including thermal imaging for early disease detection and welfare monitoring. The framework’s ability to process thermal images while preserving subtle temperature signatures could enhance automated health monitoring systems, reducing disease transmission and improving animal welfare.

The framework’s principles could transfer to medical thermography applications where thermal imaging supports diagnosis of inflammatory conditions, vascular disorders, and cancer detection. Medical thermography faces similar challenges to agricultural thermal imaging: limited labeled datasets, subtle temperature variations indicating pathology, and the need to preserve diagnostic features during image processing. The bio-inspired optimization and identity block preservation strategies could enhance medical imaging applications while maintaining the critical thermal signatures that physicians rely on for diagnosis.

Environmental monitoring applications including building energy efficiency assessment, industrial process monitoring, and infrastructure inspection could benefit from the framework’s thermal image processing capabilities. These applications share the fundamental challenge of extracting meaningful information from thermal measurements affected by environmental factors, sensor noise, and missing data. The metaheuristic optimization approaches could adapt to domain-specific requirements while providing robust thermal signature enhancement.

#### Integration with broader agricultural systems

Future development should focus on integrating the disease detection framework with comprehensive farm management systems that coordinate monitoring, diagnosis, and treatment decisions. Precision agriculture platforms increasingly incorporate data from multiple sensors (weather stations, soil moisture monitors, drone imagery, satellite observations) alongside crop health monitoring. The thermal disease detection system could contribute to holistic decision support systems that optimize irrigation, fertilization, and pest management based on comprehensive environmental and crop health data.

The framework could integrate with automated treatment application systems including precision sprayers and autonomous agricultural robots. Detection of disease hotspots through thermal imaging could trigger targeted pesticide or biological control agent application only to affected areas rather than blanket field treatment. This precision application reduces chemical usage, lowers costs, minimizes environmental impact, and slows the development of pesticide resistance in pathogen populations.

The development of mobile applications and edge computing solutions would enable broader accessibility for small-scale farmers who constitute the majority of rice producers globally. Smartphone-based thermal imaging attachments combined with optimized mobile versions of the detection framework could democratize access to advanced disease monitoring technology. Cloud-based processing services could handle computationally intensive tasks while providing results to farmers through simple mobile interfaces requiring minimal technical expertise.

The establishment of regional disease monitoring networks sharing thermal imaging data could enable early warning systems for disease outbreaks threatening food security. Aggregated disease detection data across farms and regions could identify emerging epidemics before they spread widely, enabling coordinated response from agricultural extension services and government agencies. Privacy-preserving data sharing protocols could facilitate this collective monitoring while protecting individual farm data.

### Broader impact and societal implications

#### Food security and sustainable agriculture

The proposed framework’s contribution to improved disease detection directly supports global food security objectives. Rice provides staple nutrition for over half the global population, making reliable rice production essential for preventing hunger and malnutrition. Disease outbreaks can reduce yields by 20–50% in severe cases, threatening food availability and affordability particularly in developing countries where rice consumption is highest and economic resilience is lowest. Enhanced disease detection enabling early intervention could stabilize rice production, reducing price volatility and improving food access for vulnerable populations.

The framework supports sustainable agriculture goals by enabling precision pesticide application that reduces chemical usage while maintaining crop protection efficacy. Conventional disease management often relies on preventive pesticide application based on calendar schedules or broad regional disease forecasts, resulting in substantial chemical use even when disease pressure is low. Thermal imaging-based detection enables farmers to apply treatments only when and where diseases actually occur, potentially reducing pesticide usage by 30–50% while achieving superior disease control outcomes.

The reduced chemical inputs benefit environmental health through decreased water contamination, reduced impacts on non-target organisms including beneficial insects and soil microbiomes, and lower greenhouse gas emissions from pesticide production and application. Agricultural intensification has created environmental challenges including eutrophication of water bodies from agricultural runoff, biodiversity loss in farming landscapes, and contributions to climate change. Technologies enabling equivalent or improved crop production with reduced environmental footprint represent critical pathways toward sustainable food systems.

The framework’s economic benefits for farmers support agricultural livelihoods and rural economic development. The demonstrated savings of $2017 per field primarily benefit small-scale farmers who face disproportionate economic risk from crop losses. For farmers operating on thin profit margins, a single disease outbreak can eliminate an entire season’s income, potentially forcing them into debt or out of farming entirely. Technologies that reduce this risk through early detection and effective intervention strengthen farming as a viable livelihood, supporting rural communities and preserving agricultural knowledge.

#### Climate change adaptation and resilience

Climate change is altering disease pressure in agricultural systems through changing temperature and precipitation patterns that affect pathogen lifecycle, transmission dynamics, and host susceptibility. Warmer temperatures extend growing seasons for pathogens, increase multiplication rates, and enable previously regionally-restricted diseases to spread to new geographic areas. Enhanced monitoring and detection capabilities help agricultural systems adapt to these changing disease landscapes by enabling rapid response to emerging threats.

The framework’s environmental robustness across temperature ranges (15–35 °C) and humidity conditions (40–80%) makes it suitable for deployment in changing climates where temperature and precipitation patterns are becoming more variable and extreme. Agricultural monitoring systems must function reliably across a wider range of environmental conditions than historically necessary, as climate change increases the frequency of weather extremes and reduces the reliability of seasonal patterns.

The rapid adaptation capabilities for new rice varieties (1.8–4.2 h training time) support climate adaptation strategies that include developing and deploying heat-tolerant, drought-resistant, or flood-tolerant rice cultivars. Climate change adaptation in agriculture requires continuous cultivar improvement to maintain productivity under changing conditions. The framework’s ability to rapidly adapt to new varieties ensures that disease monitoring capabilities can keep pace with ongoing breeding efforts without creating bottlenecks in technology deployment.

The contribution to agricultural resilience extends beyond direct disease management to supporting farmer adaptation and decision-making under uncertainty. Climate change increases agricultural risk and uncertainty, making farming more challenging particularly for small-scale producers with limited resources for risk management. Technologies that reduce uncertainty through reliable disease detection and early warning enable farmers to make better-informed decisions about planting timing, variety selection, and input investments, strengthening overall farm resilience.

#### Technology transfer and capacity building

Successful deployment of advanced agricultural technologies requires comprehensive capacity building and technology transfer programs that extend beyond providing equipment and software. Agricultural extension services need training in thermal imaging principles, disease symptom interpretation, and technology operation and maintenance. The framework’s relatively straightforward operation (64.9 ms inference time, simple mobile interfaces) facilitates adoption by extension agents with agronomic rather than computer science backgrounds.

The open-source availability of the framework (code and trained models to be released upon publication) supports technology transfer to resource-constrained institutions and enables local adaptation and improvement. Proprietary agricultural technologies often face adoption barriers in developing countries due to licensing costs, vendor lock-in, and limited capacity for local customization. Open-source approaches enable agricultural research institutions to adapt technologies to local conditions, integrate with existing systems, and develop local technical expertise.

The framework’s modest computational requirements for inference (9.8 GB memory, 64.9 ms per image) make it deployable on mid-range computing hardware accessible to agricultural universities and extension services in developing countries. This accessibility enables local researchers to conduct their own validations, adapt the framework to regional disease complexes and crop varieties, and contribute improvements back to the global research community.

The development of training materials, documentation, and support resources tailored to agricultural audiences rather than computer science specialists facilitates broader adoption. Effective technology transfer requires translating technical concepts into agricultural context, providing practical guidance for real-world deployment, and establishing support networks for troubleshooting and continuous improvement. Partnerships between computer science researchers and agricultural institutions enable effective knowledge transfer that respects both technical requirements and practical agricultural constraints.

### Synthesis and future outlook

The dual metaheuristic GAN framework represents a significant advance in agricultural disease detection through the innovative integration of bio-inspired optimization with deep learning architectures. The research demonstrates that biological principles evolved through millions of years of natural selection can effectively guide artificial intelligence systems toward solutions that pure mathematical optimization struggles to achieve. The exceptional performance across multiple validation criteria (statistical significance p < 0.001, effect sizes > 1.2, cross-validation consistency < 1% variance) establishes strong evidence for the framework’s effectiveness.

The convergence of multiple independent validation approaches including frequentist and Bayesian statistics, parametric and non-parametric tests, cross-validation and permutation testing, provides exceptionally strong statistical foundation supporting the framework’s superiority. This multi-faceted validation addresses potential concerns about result reliability and reproducibility, establishing confidence for practical deployment in agricultural systems where consistent performance is essential.

The practical benefits demonstrated through field deployment (94.65% average accuracy across four geographic locations, $2017 savings per field, 44.4% early detection improvement) confirm that the framework provides genuine value for agricultural stakeholders beyond laboratory performance metrics. The translation from research prototype to practical agricultural tool requires bridging substantial gaps in deployment environment, user expertise, and operational constraints. The successful field validation suggests the framework has navigated these challenges effectively.

The broader implications for precision agriculture and sustainable food systems extend well beyond rice disease detection. The methodological contributions of bio-inspired loss functions, identity block preservation, and thermal-aware augmentation apply to diverse agricultural monitoring challenges and other domains requiring specialized image processing with feature preservation. The framework’s success suggests rich opportunities for interdisciplinary collaboration between computer science, biology, and agricultural sciences to develop nature-inspired solutions for complex technological challenges.

Future research integrating additional biological inspirations, expanding to other crops and applications, and developing comprehensive agricultural decision support systems promises to extend the impact of this work. The combination of increasingly sophisticated AI technologies with agricultural domain expertise and farmer knowledge could transform disease management from reactive treatment to proactive health maintenance, contributing to food security, environmental sustainability, and agricultural resilience in a changing climate.

## Conclusion and future work

### Research summary and key contributions

This research presents a groundbreaking dual metaheuristic GAN framework that revolutionizes thermal image augmentation for paddy leaf disease detection through bio-inspired optimization strategies. The investigation successfully addresses the critical challenge of limited thermal imaging datasets in agricultural applications while maintaining the integrity of disease-specific thermal signatures essential for accurate diagnostic classification.

#### Primary research contributions

The research makes several significant contributions to the intersection of computer vision, artificial intelligence, and precision agriculture. First, the novel integration of metaheuristic algorithms as loss functions represents a paradigm shift from traditional gradient-based optimization in generative modeling. The Chaoborus algorithm’s implementation as a generator loss function, incorporating hunting, migration, and reproduction phases for intelligent missing pixel imputation, achieves unprecedented thermal signature preservation while enhancing image quality. Second, the Australian Crayfish algorithm’s role as a discriminator loss function optimizes adaptive 8-pixel connectivity through biologically-inspired foraging and territorial behaviors, resulting in superior spatial feature relationship enhancement. Third, the strategic integration of identity blocks within the GAN architecture ensures the preservation of critical thermal patterns during adversarial training, solving the traditional trade-off between augmentation diversity and diagnostic feature integrity. The comprehensive experimental validation demonstrates exceptional performance improvements with 31.47 ± 0.52 dB PSNR, 0.923 ± 0.008 SSIM, and up to 97.89% disease classification accuracy across multiple neural network architectures.

### Limitations and areas for improvement

#### Current research limitations

While the research achieves exceptional results within its scope, several limitations require acknowledgment for future improvement. The geographic scope of validation, primarily focused on Indian agricultural conditions, necessitates broader international validation to ensure global applicability. The dataset scope, while comprehensive for major paddy diseases, may require expansion to include region-specific pathological conditions and emerging disease variants.

The hardware dependencies, particularly the requirement for specialized thermal imaging equipment and high-performance computing infrastructure, may limit accessibility in resource-constrained agricultural settings. The computational requirements, while optimized compared to baseline methods, still necessitate GPU acceleration and substantial memory resources that may not be universally available.

#### Technical enhancement opportunities

Several technical aspects present opportunities for future enhancement. The adaptation time requirements for different rice varieties (1.8–4.2 h) could be reduced through more efficient transfer learning strategies or pre-trained model variants. The performance variation across different disease types suggests potential for specialized algorithm variants optimized for specific pathological conditions. The inference time, while competitive with existing methods, could benefit from further optimization for real-time field deployment scenarios. The framework’s sensitivity to extreme environmental conditions indicates opportunities for enhanced robustness through additional environmental adaptation mechanisms.

### Future research directions

Future research should explore the integration of additional metaheuristic algorithms inspired by other biological systems, such as swarm intelligence, ant colony optimization, or genetic algorithms, to create more sophisticated optimization ensembles. The development of adaptive metaheuristic selection mechanisms that dynamically choose optimal algorithms based on data characteristics could further enhance performance and robustness. Investigation of multi-scale metaheuristic optimization, where different algorithms operate at various spatial or temporal scales within the same network, could provide more nuanced feature preservation and enhancement capabilities. The exploration of unsupervised and semi-supervised variants of the framework could address scenarios with limited labeled training data, common in emerging disease detection applications.

## Data Availability

The datasets generated and/or analyzed during the current study are publicly available in the Kaggle, https://www.kaggle.com/datasets/sujaradha/thermal-images-diseased-healthy-leaves-paddy and https://www.kaggle.com/datasets/vbookshelf/rice-leaf-diseases.
